# Software for the frontiers of quantum chemistry: An overview of developments in the Q-Chem 5 package

**DOI:** 10.1063/5.0055522

**Published:** 2021-08-23

**Authors:** Evgeny Epifanovsky, Andrew T. B. Gilbert, Xintian Feng, Joonho Lee, Yuezhi Mao, Narbe Mardirossian, Pavel Pokhilko, Alec F. White, Marc P. Coons, Adrian L. Dempwolff, Zhengting Gan, Diptarka Hait, Paul R. Horn, Leif D. Jacobson, Ilya Kaliman, Jörg Kussmann, Adrian W. Lange, Ka Un Lao, Daniel S. Levine, Jie Liu, Simon C. McKenzie, Adrian F. Morrison, Kaushik D. Nanda, Felix Plasser, Dirk R. Rehn, Marta L. Vidal, Zhi-Qiang You, Ying Zhu, Bushra Alam, Benjamin J. Albrecht, Abdulrahman Aldossary, Ethan Alguire, Josefine H. Andersen, Vishikh Athavale, Dennis Barton, Khadiza Begam, Andrew Behn, Nicole Bellonzi, Yves A. Bernard, Eric J. Berquist, Hugh G. A. Burton, Abel Carreras, Kevin Carter-Fenk, Romit Chakraborty, Alan D. Chien, Kristina D. Closser, Vale Cofer-Shabica, Saswata Dasgupta, Marc de Wergifosse, Jia Deng, Michael Diedenhofen, Hainam Do, Sebastian Ehlert, Po-Tung Fang, Shervin Fatehi, Qingguo Feng, Triet Friedhoff, James Gayvert, Qinghui Ge, Gergely Gidofalvi, Matthew Goldey, Joe Gomes, Cristina E. González-Espinoza, Sahil Gulania, Anastasia O. Gunina, Magnus W. D. Hanson-Heine, Phillip H. P. Harbach, Andreas Hauser, Michael F. Herbst, Mario Hernández Vera, Manuel Hodecker, Zachary C. Holden, Shannon Houck, Xunkun Huang, Kerwin Hui, Bang C. Huynh, Maxim Ivanov, Ádám Jász, Hyunjun Ji, Hanjie Jiang, Benjamin Kaduk, Sven Kähler, Kirill Khistyaev, Jaehoon Kim, Gergely Kis, Phil Klunzinger, Zsuzsanna Koczor-Benda, Joong Hoon Koh, Dimitri Kosenkov, Laura Koulias, Tim Kowalczyk, Caroline M. Krauter, Karl Kue, Alexander Kunitsa, Thomas Kus, István Ladjánszki, Arie Landau, Keith V. Lawler, Daniel Lefrancois, Susi Lehtola, Run R. Li, Yi-Pei Li, Jiashu Liang, Marcus Liebenthal, Hung-Hsuan Lin, You-Sheng Lin, Fenglai Liu, Kuan-Yu Liu, Matthias Loipersberger, Arne Luenser, Aaditya Manjanath, Prashant Manohar, Erum Mansoor, Sam F. Manzer, Shan-Ping Mao, Aleksandr V. Marenich, Thomas Markovich, Stephen Mason, Simon A. Maurer, Peter F. McLaughlin, Maximilian F. S. J. Menger, Jan-Michael Mewes, Stefanie A. Mewes, Pierpaolo Morgante, J. Wayne Mullinax, Katherine J. Oosterbaan, Garrette Paran, Alexander C. Paul, Suranjan K. Paul, Fabijan Pavošević, Zheng Pei, Stefan Prager, Emil I. Proynov, Ádám Rák, Eloy Ramos-Cordoba, Bhaskar Rana, Alan E. Rask, Adam Rettig, Ryan M. Richard, Fazle Rob, Elliot Rossomme, Tarek Scheele, Maximilian Scheurer, Matthias Schneider, Nickolai Sergueev, Shaama M. Sharada, Wojciech Skomorowski, David W. Small, Christopher J. Stein, Yu-Chuan Su, Eric J. Sundstrom, Zhen Tao, Jonathan Thirman, Gábor J. Tornai, Takashi Tsuchimochi, Norm M. Tubman, Srimukh Prasad Veccham, Oleg Vydrov, Jan Wenzel, Jon Witte, Atsushi Yamada, Kun Yao, Sina Yeganeh, Shane R. Yost, Alexander Zech, Igor Ying Zhang, Xing Zhang, Yu Zhang, Dmitry Zuev, Alán Aspuru-Guzik, Alexis T. Bell, Nicholas A. Besley, Ksenia B. Bravaya, Bernard R. Brooks, David Casanova, Jeng-Da Chai, Sonia Coriani, Christopher J. Cramer, György Cserey, A. Eugene DePrince, Robert A. DiStasio, Andreas Dreuw, Barry D. Dunietz, Thomas R. Furlani, William A. Goddard, Sharon Hammes-Schiffer, Teresa Head-Gordon, Warren J. Hehre, Chao-Ping Hsu, Thomas-C. Jagau, Yousung Jung, Andreas Klamt, Jing Kong, Daniel S. Lambrecht, WanZhen Liang, Nicholas J. Mayhall, C. William McCurdy, Jeffrey B. Neaton, Christian Ochsenfeld, John A. Parkhill, Roberto Peverati, Vitaly A. Rassolov, Yihan Shao, Lyudmila V. Slipchenko, Tim Stauch, Ryan P. Steele, Joseph E. Subotnik, Alex J. W. Thom, Alexandre Tkatchenko, Donald G. Truhlar, Troy Van Voorhis, Tomasz A. Wesolowski, K. Birgitta Whaley, H. Lee Woodcock, Paul M. Zimmerman, Shirin Faraji, Peter M. W. Gill, Martin Head-Gordon, John M. Herbert, Anna I. Krylov

**Affiliations:** 1 Q-Chem, Inc., 6601 Owens Drive, Suite 105, Pleasanton, California 94588, USA; 2 Research School of Chemistry, Australian National University, Canberra, Australia; 3 School of Chemistry, University of Sydney, Sydney, New South Wales, 2006, Australia; 4 Department of Chemistry, University of Southern California, Los Angeles, California 90089, USA; 5 Department of Chemistry, University of California, Berkeley, California 94720, USA; 6 Division of Chemistry and Chemical Engineering, California Institute of Technology, Pasadena, California 91125, USA; 7 Department of Chemistry and Biochemistry, The Ohio State University, Columbus, Ohio 43210, USA; 8 Interdisciplinary Center for Scientific Computing, Ruprecht-Karls University, Im Neuenheimer Feld 205, 69120 Heidelberg, Germany; 9 Department of Chemistry, Ludwig Maximilian University, Butenandtstr. 7, D-81377 München, Germany; 10 Hefei National Laboratory for Physical Sciences at the Microscale, University of Science and Technology of China, Hefei, Anhui 230026, China; 11 Department of Chemistry, Loughborough University, Loughborough, United Kingdom; 12 Department of Chemistry, Technical University of Denmark, Kemitorvet Bldg. 207, DK-2800 Kgs Lyngby, Denmark; 13 Institute of Chemistry, Academia Sinica, 128, Academia Road Section 2, Nangang District, Taipei 11529, Taiwan; 14 Department of Chemistry, University of Pittsburgh, Pittsburgh, Pennsylvania 15260, USA; 15 Department of Chemistry, University of Pennsylvania, Philadelphia, Pennsylvania 19104, USA; 16 Department of Physics and Materials Science, University of Luxembourg, L-1511 Luxembourg, Luxembourg; 17 Department of Physics, Kent State University, Kent, Ohio 44242, USA; 18 Department of Chemistry, University of Cambridge, Cambridge, United Kingdom; 19 Donostia International Physics Center, 20080 Donostia, Euskadi, Spain; 20 Materials Science Division, Lawrence Berkeley National Laboratory, Berkeley, California 94720, USA; 21 Department of Chemistry, University of Michigan, Ann Arbor, Michigan 48109, USA; 22 Department of Chemistry, Fresno State, Fresno, California 93740, USA; 23 COSMOlogic GmbH & Co. KG, Imbacher Weg 46, D-51379 Leverkusen, Germany; 24 School of Chemistry, University of Nottingham, Nottingham, United Kingdom; 25 Mulliken Center for Theoretical Chemistry, Institut für Physikalische und Theoretische Chemie, Beringstr. 4, 53115 Bonn, Germany; 26 Department of Physics, National Taiwan University, Taipei 10617, Taiwan; 27 Department of Chemistry and Henry Eyring Center for Theoretical Chemistry, University of Utah, Salt Lake City, Utah 84112, USA; 28 Department of Chemistry, The University of Texas Rio Grande Valley, Edinburg, Texas 78539, USA; 29 Department of Chemistry and Biochemistry, Kent State University, Kent, Ohio 44240, USA; 30 Department of Chemistry and Biochemistry, University of Notre Dame, Notre Dame, Indiana 46556, USA; 31 Department of Chemistry, Boston University, Boston, Massachusetts 02215, USA; 32 Department of Chemistry and Biochemistry, Gonzaga University, Spokane, Washington 99258, USA; 33 Department of Physical Chemistry, University of Geneva, 30, Quai Ernest-Ansermet, CH-1211 Geneva 4, Switzerland; 34 Institute of Experimental Physics, Graz University of Technology, Graz, Austria; 35 Centre d’Enseignement et de Recherche en Mathématiques Informatique et Calcul Scientifique (CERMICS), École des Ponts Paris Tech and Institut National de Recherche en Informatique et en Automatique (INRIA), 6 & 8 Avenue Blaise Pascal, Cité Descartes, Champs sur Marne, 77455 Marne-La-Vallée Cedex 2, France; 36 Department of Chemistry, Virginia Tech, Blacksburg, Virginia 24061, USA; 37 Department of Chemistry, Xiamen University, Xiamen 361005, China; 38 Stream Novation Ltd., Práter utca 50/a, H-1083 Budapest, Hungary; 39 Graduate School of Energy, Environment, Water and Sustainability (EEWS), Korea Advanced Institute of Science and Technology (KAIST), Daejeon 34141, Republic of Korea; 40 Department of Chemistry, Massachusetts Institute of Technology, Cambridge, Massachusetts 02139, USA; 41 Wavefunction, Inc., Irvine, California 92612, USA; 42 Department of Chemistry, Purdue University, West Lafayette, Indiana 47907, USA; 43 Department of Chemistry and Biochemistry, Florida State University, Tallahassee, Florida 32306, USA; 44 Department of Chemistry, Western Washington University, Bellingham, Washington 98225, USA; 45 Chemical Sciences Division, Lawrence Berkeley National Laboratory, Berkeley, California 94720, USA; 46 Department of Chemistry, University of Helsinki, P.O. Box 55 (A. I. Virtasen aukio 1), FI-00014 Helsinki, Finland; 47 Department of Chemistry, University of Minnesota, Minneapolis, Minnesota 55455, USA; 48 Department of Chemistry and Chemical Biology, Harvard University, Cambridge, Massachusetts 02138, USA; 49 Zernike Institute for Advanced Materials, University of Groningen, 9774AG Groningen, The Netherlands; 50 Department of Chemistry, Florida Institute of Technology, Melbourne, Florida 32901, USA; 51 Department of Chemistry, KU Leuven, Leuven, Belgium; 52 Department of Chemistry, Yale University, New Haven, Connecticut 06520, USA; 53 School of Electrical and Computer Engineering, University of Oklahoma, Norman, Oklahoma 73019, USA; 54 Institute for Physical and Theoretical Chemistry, University of Bremen, Bremen, Germany; 55 Department of Chemistry, Fudan University, Shanghai 200433, China; 56 Department of Chemical Engineering, University of California, Berkeley, California 94720, USA; 57 Laboratory of Computational Biophysics, National Institute of Health, Bethesda, Maryland 20892, USA; 58 Physics Division, National Center for Theoretical Sciences, National Taiwan University, 1, Sec. 4, Roosevelt Rd., Taipei 10617, Taiwan; 59 Faculty of Information Technology and Bionics, Pázmány Péter Catholic University, Práter str. 50/a, 1083 Budapest, Hungary; 60 Department of Chemistry and Chemical Biology, Cornell University, Ithaca, New York 14853, USA; 61 Department of Chemistry, University at Buffalo, State University of New York, Buffalo, New York 14260, USA; 62 Materials and Process Simulation Center, California Institute of Technology, Pasadena, California 91125, USA; 63 Department of Chemical Physics, University of Science and Technology of China, Hefei, Anhui, 230026, China; 64 Department of Chemistry, University of California, Davis, California 95616, USA; 65 Department of Physics, University of California, Berkeley, California 94720, USA; 66 Department of Chemistry and Biochemistry, University of South Carolina, Columbia, South Carolina 29208, USA; 67 Department of Chemistry and Biochemistry, University of Oklahoma, Norman, Oklahoma 73019, USA; 68 Department of Chemistry, University of South Florida, Tampa, Florida 33620, USA

## Abstract

This article summarizes technical advances contained in the fifth major release of the Q-Chem quantum chemistry program package, covering developments since 2015. A comprehensive library of exchange–correlation functionals, along with a suite of correlated many-body methods, continues to be a hallmark of the Q-Chem software. The many-body methods include novel variants of both coupled-cluster and configuration-interaction approaches along with methods based on the algebraic diagrammatic construction and variational reduced density-matrix methods. Methods highlighted in Q-Chem 5 include a suite of tools for modeling core-level spectroscopy, methods for describing metastable resonances, methods for computing vibronic spectra, the nuclear–electronic orbital method, and several different energy decomposition analysis techniques. High-performance capabilities including multithreaded parallelism and support for calculations on graphics processing units are described. Q-Chem boasts a community of well over 100 active academic developers, and the continuing evolution of the software is supported by an “open teamware” model and an increasingly modular design.

## I. INTRODUCTION

The era of electronic computing began with the “ENIAC” machine,^1^ developed at the University of Pennsylvania beginning in 1943, and the first commercial machines began to be produced around 1950. Although originally developed for military applications, molecular physics was not far behind.^2^ The existence of these machines in universities led to the first development of quantum chemistry software starting in the mid-1950s.^3^ Prognosticating on the future of electronic structure theory in his 1966 Nobel Lecture, Mulliken stated that^4^

… the era of computing chemists, when hundreds if not thousands of chemists will go to the computing machine instead of the laboratory for increasingly many facets of chemical information, is already at hand.

However, he did caution that

… at the present time the rapid progress which could be made even with existing machine programs is not being made, simply because available funds to pay for machine time are far too limited.

**FIG. 1.**
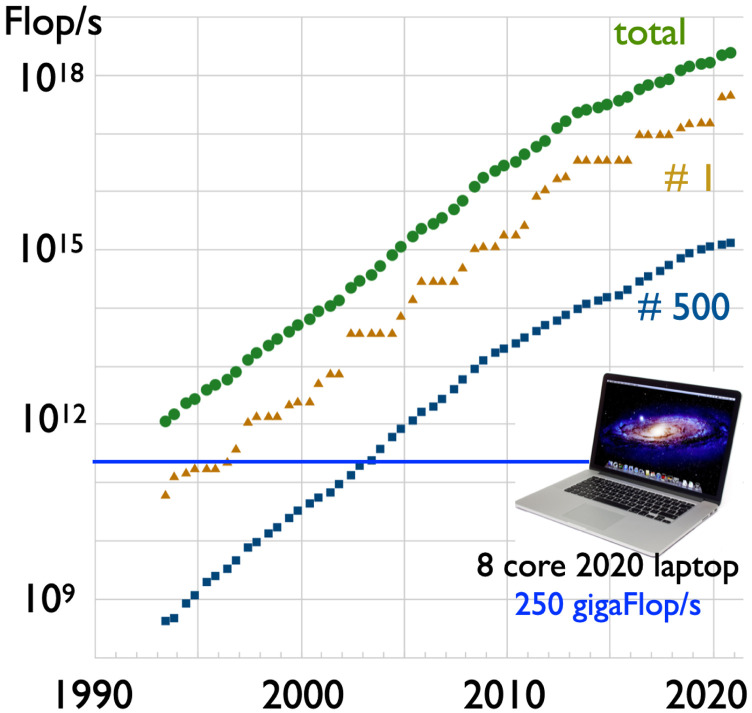
Development of leading edge computer capabilities, as documented through the performance of the world’s top 500 supercomputers, as measured on dense linear algebra in units of double precision floating-point operations per second (Flops/s). The data are adapted from Top500.org and compared against the performance of an eight-core laptop, which evidently has performance comparable to the world’s fastest supercomputer of the mid-1990s to late-1990s.

In the ensuing half-century, the problem of inadequate funds was resolved by the revolution in inexpensive computer hardware that traces its origin to the invention of the integrated circuit in the late 1950s and the microprocessor in the mid-1970s. Perhaps ironically, a desire for realistic simulation in computer games has led to such a massive market for high-performance hardware that today’s laptop computers have the power of the world’s most powerful supercomputer from the mid-1990s, as shown in Fig. 1. It is also worth noting that the roughly 100 W power consumption of today’s eight-core laptop is an impressive 5000× smaller than the corresponding supercomputer (e.g., the Fujitsu Numerical Wind Tunnel Computer, which was No. 1 in 1996, consumes 500 kW). At the other extreme, computing resources well into the terascale are routinely available on computer clusters, and leadership supercomputing is in the midst of a transition from petascale toward exascale computing.

This revolution in computer hardware is only meaningful to practicing chemists if corresponding software is available to enable straightforward and realistic simulation of molecules, molecular properties, and chemical reaction pathways. The first electronic structure codes were already working at the time of Mulliken’s Nobel address, and indeed, Charles Coulson had warned in 1959 of a growing split between theoretical chemists who were numerical simulators (primarily early code developers) and those who developed chemical concepts.^5^ Today one would rather say that quantum chemistry calculations are simulations whose results represent numerical experiments. Just like real experiments, results from these *in silico* experiments (even if reliable) must still be understood in conceptual terms, to the extent possible. The aspirations of early electronic structure codes are reflected in program names such as Polyatom,^6^ and such efforts rarely achieved useful accuracy or else did so via fortuitous cancellation of errors.^7^ However, today there are many useful program packages including ≈20 that are actively developed and supported.^8^

**FIG. 2.**
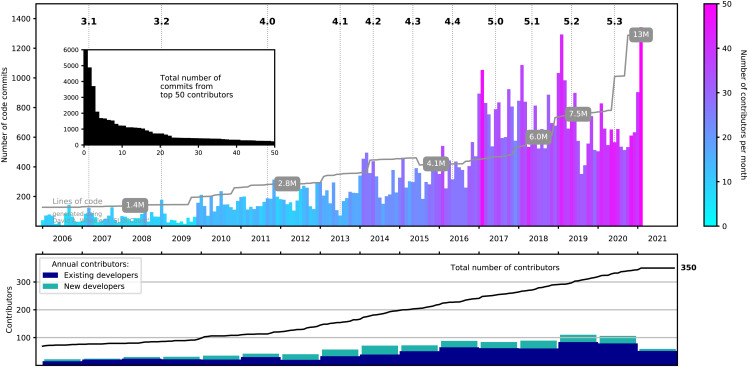
Statistics showing Q-Chem developer activity since 2006. Top: total number of code commits, organized chronologically by month. The color of each monthly entry indicates the number of individual developers who made commits. (Light blue is single-digit numbers, and the January 2021 peak represents about 50 developers committing code that month.) Bottom: growth of the developer base broken down into existing developers vs those who committed code for the first time. The inset depicts the total number of commits by the 50 most prolific developers.

One of those is the Q-Chem project, which began in the late 1992.^9^ Since its inception, Q-Chem has operated as a large collaboration that defines its genre as *open teamware* scientific software.^9,10^ The Q-Chem source code is open to a large group of developers that currently includes more than 100 individuals in at least 9 countries. Developers can submit their contributions for inclusion in the official releases as long as the changes do not violate the integrity of the overall package and are scientifically sound. In addition, several Q-Chem modules are distributed as open source software.^11–17^ Figure 2 illustrates some statistics regarding developer activity derived from the Q-Chem source code repository logs. These data provide clear evidence of the sustained growth of the developer community and the code itself over the past decade.

The Q-Chem collaboration has delivered useful and reliable quantum chemistry software over the course of five major releases (as documented in earlier review articles)^18–20^ and ≈15 minor releases. The present paper addresses progress made since 2015 by the relatively large team of academic developers and the relatively small team of professional programmers who contribute to the package. The authors of this paper^710^ represent contributors to Q-Chem v. 4 and v. 5, while contributors to earlier versions are recognized in overview articles describing v. 2,^18^ v. 3,^19^ and v. 4.^20^

The remainder of this paper is organized as follows: Sec. II provides an overview of density functional theory (DFT) capabilities in Q-Chem, including a survey of the 200+ exchange–correlation (XC) functionals that are presently available (Sec. II A).^21^ A variety of excited-state DFT capabilities are described in Sec. II C, including time-dependent (TD-)DFT in both its linear-response and its explicitly time-dependent (“real-time”) versions. Next, Sec. III describes single-reference correlated wave function methods and other many-body capabilities, while Sec. IV describes multireference methods. Section V highlights some specialty features, including methods for computing core-level (x-ray) excitation spectra, methods for describing metastable resonance states, methods for computing vibronic lineshapes, and finally the nuclear–electronic orbital (NEO) method for describing proton quantum effects. Section VI surveys methods for describing a molecule’s extended environment [e.g., quantum mechanics/molecular mechanics (QM/MM), dielectric continuum, and embedding methods]. Energy decomposition analysis methods are described in Sec. VII. Section VIII describes the Q-Chem software development environment, and Sec. IX provides an overview of high-performance capabilities, including multithreaded parallelism and algorithms that exploit graphics processing units (GPUs). Section X describes graphical user interfaces (GUIs). Finally, Sec. XI provides a wrap-up and a glimpse toward the future.

## II. DENSITY FUNCTIONAL THEORY

Standard quantum mechanics, including wave function-based quantum chemistry, employs an approximate *N*-electron wave function |Ψ⟩ to evaluate the energy, $E=\left\langle \Psi |\hat{H}|\Psi \right\rangle$. By contrast, DFT is based on the Hohenberg–Kohn theorems,^22–25^ which assert that the ground state energy *E* can be expressed as a functional of the electron density, *E* = *E*[*ρ*(**r**)]. While the exact functional is unknown and is almost certainly unknowable in explicit form, tremendous progress has been made toward achieving useful approximations. After some minimal background, this section summarizes recent aspects of that progress that are available in Q-Chem.

### A. Exchange–correlation functionals

**FIG. 3.**
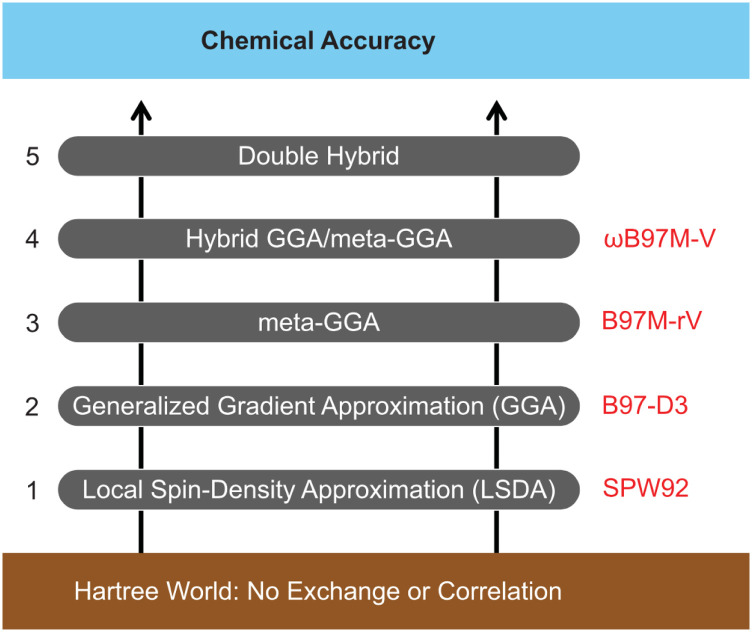
Illustration of the ladder-based classification of density functionals. Also shown at each rung are the top-performing functionals (out of 200 DFAs from rungs 1–4), as assessed using the MGCD84 database containing nearly 5000 data points.^21^ Adapted with permission from N. Mardirossian and M. Head-Gordon, Mol. Phys. **115**, 2315 (2017). Copyright 2017 Taylor and Francis.

Nearly all modern density functionals are of the Kohn–Sham type,^23–26^ in which the density is constructed from an auxiliary Slater determinant |Φ_*s*_⟩ composed of Kohn–Sham molecular orbitals (MOs), {*ϕ*_*k*_}. The determinant |Φ_*s*_⟩ describes a system of noninteracting electrons (or partially interacting electrons,^27^ for rungs 4 and 5 on the hierarchy in Fig. 3), which has the same density as the physical system of interest. This ensures so-called *N*-representability^24,25^ and is also used to exactly evaluate the noninteracting kinetic energy, $T_{s}=-\frac{1}{2}\left\langle \Phi_{s}|{\hat{\nabla }}^{2}|\Phi_{s}\right\rangle$. The Kohn–Sham DFT energy is expressed as

$$E=T_{s}+V_{\text{ext}}+E_{J}+E_{\text{XC}},$$ (1)

where the electron–nuclear attraction term (or “external potential,” *V*_ext_) and the classical Coulomb mean-field energy (*E*_*J*_) are known functionals of *ρ*(**r**). This leaves only the non-classical exchange–correlation (XC) energy (*E*_XC_) as unknown, and density functional approximations (DFAs) represent models for *E*_XC_.

Given a DFA, the energy is obtained by minimizing the energy of Eq. (1) with respect to the density $\rho \left(r\right)=\sum_{k}^{N}|\phi_{k}\left(r\right){|}^{2}$. This minimization is equivalent to solving the Kohn–Sham eigenvalue equation

$$\hat{F}\phi_{k}\left(r\right)=\epsilon_{k}\;\phi_{k}\left(r\right).$$ (2)

This is a one-electron analog of the time-independent Schrödinger equation. By analogy to the single-determinant Hartree–Fock approach in wave function theory (WFT),^28^ the effective one-electron Hamiltonian $\hat{F}\left[\left\{\phi_{k}\right\}\right]$ is known as the *Fock operator*, and it depends on its own eigenfunctions (as in Hartree–Fock theory). The power of Kohn–Sham DFT is that that the solution of the *self-consistent field* (SCF) problem in Eq. (2) would be equivalent to solving the full *N*-electron Schrödinger equation, if the exact functional *E*_XC_ were available.

While that is sadly not the case, the lack of an exact XC functional happily keeps electronic structure theorists gainfully employed, and there are many useful DFAs that far exceed the accuracy of the cost-equivalent Hartree–Fock method. The manner in which different DFAs depend on various descriptors of the density *ρ*(**r**) leads to five broadly recognized categories of density functionals that are commonly visualized as rungs of the metaphorical “Jacob’s ladder.”^29,30^ The rungs are illustrated in Fig. 3. From lowest to highest, the rungs correspond to the following:

1. Local Spin Density Approximation (LSDA). The LSDA functional *E*_XC_[*ρ*(**r**)] depends strictly on the density and solves the model problem of a uniform electron gas. Common fits to the uniform electron gas data are known as VWN^31^ and PW92,^32^ which are quite similar.^33^ Most higher rungs of Jacob’s ladder introduce corrections based on LSDA as a starting point.
2. Generalized Gradient Approximations (GGAs). GGAs add a dependence on $\hat{\nabla }\rho \left(r\right)$ to *E*_XC_, making the *ansatz* potentially exact for slowly varying electron densities, not just uniform ones. Many useful GGAs have been developed, including PBE,^34^ BLYP,^35,36^ and B97-D.^37^ Q-Chem 5 also includes the nonseparable gradient approximation, GAM.^38^ It is nowadays standard to add empirical dispersion corrections (of the D, D3, or D4 form, for example) to these functionals,^39^ in order to improve their performance for non-bonded interactions.
3. Meta-GGAs. These functionals incorporate an additional dependence on the kinetic energy density, *τ*(**r**). Functionals on this rung are still under active development and noteworthy recent meta-GGAs include SCAN,^40^ B97M-V,^41^ and revM06-L.^42^ The “-V” suffix in B97M-V indicates that the functional also includes a nonlocal correlation functional (VV10),^43^ which can (at least in principle) account for dispersion interactions for the right physical reasons,^44^ whereas “semilocal” functionals that depend only on *ρ*(**r**), $\hat{\nabla }\rho \left(r\right)$, and/or *τ*(**r**) lack the nonlocality to describe correlated density fluctuations between nonoverlapping densities.
4. Hybrid functionals. Hybrid DFAs include some portion of the “exact” (or Hartree–Fock) exchange energy associated with the Kohn–Sham determinant. The traditional approach has used a fixed fraction of exact exchange, and such functionals are known as “global” hybrid functionals. Popular examples include B3LYP,^35,36^ PBE0,^45^ and M06-2X,^46^ while some more recent and noteworthy examples of global hybrids include SCAN0,^47^ MN15,^48^ and revM06.^49^ A popular alternative to global hybrids uses a variable fraction of exact exchange that typically increases with the inter-electron distance, *r*_12_. These are known as *range-separated hybrid* (RSH) functionals, and notable older examples include *ω*B97X^50^ and *ω*B97X-D,^51^ while newer examples include *ω*B97X-V^52^ and *ω*B97M-V.^53^ More specialized RSH functionals are also widely used for time-dependent DFT calculations of excited states; see Sec. II C.
5. Double-Hybrid (DH) functionals. Hybrid DFAs depend only on the occupied Kohn–Sham orbitals, but DH-DFAs add an additional dependence on the virtual (unoccupied) Kohn–Sham MOs, which facilitates description of nonlocal electron correlation, as in second-order Møller–Plesset perturbation theory (MP2). DH-DFAs have undergone rapid recent development,^54,55^ and established models such as B2PLYP-D3,^56^ XYG3,^57^ and *ω*B97X-2^58^ have been joined by promising new DH-DFAs, including *ω*B97M(2),^59^ and a slew of functionals that involve empirical scaling of the MP2 spin components.^60–62^ Relative to the lower rungs of the ladder, the prospect of higher accuracy from DH-DFAs also comes with the cost of significantly higher computational demands, and significantly slower convergence of the results toward the complete basis set limit.

With respect to DFT, the most important feature of Q-Chem is that an exceptionally rich set of density functionals is supported: well over 200 functionals are available for a user to choose between.^21^ A closely related feature is that Q-Chem contains a very complete set of methods for accurate treatment of dispersion interactions. These include Grimme’s D,^37^ D3,^63,64^ and D4 corrections,^65^ as well as a variety of nonlocal correlation and van der Waals functionals,^43,66–68^ the exchange dipole model (XDM),^69,70^ the Tkatchenko–Scheffler (TS) model,^71^ and the many-body dispersion (MBD) model.^72–74^ In addition, for calculations on large molecules using the small def2-SVPD basis set,^75,76^ a built-in geometric counterpoise correction method (the so-called DFT-C approach^77^) is available. Q-Chem also has analytic nuclear gradients and Hessians for most of this long list of functionals through rung 4. Some modern DFAs are more challenging to integrate than older ones, and a set of modern quadrature grids is available,^78^ with sensible defaults.

This broad selection of available functionals is a perhaps unfortunate necessity due to the fact that the “best” functional often depends on the problem at hand. According to Pople’s concept of a *theoretical model chemistry*,^79,80^ one should validate candidate approximations using known results that are related (as closely as possible) to the desired area of chemical application and then proceed to make predictions for related but unknown systems. The best functional(s) for modeling hydrogen storage in a host material,^81^ for example, may differ significantly from the best functional(s) to describe elementary steps in a CO_2_ reduction catalyst,^82^ or the best functional may even differ from one catalyst to another,^83^ as dictated by the need to get reduction potentials in reasonable agreement with experiment. (Excited-state calculations bring in a host of other considerations,^84–89^ as discussed in Sec. II C.) Problem-specific validation of the choice of DFA for a given application is therefore a good idea, particularly if there are good available data to benchmark several candidate DFAs.

To bring some order to this situation, it is important to recognize that there are general classes of energy differences that are common to most applications in chemistry. Such classes include non-covalent interactions, thermochemical energy differences, isomerization energies, and reaction barrier heights. The large main-group chemistry database (MGCDB84) developed by Mardirossian and Head-Gordon is categorized along these lines and contains 84 distinct subsets and almost 5000 data points.^21^ The top-ranked functional at each rung of Jacob’s ladder, according to this dataset, is shown in Fig. 3.

**FIG. 4.**
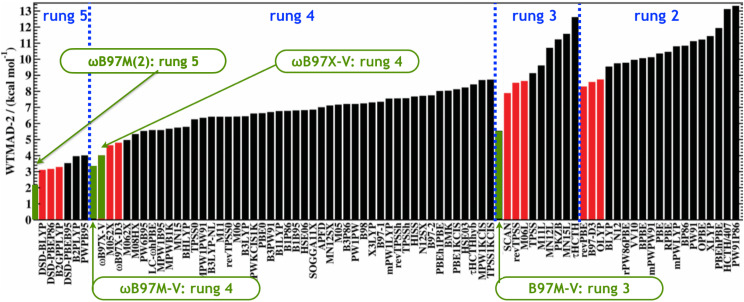
Weighted errors (in kcal/mol) for a range of functionals, assessed using the GMTKN55 dataset and arranged according to the rungs of Jacob’s ladder in Fig. 3. The figure is adapted from Ref. 90 but includes additional data from Refs. 91 and 62. Adapted with permission from Goerigk *et al.*, Phys. Chem. Chem. Phys. **19**, 32184 (2017). Copyright 2017 Published by the PCCP Owner Societies.

The GMTKN55 dataset is another large diverse set of benchmarks for main-group chemistry,^90^ and Fig. 4 summarizes the performance of a large range of functionals for this dataset. Consistent with the Jacob’s ladder taxonomy, the performance of the best functional improves at each rung of the ladder, showing that the inclusion of additional physical content does indeed improve accuracy. While it is often (correctly) stated that DFT results on a given molecule are not systematically improvable by switching from one functional to another, these results illustrate that in a statistical sense, DFT does systematically improve when represented by the best functional at each rung of the ladder. The same need not be true if one considers worse-performing functionals at each level, as the additional flexibility associated with higher rungs on Jacob’s ladder makes it quite possible to overfit complicated functional forms using limited data, especially where meta-GGA functionals are concerned.

Diving a bit deeper into the data shown in Fig. 4 reveals a variety of other interesting observations.

• LSDA (rung 1) is essentially useless for chemical applications. A good GGA such as B97-D3 is the simplest and lowest-cost DFT method that is useful for chemistry.
• A good meta-GGA, as exemplified by B97M-V, offers striking improvements over the best GGA across all categories. It is clear that meta-GGAs can deliver significantly higher accuracy than GGAs.
• Significant further improvement is delivered by the best hybrid functionals, exemplified by *ω*B97X-V as a RSH-GGA and *ω*B97M-V as a corresponding RSH-meta-GGA. This improvement arises primarily from better accuracy for barrier heights, thermochemistry, and isomerization energies. There is good reason for hybrids to be a default choice for chemical modeling.
• The best DH-DFAs offer further improvements in the same categories where hybrids improve over meta-GGAs: barrier heights, thermochemistry, and isomerization energies. However, the significantly higher cost of DH-DFAs means that they are often used only for single-point energy calculations at stationary points optimized at lower levels of theory. Q-Chem includes the efficient occ-RI-K algorithm^92^ to significantly reduce the additional compute cost of DH-DFAs. Some parallel timings are given in Sec. IX.
• The gap in accuracy between DFT and the best wave function theories remains quite substantial. For both bonded and non-bonded interactions, errors associated with coupled-cluster (CC) methods that include triple excitations [CCSD(T) or better] are on the order of 5× smaller than those for the best rung-5 density functionals.^59^ Therefore, despite the much higher computational costs, there remains strong incentive to perform CC calculations when possible. Some of Q-Chem’s CC capabilities are described in Sec. III.

Further details regarding the combinatorial design strategy used to obtain the best functionals at rungs 3, 4, and 5 can be found in the work of Mardirossian and Head-Gordon.^41,52,53,59^ It should be noted that statistical assessments of DFAs are only as transferable as the data they are built upon. The transferability of the conclusions discussed above to similar systems is supported by the fact that broadly similar conclusions can be drawn from other large-scale data assessments, e.g., comparing MGCDB84 vs GMTKN55 for main-group compounds. It is a separate issue to investigate the performance of density functionals for very different classes of molecules, such as transition metal compounds. (These have been the target of several other recent benchmark studies.^93,94^) Similarly, interest in the quality of densities derived from DFT must be separately assessed, either directly^95^ or via properties such as electrical moments.^96–99^ Similar considerations apply to other molecular properties, such as polarizabilities^100^ and nuclear magnetic resonance (NMR) chemical shifts.^101^

### B. Thermally assisted-occupation DFT

Systems with strong static correlation remain very challenging for conventional Kohn–Sham DFT. Q-Chem 5 contains *thermally assisted-occupation* (TAO-)DFT,^102–104^ an efficient means to explore ground-state properties of large electronic systems with strong static correlation. Unlike Fermi smearing^105^ (also supported by Q-Chem), which is a convergence aid for small-gap systems, TAO-DFT aims to access densities beyond those obtainable from a single Kohn–Sham determinant. TAO-DFT is similar to Kohn–Sham DFT in computational complexity but represents the ground-state electron density in terms of orbitals with fractional occupation numbers governed by a Fermi–Dirac distribution at a fictitious temperature that is related to the strength of static correlation. In TAO-DFT, static correlation can be approximately described by the entropy contribution,^102^ even when semilocal^102,103^ or hybrid^104^ density functionals are employed. A self-consistent scheme defining the fictitious temperature has been recently developed for diverse applications.^106^ By combining computational efficiency with reasonable accuracy, TAO-DFT is well positioned to investigate the ground-state properties of electronic systems at the nanoscale, especially those possessing strong static correlation effects.^107–111^ TAO-DFT has recently been combined with *ab initio* molecular dynamics.^112^

### C. Excited-state DFT methods

The TDDFT approach^113,114^ extends ground-state DFT to electronically excited states via the linear response (LR) formalism,^115,116^ incorporating electron correlation at a computational cost equivalent to its uncorrelated Hartree–Fock analog, the configuration-interaction singles (CIS) method.^114^ This relatively low cost makes LR-TDDFT (Sec. II C 1) the most widely used method for computing vertical excitation spectra and for exploring excited-state potential energy surfaces (computational photochemistry, Sec. II C 2). An alternative to the LR formalism is “real-time” TDDFT,^117,118^ also known as *time-dependent Kohn–Sham* (TDKS) theory,^119–121^ which is discussed in Sec. II C 3 and which can be used to compute broadband excitation spectra. Finally, an altogether different category of DFT-based excited-state methods is the ΔSCF formalism, which is a state-specific approach that fully accounts for orbital relaxation in the excited state and can be used to describe challenging problems such as excited-state charge separation and states with double-excitation character, thereby sidestepping known systemic problems with LR-TDDFT while retaining SCF cost. The ΔSCF approach is discussed in Sec. II C 4.

#### 1. LR-TDDFT

Despite its popularity, LR-TDDFT does have systemic problems for certain classes of excited states, the most infamous of which is its dramatic underestimation of excitation energies having charge-transfer (CT) character.^85–87,122–127^ Nevertheless, this method often achieves an impressive statistical accuracy of 0.2–0.3 eV for low-lying valence excitation energies,^128^ giving it a wide domain of applicability despite recognized shortcomings.

The CT problem, in particular, can be largely ameliorated through the use of *long-range corrected* (LRC) functionals,^84–89^ which are RSH functionals in which the fraction of Hartree–Fock exchange is required to go to unity as *r*_12_ → *∞*. The most popular such functional is LRC-*ω*PBE,^87,129^ along with its short-range hybrid cousin, LRC-*ω*PBEh,^126^ although other variants are available, including LRC-*μ*BLYP and LRC-*μ*BOP.^86,88,130^ In addition to these LRC-GGAs, Q-Chem 5 also includes the relatively new revM11 functional,^131^ a LRC-meta-GGA functional specifically optimized for long-range CT excitations.

For best results, the range-separation parameter (*ω* or *μ*) is often “tuned” in order to set the frontier energies based on the molecule’s own (ΔSCF) ionization energy (IE),^89,132–134^

$$\text{IE}\left(\omega \right)=-\epsilon_{\text{HOMO}}\left(\omega \right).$$ (3)

In Q-Chem 5, an alternative “global density-dependent” (GDD) tuning procedure is available.^135–137^ Following a standard SCF calculation with a functional such as LRC-*ω*PBE, the GDD procedure automatically determines a new tuned value (*ω*_GDD_) based on the size of the exchange hole. This approach appears to avoid system-size-dependent problems with the value of *ω* tuned according to Eq. (3).^137^

#### 2. Exploring excited-state potential surfaces

Q-Chem 5 contains new tools that enable the exploration of excited-state potential energy surfaces with LR-TDDFT, including algorithms for locating minimum-energy crossing points (MECPs) along conical seams. For a molecule with *n*_vib_ = 3*n*_atoms_ − 6 vibrational degrees of freedom, the conical seam (or “conical intersection”) is a (*n*_vib_ − 2)-dimensional subspace within which two electronic states are exactly degenerate. Conical intersections serve as photochemical funnels for nonadiabatic dynamics,^138,139^ so locating the MECP (i.e., the lowest-energy point within the degenerate subspace) can help to rationalize excited-state dynamics by providing a single chemical structure to represent the whole seam space.^140^

Orthogonal to the conical seam is the two-dimensional *branching space*, within which any infinitesimal displacement lifts the degeneracy between electronic states |Ψ_*J*_⟩ and |Ψ_*K*_⟩.^138,141^ The branching space is spanned by two (nonorthogonal) vectors,

$$g_{JK}=\frac{\partial E_{J}}{\partial R}-\frac{\partial E_{K}}{\partial R}$$ (4)

and

$$h_{JK}=\left\langle \Psi_{\;J}\left|\frac{\partial \hat{H}}{\partial R}\right|\Psi_{\;K}\right\rangle ,$$ (5)

where **R** indicates the nuclear coordinates. Operationally, the gradient difference (“**g**-vector”) is easily computed using any excited-state method for which analytic gradients are available, but the nonadiabatic coupling (“**h**-vector”) is less routinely available. Analytic **h**-vectors are available in Q-Chem 5 for both CIS and LR-TDDFT,^141–145^ which greatly facilitates efficient optimization of MECPs by means of a projected-gradient algorithm that optimizes directly in the seam space.^146^ Alternatively, for excited-state methods where analytic gradients (and therefore **g**_*JK*_) are available but analytic derivative couplings (**h**_*JK*_) are not, Q-Chem provides a branching-plane updating algorithm to optimize MECPs.^140,147^ This is significantly more efficient^140^ than alternative penalty-function methods,^148^ which can also be used in the absence of **h**_*JK*_. The projected-gradient algorithm is the most efficient approach of all, however, converging in fewer steps while the computation of **h**_*JK*_ adds a modest 10%–20% overhead to the cost of computing the gradients for states *J* and *K*.^142,149,150^ For molecules with intersystem crossing, analytic gradients and derivative couplings at the CIS and LR-TDDFT levels are available within both the spin-diabatic and spin-adiabatic representations.^151,152^

Nonadiabatic trajectory simulations at the LR-TDDFT level are available in Q-Chem and take advantage of these analytic derivative couplings. These simulations can be performed using the Tully’s “fewest switches” surface hopping (FSSH) algorithm^153,154^ or using an “augmented” FSSH algorithm that includes decoherence effects on the electronic amplitudes.^155,156^ These corrections are necessary in order to maintain detailed balance and to describe both short- and long-time relaxation dynamics, including Marcus theory.^157–159^ A Python framework for performing FSSH simulations using Q-Chem is also available.^160^

**FIG. 5.**
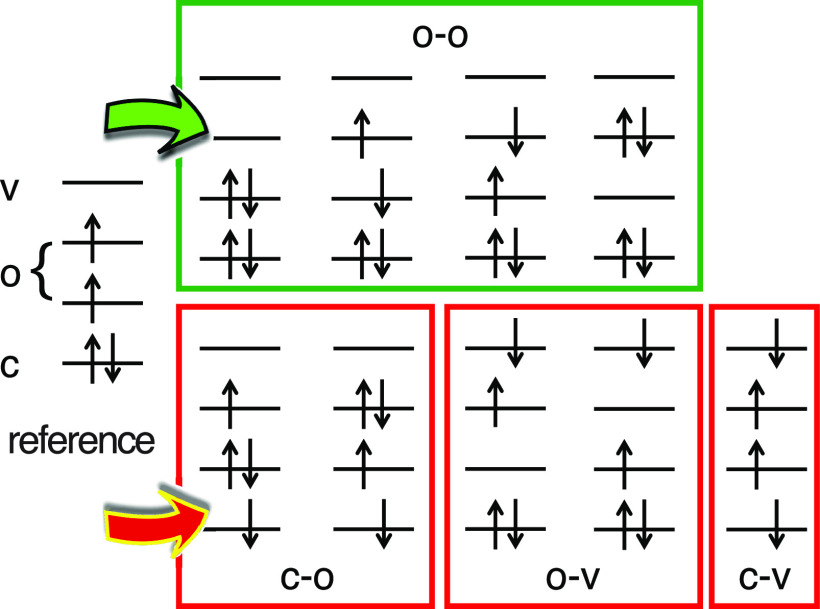
Illustration of the spin–flip TDDFT excitation space for a (4e, 4o) model, starting from a high-spin triplet reference. Proper spin eigenfunctions can be formed from the four determinants in the o–o subspace, but the remaining determinants are missing one or more complementary spin functions. Adapted from X. Zhang and J. M. Herbert, J. Chem. Phys. **143**, 234107 (2015) with the permission of AIP Publishing.

A systematic shortcoming of LR-TDDFT that is relevant here is an incorrect description of the topology around any conical intersection that involves the ground state; in such cases, the branching space predicted by LR-TDDFT is one-dimensional rather than two-dimensional.^141,161^ This problem has its roots in the fact that any excited-state method based on response theory treats the “reference state” (usually the ground state) in a fundamentally different manner as compared to the “response” (excited) states. This can cause difficulties when the reference state becomes quasi-degenerate with the lowest excited state, and in the context of nonadiabatic trajectory simulations, this imbalance can manifest as SCF convergence failure in the vicinity of a conical intersection.^162^ The “spin–flip” approach to LR-TDDFT^163–165^ resolves this problem^141,142^ by using a reference state with a different spin multiplicity as compared to the target states of interest. An example is shown in Fig. 5, which depicts the excitation space for a case where a high-spin triplet reference state is used to generate determinants for singlet states, including the closed-shell S_0_ ground state. The spin–flip single-excitation manifold contains a subset of the possible determinants that are doubly excited with respect to S_0_, including the one (in the “o–o” subspace in Fig. 5) that is necessary to provide proper topology at the S_0_/S_1_ conical intersection.^142,161^ In Q-Chem 5, nonadiabatic coupling vectors **h**_*JK*_ are available for both conventional and spin–flip variants of LR-TDDFT.^142^

**FIG. 6.**
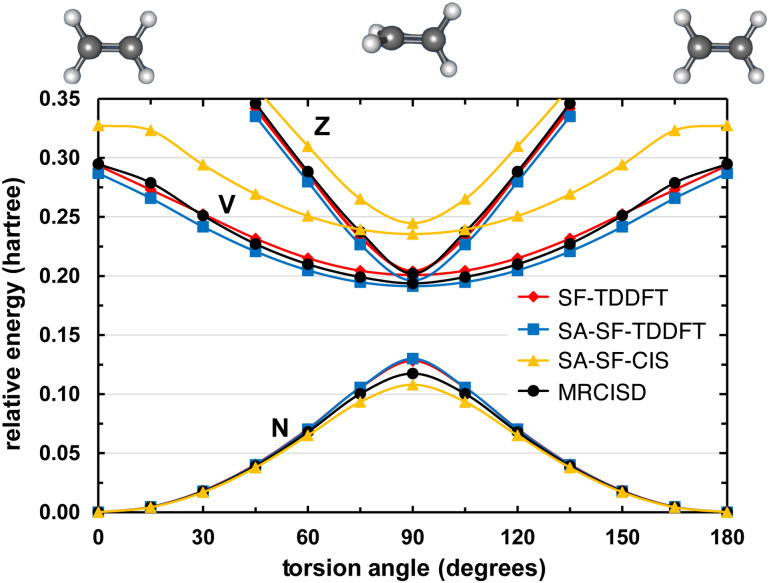
Potential energy curves for the singlet N [${\left(\pi \right)}^{2}{\left(\pi^{*}\right)}^{0}$], V [${\left(\pi \right)}^{1}{\left(\pi^{*}\right)}^{1}$], and Z [${\left(\pi \right)}^{0}{\left(\pi^{*}\right)}^{2}$] states of C_2_H_4_, twisting along the C–C axis, computed using various spin–flip methods in comparison to multireference benchmarks. Both SA-SF-TDDFT and SA-SF-CIS correctly describe the topology around a conical interaction, but the latter lacks dynamical correlation and therefore excitation energies are not accurate. Adapted from X. Zhang and J. M. Herbert, J. Chem. Phys. **143**, 234107 (2015) with the permission of AIP Publishing.

While the spin–flip approach rigorously cures the topology problem at conical intersections,^141,142^ it unfortunately exacerbates problems with spin contamination. This is especially true as one moves away from the Franck–Condon region and starts to break bonds, for which singlet and triplet states often become comparable in energy, and may necessitate the use of state-tracking algorithms to ensure that a geometry optimization or dynamics trajectory remains on a potential surface of consistent spin multiplicity.^166–169^ At the heart of this problem is the fact that each of the determinants in the c-o, o-v, and c-v subspaces in Fig. 5 is missing one or more of the complementary determinants^170–172^ needed to form an ${\hat{S}}^{2}$ eigenstate. The missing determinants are absent because they cannot be generated from the reference state via a single excitation combined with a single *α* → *β* spin flip. However, these determinants *can* be generated, in an automated manner that does not increase the formal computational scaling of LR-TDDFT, by means of a tensor equation-of-motion (EOM) formalism.^169,173–175^ This formalism has been used to develop a “spin-adapted spin–flip” (SA-SF) TDDFT method,^169^ which preserves proper topology at conical intersections but also restores spin multiplicity as a good quantum number. Figure 6 shows that SA-SF--TDDFT results are close to multireference benchmarks for the challenging problem of twisting ethylene by 90° about its C–C axis. Analytic gradients for SA-SF-TDDFT are not yet available, but this method can be used to check the veracity of any heavily spin-contaminated results that are obtained with other flavors of LR-TDDFT.

SF-TDDFT methods are also suitable for treating other types of electronic structure that are not accessible by the standard Kohn–Sham DFT, such as polyradicals and single-molecule magnets.^163,164,176,177^

#### 3. “Real-time” TDDFT

The term “TDDFT” is used almost universally to refer specifically to LR-TDDFT, which despite its name is a strictly frequency-domain theory with no explicit time dependence, at least not within the ubiquitous adiabatic approximation that is used in all practical implementations.^114,115^ However, just as the ground-state Kohn–Sham problem is based on a one-electron analog of the time-independent Schrödinger equation [Eq. (2)], at the foundation of TDDFT is a one-electron analog of the time-*dependent* Schrödinger equation, which governs the time evolution of |Φ_*s*_⟩ and thus the Kohn–Sham MOs. The latter evolve in time according to

$$\text{i}\hbar \;\frac{d\phi_{k}\left(r,t\right)}{dt}=\hat{F}\phi_{k}\left(r,t\right).$$ (6)

Using this TDKS equation, the MOs can be propagated in time following a perturbation of the ground state density at *t* = 0 that generates a (non-stationary) superposition of excited states. Information about electronic excitation energies is encoded into the time evolution of this superposition state, and an entire broadband excitation spectrum can be obtained via Fourier transform of the time-dependent dipole moment function, with a spectral resolution that improves upon further time propagation.^117,178^ This approach has been given the unwieldy moniker of “real-time” TDDFT,^117,118^ although calling it TDKS theory avoids confusion with the more widespread LR-TDDFT approach.^119–121^

In the limit of a weak perturbation at *t* = 0, propagated to *t* → *∞* to obtain narrow spectral lines, TDKS spectra are equivalent to those obtained using LR-TDDFT,^178^ but the TDKS approach need not be limited to the weak-field LR regime and can be used to explore strong-field dynamics,^179^ strong-field ionization,^180–183^ and high-harmonic spectra,^120,184–187^ for example. [Ionization requires the use of complex absorbing potentials (CAPs), which are discussed in Sec. V B. These are available for use in TDKS simulations,^120,121^ along the lines of the atom-centered potentials described in Refs. 180–183.] In this way, TDKS simulations can describe time-dependent electron dynamics beyond the Born–Oppenheimer approximation, where the electrons are out of equilibrium with the nuclei. At present, Q-Chem’s implementation of the TDKS method^120,121^ is limited to clamped-nuclei simulations, meaning electron dynamics only.

Time propagation according to Eq. (6) is complicated by the fact that $\hat{F}$ depends on the MOs and thus the effective Hamiltonian is time-dependent. The most widely used propagation algorithm is the modified-midpoint method,^188^ for which the cost of one time step is the same as the cost of one SCF cycle of a ground-state calculation. (It should be noted that for *electron* dynamics, the fundamental timescale is attoseconds, and therefore, time steps Δ*t* ∼ 0.04 a.u. = 10^−18^ s are typical.^119^) Q-Chem’s implementation of the TDKS approach also contains several predictor/corrector algorithms as alternatives to the modified-midpoint approach.^119^ These are stable over longer time steps Δ*t* and furthermore facilitate on-the-fly detection of instabilities that can lead to spurious peak-shifting but are not always evident simply by monitoring energy conservation, which is a necessary but not a sufficient condition for accurate integration of Eq. (6).^119^

**FIG. 7.**
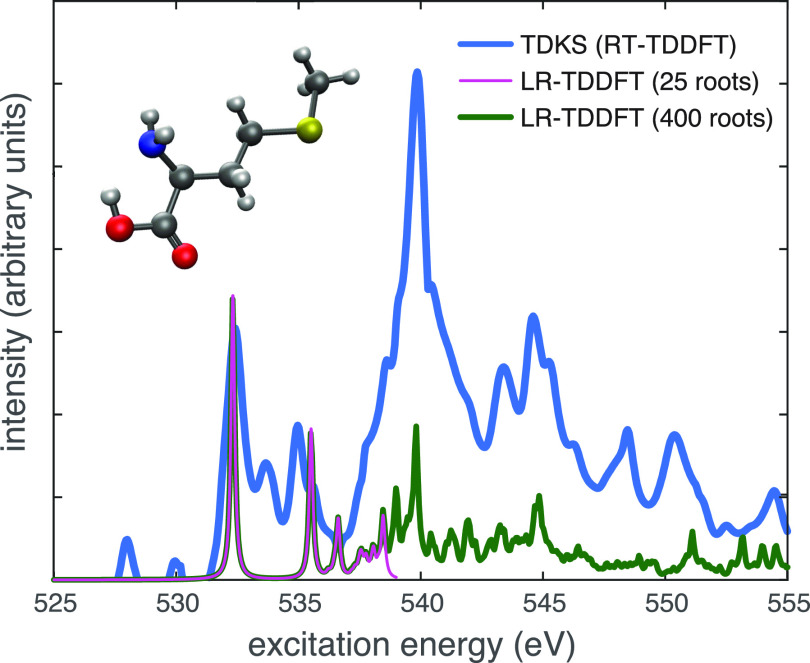
Absorption spectra of methionine at the oxygen K-edge computed at the level of SRC1-R1^189^/def2-TZVPD.^75,76^ A broadband TDKS calculation is shown along with two LR-TDDFT spectra using different numbers of roots. The former is obtained from 7.3 fs of time propagation with Δ*t* = 0.02 a.u. The LR-TDDFT calculations use an active space consisting of all virtual MOs but only the O(1s) orbitals from the occupied space. Features below 531 eV in the TDKS spectrum correspond to N(1s) → continuum transitions that are excluded by this active-space approximation. Data are taken from Ref. 121.

Figure 7 illustrates a TDKS calculation of a broadband excitation spectrum, corresponding to x-ray absorption (XAS) at the oxygen K-edge above 530 eV.^120,121^ This spectrum was obtained from 7.3 fs of time propagation with Δ*t* = 0.02 a.u. (meaning 15 140 time steps) using Padé approximants to accelerate convergence of the Fourier transform.^120,121,190^ Also shown are two LR-TDDFT excitation spectra computed using the same functional and basis set, which reproduce the same basic features; however, hundreds of excited states are required in order to get beyond the near-edge peak, corresponding to the O(1s) → LUMO transition. In the TDKS approach, the carbon or nitrogen K-edge spectra (at lower excitation energies) are obtained from the same calculation, although the sulfur K-edge appears at significantly higher energy (above 2400 eV) and requires a smaller time step. In contrast, LR-TDDFT excitation spectra must be computed in terms of individual eigenstates; frozen occupied orbitals are required in order to make core-level excitations emerge as the lowest-energy states, and even so, hundreds of eigenstates are required to converge the features of the spectrum. For the LR-TDDFT calculations in Fig. 7, only the two O(1s) orbitals of the methionine molecule were active from the occupied space. Despite this restriction, several hundred states are required in order to access excitation energies above the first near-edge features, and this quickly becomes prohibitive for large molecules, especially in terms of memory. These requirements for the LR-TDDFT calculation can be reduced by judicious use of frozen orbitals,^191,192^ and much larger examples (e.g., C_70_) have been reported using Q-Chem’s LR-TDDFT code.^191^ However, the memory requirement for TDKS (without approximation) is a mere 2× the memory for a ground-state SCF calculation, which is quite minimal. That said, whereas LR-TDDFT naturally provides CIS-like excitation amplitudes that characterize each excited state, from TDKS calculations it is more difficult to extract information regarding the specific MOs that contribute to various spectral features, although some ideas to this end have been put forward.^190,193^

Some of these same considerations apply when many-body methods are used to compute x-ray spectra, as described in Sec. V A. The LR-TDDFT approach to core-level spectroscopy is discussed alongside these approaches in that section.

#### 4. ΔSCF and ROKS methods

LR-TDDFT tends to fail systematically for excited states that involve a significant change in the density, including the aforementioned CT excitations, but also states with double-excitation character,^194^ which are often either missing entirely from the LR-TDDFT excitation spectrum or else are badly in error. Both types of states are characterized by significant orbital relaxation. Indeed, it has recently been argued that much of what passes for double-excitation character (e.g., in the well-known case of the 2^1^A_*g*_ state of butadiene) is simply orbital relaxation and that double excitations are required within a single-reference CI formalism simply because the optimal excited-state MOs are very different from those optimized for the ground state.^195^ In such cases, it may make sense to optimize the MOs for the excited state directly. This is the basis for the “ΔSCF” approach to excitation energies, in which one uses an orbital-relaxed, non-*aufbau* Slater determinant as an approximation for the excited-state wave function. In general, these non-*aufbau* solutions are saddle points (rather than local minima) in the space of MO coefficients, and orbital optimization runs the risk of variational collapse to the ground-state solution.

A popular means to overcome this limitation is the *maximum overlap method* (MOM) of Gill and co-workers,^196–198^ which has been improved in Q-Chem 5 by the addition of an “initial MOM” (IMOM) variant.^198^ Starting from a user-specified non-*aufbau* electron configuration (using MOs determined from a previous calculation), the MOM and IMOM algorithms attempt to preserve the character of this state at each step of the SCF orbital optimization procedure. While the IMOM algorithm tends to be more robust as compared to the original MOM, neither one is guaranteed to avoid variational collapse. Q-Chem 5 offers two new algorithms that are much more reliable in this capacity: squared-gradient minimization (SGM)^199^ and state-targeted energy projection (STEP).^200^

The SGM algorithm converts the unstable saddle-point search associated with excited-state orbital optimization into a simpler minimization problem by considering the squared-gradient $\|\partial L/\partial \theta {\|}^{2}$ of an excited-state Lagrangian L(θ), where ***θ*** is a vector of orbital-rotation variables. SGM is far more robust than either MOM or IMOM, although it is a few times more expensive (per iteration) as compared to the ground-state SCF technology that underlies MOM,^199^ and furthermore, not every local minimum of $\|\partial L/\partial \theta {\|}^{2}$ corresponds to a physically meaningful state.^200^ An alternative is the STEP algorithm, which has the same cost as MOM but tends to be more robust.^200^ This approach uses a level-shift in order to optimize a determinant containing a “hole” in the occupied space, using nothing more than the ground-state machinery of iterative Fock-matrix diagonalizations.

Both the SGM and STEP algorithms succeed in a variety of cases where MOM and IMOM suffer variational collapse.^199,200^ For a challenging database of doubly excited states,^201^ ΔSCF excitation energies computed with the B97M-V functional are only 0.15 eV away from theoretical best estimates, with a maximum error <0.5 eV.^199,200^ (Errors for the same dataset at the CC3 level are ∼1 eV,^201^ despite the inclusion of triple excitations.) The ΔSCF approach can also be used for ionization energies, to access the full valence photoelectron spectrum by systematically removing an electron from orbitals below the HOMO.^200^ Because the ΔSCF approach is based on ground-state machinery, analytic nuclear gradients and even analytic Hessians are available for many different density functionals. Geometry optimization can be performed in the presence of a valence hole in order to compute the adiabatic ionization energy for ionization below the HOMO.^200^

**FIG. 8.**
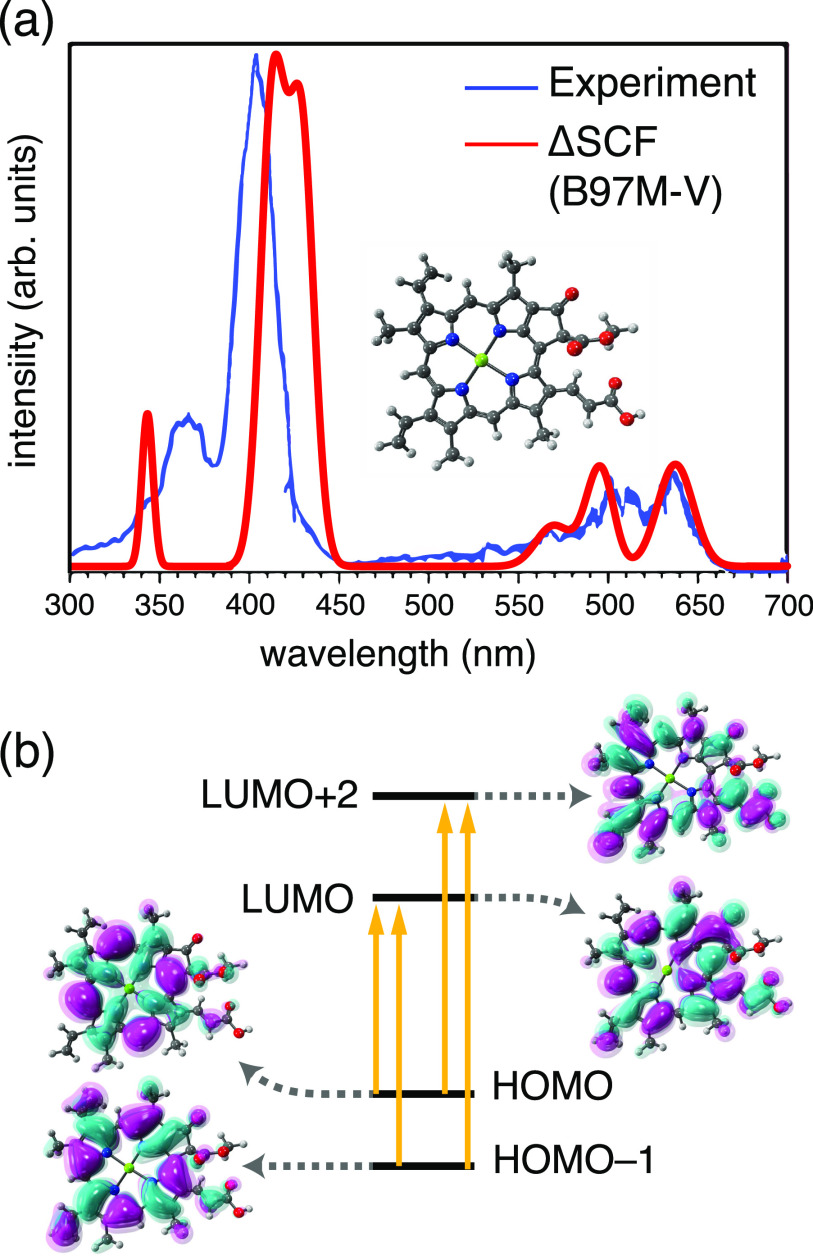
(a) Absorption spectra of the Mg-chlorin chromophore of Chl *a* (structure shown), comparing a gas-phase experimental spectrum^202^ to a ΔSCF calculation at the B97M-V/def2-TZVPD level, which is then spin-purified using Eq. (7).^200^ (b) Four-orbital model demonstrating the states that were targeted using the STEP algorithm and included in the excitation spectrum shown in (a). Adapted with permission from K. Carter-Fenk and J. M. Herbert, J. Chem. Theory Comput. **16**, 5067 (2020). Copyright 2020 American Chemical Society.

As a showcase of the ΔSCF approach, Fig. 8(a) presents a computed absorption spectrum for the chlorin moiety of chlorophyll *a*.^200^ In accordance with Gouterman’s four-orbital model,^203^ the ΔSCF calculation includes the four excitations that are shown in Fig. 8(b), and the result is in semiquantitative agreement with a recent gas-phase experimental spectrum.^202^ It is worth noting that the ΔSCF approach uses a single Slater determinant to describe the excited-state wave function, but for an open-shell singlet, a minimum of two determinants is required in order to obtain a spin eigenstate. It is therefore not unusual for the ΔSCF wave functions to exhibit $\left\langle {\hat{S}}^{2}\right\rangle \approx 1$ (in units of *ℏ*^2^), indicating approximately equal mixture of singlet and triplet. A simple spin-purification procedure,^204,205^

$$E_{\text{singlet}}\approx 2E_{\text{mixed}}-E_{\text{triplet}},$$ (7)

can be used as an *a posteriori* correction that requires only the triplet energy (*E*_triplet_) in addition to the spin-contaminated energy *E*_mixed_.

A more elaborate method is to optimize the orbitals directly using Eq. (7) as the total energy expression, which forms the basis of the *restricted open-shell Kohn–Sham* (ROKS) formalism.^206,207^ ROKS has been found to be effective in predicting energies of excited states of small molecules,^207^ as well as charge-separated excited states of organic light emitting diode materials,^208^ to an accuracy of ∼0.2–0.3 eV. In conjunction with the SGM algorithm, the ROKS approach can be used to predict core-level excitation energies to an accuracy of 0.2–0.3 eV,^209^ as described in Sec. V A. Nuclear gradients for ROKS are available in Q-Chem,^207^ permitting geometry optimizations and (finite-difference) frequency calculations in the excited state. Finally, note that Eq. (7) is only appropriate in the case of two unpaired electrons, and more elaborate treatments are necessary in more complicated cases.^210–212^

## III. MANY-BODY METHODS

Whereas Jacob’s ladder of DFT provides a hierarchy of methods that are improvable only in a statistical sense, meaning that the best functionals on a given rung are *usually* (but not always) better than the ones on the rung below, many-body approaches to the electron correlation problem provide a systematic and rigorous way to approach the exact solution for any given molecule.^213^ Particularly powerful are the hierarchical approximations built upon the Møller–Plesset (MP) perturbation theory and coupled-cluster (CC) frameworks,^214^ which do not involve system-specific parameterization. Q-Chem offers fast and efficient implementations of the standard many-body approaches, including MP2, MP3, CCSD, and CCSD(T). These codes exploit shared-memory parallelism (OpenMP) as well as numerous cost-reduction and resource-reduction techniques. Among these are resolution-of-identity approximations (also known as density fitting),^215^ Cholesky decomposition of the electron repulsion integrals (ERIs),^215,216^ frozen natural orbitals,^217,218^ and efficient tensor libraries.^12,13^ Mixed-precision CC and EOM-CC calculations are also available for energies, properties, and gradients.^219^ Q-Chem 5 also features mixed precision (T) calculation. A combination of these techniques enabled calculations of magnetic properties of single-molecule magnets and even infinite spin-chains at the CC/EOM-CC level of theory.^177,220–223^ A new object-oriented implementation of the MP2 energy and gradient and of MP3 energies (including orbital-optimized variants) requires no storage of amplitudes or four-index electron repulsion integrals and is optimized for OpenMP parallelism.

Single-reference wave function methods can be extended to tackle many problems traditionally described as “multi-reference.” For example, many types of open-shell and electronically excited species can be handled by equation-of-motion (EOM)-CC methods^224–226^ as well as by methods based on the algebraic diagrammatic construction (ADC).^227^ At the same time, Q-Chem also contains methods based on the CI formalism, including active-space methods for the treatment of strong correlation. Those methods are described in Sec. IV, whereas the present section highlights some examples of new development in MP*n* and CC methods.

### A. Extensions of MP*n* theory

MP*n* theory is traditionally applied to the Hartree–Fock determinant, on the assumption that it is the best single-determinant approximation to the correlated wave function, an assumption that may not be valid for open-shell systems or cases where static correlation is important. Deficiencies of Hartree–Fock orbitals include excessive spin polarization (i.e., artificial symmetry breaking)^228^ and charge distributions that are slightly too diffuse and too polar.^229^ These deficiencies can be addressed using orbital-optimized (OO) approaches in which the orbitals are determined by minimizing a correlated energy expression. In the context of MP2, this can be done using either the opposite-spin correlation energy^230^ or the total MP2 correlation energy.^231,232^ However, OOMP2 exaggerates correlation effects and this can lead to artifacts, especially when orbital energy gaps become small.^233^ This issue is addressed by an improved version of OOMP2, termed *κ*-OOMP2,^234^ which applies a novel energy-dependent regularization to the electron repulsion integrals,

$$〈ij\|ab〉\left(\kappa \right)=〈ij\|ab〉\left[1-\exp\left(-\kappa \Delta_{ij}^{ab}\right)\right].$$ (8)

This removes divergences associated with small denominators $\Delta_{ij}^{ab}=\epsilon_{a}+\epsilon_{b}-\epsilon_{i}-\epsilon_{j}$ in the *κ*-OOMP2 energy expression

$$E=E_{0}-\underset{i<j}{\sum }\underset{a<b}{\sum }\frac{{\left[\left\langle ij\|ab\right\rangle \left(\kappa \right)\right]}^{2}}{\Delta_{ij}^{ab}}.$$ (9)

With the recommended choice of *κ* = 1.45 a.u., *κ*-OOMP2 significantly improves upon standard MP2 for thermochemical properties, non-covalent interactions, and reaction barrier heights.

The use of *κ*-OOMP2 orbitals also sidesteps artificial symmetry breaking, and in this capacity the method can be useful for diagnosing the presence of strong correlation. By design, *κ*-OOMP2 includes a simple treatment of dynamical (or weak) correlation but zero contribution in the strongly correlated limit.^235^ In molecules without strong correlation, spin symmetry-breaking (SSB) exhibited by Hartree–Fock orbitals is dramatically reduced by *κ*-OOMP2, signifying that the SSB in question was “artificial,” caused by the absence of dynamic correlation. In molecules *with* strong correlation, Hartree–Fock SSB is preserved in the *κ*-OOMP2 orbitals, signifying the presence of essential SSB associated with multireference character.

This approach helped to resolve a controversy^236,237^ regarding the character of electron correlations in fullerenes. Hartree–Fock theory shows dramatic SSB in C_60_, with the global-minimum solution exhibiting complex and general symmetry breaking, which has been interpreted as a signature of strong correlation and polyradical character. However, the *κ*-OOMP2 global-minimum orbitals remove this artificial SSB and are spin-pure, thus establishing that C_60_ is not a strongly correlated system, which is consistent with other observables.^235^ By contrast, more reactive fullerenes, such as C_30_, do exhibit essential SSB in *κ*-OOMP2. In conjunction with other observables, this confirms the presence of strong correlations in their ground states. By using *κ*-OOMP2 with either spin projection or complex orbitals, one can treat large diradicaloid systems, on the size scale of the reactive fullerenes.^238^

**FIG. 9.**
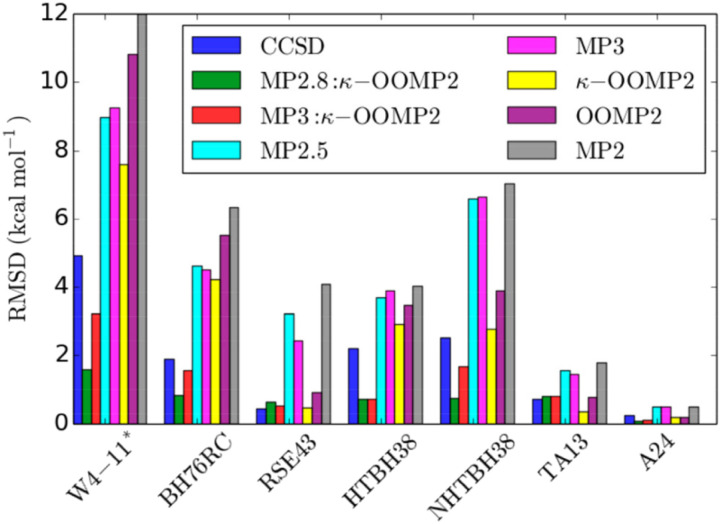
RMS errors (in kcal/mol) relative to benchmark CCSD(T) values for seven different datasets assessed using MP2, MP3, and CCSD methods. Reprinted with permission from Bertels *et al.*, J. Phys. Chem. Lett. **10**, 4170 (2019). Copyright 2019 American Chemical Society.

The *κ*-OOMP2 energy and gradient are implemented in Q-Chem 5 within a modern MP*n* suite that includes MP3. The long-neglected MP3 *ansatz*, when used with orbitals from either *κ*-OOMP2 or a good DFA, can deliver accuracy comparable to that of CCSD but is 20–30× faster.^239,240^ Figure 9 illustrates the improvement of *κ*-OOMP2 relative to MP2, as well as the dramatic improvement in MP3 when using *κ*-OOMP2 orbitals instead of Hartree–Fock orbitals.

### B. CC/EOM-CC and ADC methods for open-shell and electronically excited species

Q-Chem contains an ever-growing suite of many-body methods for describing open-shell molecules and excited states.^172^ The EOM-CC^224–226^ and ADC^227,241^ formalisms are two powerful approaches for describing multiconfigurational wave functions within a black-box single-reference formalism. Target states |Ψ_ex_⟩ are described as excitations from a reference state |Ψ_0_⟩,

$$|\Psi_{\text{ex}}〉=\hat{R}|\Psi_{0}〉,$$ (10)

where $\hat{R}$ is an excitation operator parameterized via amplitudes that are determined by solving an eigenvalue problem. In EOM-CC, these amplitudes are eigenvectors of the effective Hamiltonian

$$\bar{H}=e^{-\hat{T}}\hat{H}e^{\hat{T}}\;,$$ (11)

in which $\hat{T}$ is either the CC or the MP2 operator for the reference state. Currently, EOM-CCSD and EOM-MP2 models are available. In ADC, an effective shifted Hamiltonian is constructed using perturbation theory and the intermediate state representation (ISR) formalism,^227,241^ similar to Eq. (10), to afford

$$M=\left\langle \Psi_{\text{ex}}|\hat{H}-E_{0}|\Psi_{\text{ex}}\right\rangle ,$$ (12)

where *E*_0_ is the energy of the MP*n* reference state. Diagonalization of the Hermitian matrix **M** yields excitation energies, and the ADC eigenvectors give access to the excited-state wave function. Second-order standard ADC(2), extended ADC(2)-x, and ADC(3) are available.^241^ For the second-order ADC schemes, spin-opposite-scaled (SOS) variants are also implemented.^242^

**FIG. 10.**
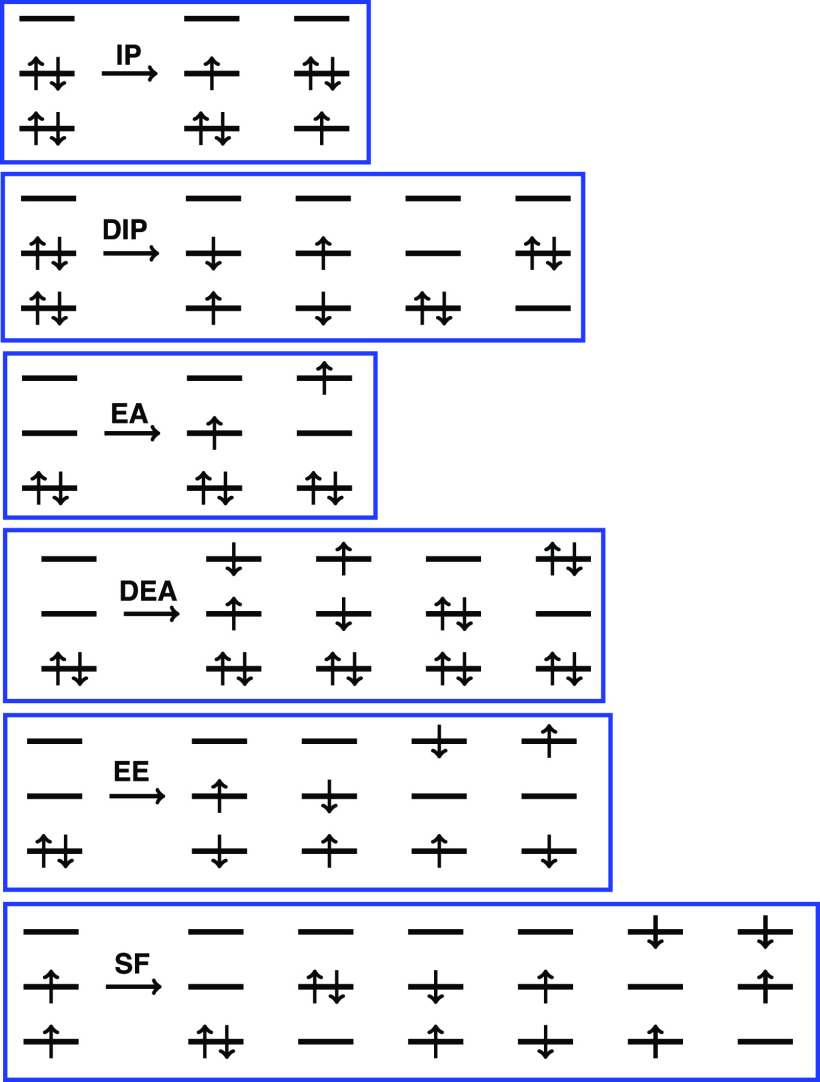
Schematic representation of the manifolds of target states that are accessed within various EOM-CC and ADC formalisms by combining particular choices of reference state and excitation operator in Eq. (10). For example, in the EE models for electronically excited states, the reference |Ψ_0_⟩ is the closed-shell ground-state wave function and the operator $\hat{R}$ conserves the number of *α* and *β* electrons in generating a target manifold of correlated excited-state basis functions. Non-particle-conserving operators (IP, EA, DIP, and DEA) and spin-flipping (SF) operators open a route to the multi-configurational wave functions encountered in radicals, diradicals, triradicals, and bond-breaking processes. Reprinted with permission from D. Casanova and A. I. Krylov, Phys. Chem. Chem. Phys. **22**, 4326 (2020). Copyright 2020 Published by the PCCP Owner Societies.

Various EOM-CC and ADC models are defined by the choice of reference state |Ψ_0_⟩ and excitation operator $\hat{R}$, as illustrated in Fig. 10. The following models are available:^224,227,241^ EE (excitation energies), IP (ionization potentials), EA (electron affinities), SF (spin–flip, for triplet and quartet references), 2SF (double SF, for quintet references); DIP (double IP), and DEA (double EA). At present, the 2SF, DIP, and DEA variants are only available in combination with an EOM treatment.^243^

Analytic gradients^244,245^ and properties^246–248^ are available for most of these models, including transition properties between different target states (e.g., transition dipoles, angular momentum, and electronic circular dichroism rotatory strengths),^249^ nonadiabatic couplings,^250^ spin–orbit couplings,^220,251,252^ and nonlinear optical properties, including two-photon transition moments and (hyper)polarizabilities for both ground and excited states.^253–256^ Extensions of these theories to metastable states^257^ (resonances) and to core-level excitations^258–260^ are also available and are highlighted in Sec. V.

The IP and EA variants of these models afford spin-pure descriptions of ground and excited doublet states and are useful for modeling charge-transfer processes. EOM-SF and SF-ADC methods are suitable for treating diradicals, triradicals, and conical intersections. The DEA and DIP *ansätze* further expand the scope of applicability.^243^ Spin–flip methods can be used to treat strongly correlated systems within an effective Hamiltonian formalism,^221,261,262^ with applications to single-molecule magnets and even infinite spin chains.^222^

**FIG. 11.**
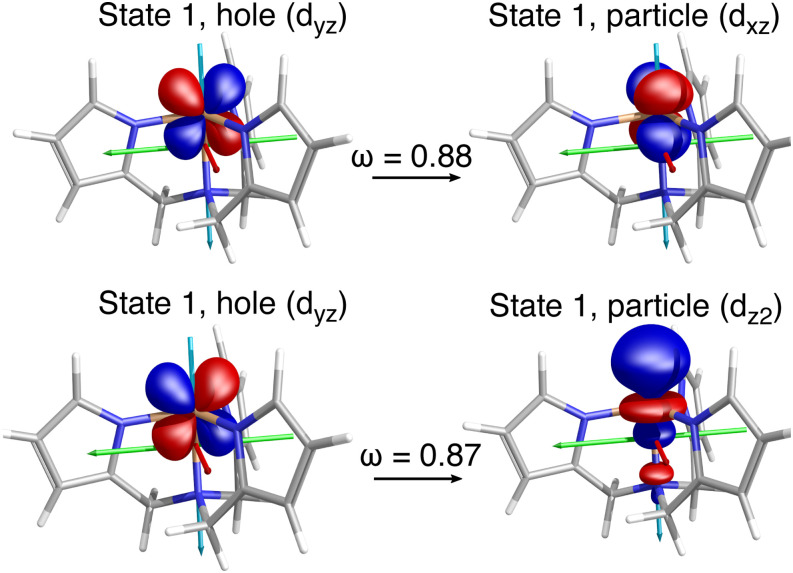
Spinless NTOs for selected transitions between two quintet *d*^6^ states in a tris(pyrrolylmethyl)amine Fe(II) single-molecule magnet,^263^ which are responsible for its large (158 cm^−1^) spin-reversal barrier. Q-Chem’s efficient EOM-CC implementation using the spin–orbit mean-field approximation and the Wigner–Eckart theorem enables calculations for medium-size molecules such as the one shown here. The computed spin-reversal barrier is within 1 cm^−1^ of the experimental value.^252^ The key object, the spinless triplet transition density matrix, provides valuable information about the nature of spin–orbit coupling and the related properties. Spinless NTOs (shown here) allow one to quantify and validate El-Sayed’s rules.^252^ Reprinted with permission from Pokhilko *et al.*, J. Phys. Chem. Lett. **10**, 4857 (2019). Copyright 2019 American Chemical Society.

For visualization purposes, both Dyson orbitals^264^ and natural transition orbitals^265^ (NTOs) are available,^15,88,220,266–269^ including NTOs of the response density matrices for analyzing two-photon absorption^270^ and resonant inelastic x-ray scattering.^271^ Figure 11 highlights the application of these tools to model magnetic properties and spin-forbidden chemistry. Exciton analyses,^267,268,272–274^ bridging the gap between the quasiparticle and MO pictures of excited states, enable the calculation and visualization of electron–hole correlation.^89,267,268,272,273^

## IV. ACTIVE-SPACE METHODS FOR STRONG CORRELATION

The applicability of single-reference methods rests on an assumption that the wave function is dominated by a single Slater determinant. While justified for ground states of well-behaved closed-shell molecules, this assumption is inappropriate for systems exhibiting strong (or static) correlation, where many Slater determinants may make comparable contributions. Examples of multiconfigurational systems include organic polyradicals and transition metals.^275,276^ While certain classes of multiconfigurational wave functions can be effectively described by single-reference methods, such as EOM-CC and ADC (Sec. III B), more general treatments are sometimes desirable.

The exact solution to the finite-basis Born–Oppenheimer electronic structure problem is the full configuration interaction (FCI) wave function, but factorial scaling generally limits its applicability to very small systems. It is thus more effective to solve the FCI problem within an active space of chemically relevant orbitals that contains the strong correlations, leaving the other orbitals to be described via mean-field theory. Although the introduction of an active space imparts an arbitrariness, which is undesirable for a theoretical model chemistry,^79^ the necessity of active-space methods cannot be denied, despite the need to carefully validate the active-space selection for each particular system and process.

This complete active-space (CAS-)CI *ansatz* can be used on its own^277^ but is more commonly combined with orbital optimization, which defines the popular CASSCF method,^278,279^ also known as the fully optimized reaction space (FORS).^280^ Both CASCI and CASSCF are available in Q-Chem 5, including analytic nuclear gradients.

The CASCI problem still exhibits factorial scaling with respect to the size of the active space. The total number of Slater determinants in an active space with *M* spatial orbitals is

Ndet=MNαMNβ, (13)

where *N*_*α*_ and *N*_*β*_ are the number of *α*- and *β*-spin electrons. This equates to *N*_det_ ∼ 5 × 10^11^ for *M* = 22 and *N*_*α*_ = *N*_*β*_ = 11, which is close to the practical upper limit and is only feasible within a massively parallel framework.^281^ With more typical resources, the limit is *M* ≤ 18. On the other hand, the overwhelming majority of these determinants make only a miniscule contribution to the energy.^282,283^ This enables the development of approximate active-space methods that attempt to identify the most important determinants in an automated way, without solving the full CASCI problem, and are thus extensible to much larger active spaces than conventional CASCI or CASSCF methods. The ability to deploy large active spaces helps to reduce the dependence on the active-space choice and affords more robust performance, including a more balanced treatment of dynamic and non-dynamic correlation. Two such methods, adaptive CI and incremental FCI, are described in this section.

The CASCI method can be extended by adding electronic excitations beyond the active space, as in the restricted active space CI (RAS-CI) approach, with single excitations into (hole) and out of (particle) the active space.^284^ This method has been implemented in Q-Chem using an integral-driven algorithm with exact integrals^285^ and also using the RI approximation.^286^ Similar to EOM-CC and ADC methods, target RAS-CI wave functions can be constructed with a general excitation-type operator (EE, nIP, nEA or nSF; see Fig. 10). The intrinsic lack of dynamic correlation within the RAS-CI family can be addressed by means of multi-reference perturbation theory [RAS-CI(2)]^287^ or by the use of short-range density functional correlation energy (RAS–CI–srDFT).^288,289^ Q-Chem's RAS-CI implementation can compute state and transition properties, including transition dipole moments and spin–orbit couplings.^290^

### A. CI with adaptive selection

“Selected” CI (SCI) methods aim to exploit the sparsity of the Hilbert space by identifying important determinants and diagonalizing the Hamiltonian only within the space of important configurations. Although formulated long ago,^291–296^ these methods have re-emerged recently due to breakthroughs in efficient search of the determinantal space.^297–304^ Q-Chem 5 contains an implementation of the *adaptive sampling configuration interaction* (ASCI) method,^304–306^ which efficiently selects important configurations to yield compact CI wavefunctions that account for most of the correlation energy. Based on the computer resources available, the user selects a maximum number of determinants *t* to keep in the variational CI wave function and a cutoff of the top *c* determinants in this list to generate new determinants that are iteratively considered to replace the least significant members of the *t*-list. While still exponential-scaling, the ASCI algorithm permits dramatically larger FCI calculations than the standard approach. To correct for missing configurations, ASCI can be complemented with a second-order perturbation theory correction for the missing configurations to approach chemical accuracy of ∼1 kcal/mol.

While the “soft exponential” scaling of ASCI is a tremendous improvement over conventional FCI, it is still critically important to minimize the size of the FCI problem if the ASCI algorithm is to obtain chemical accuracy. ASCI can be used as an approximate CASCI solver for CASSCF calculations, with the resulting ASCI-SCF method extends the applicability of CASSCF to problems as large as CAS(50, 50) so that periacenes or iron porphyrin can be handled in this way.^307^ The difference between this and the conventional “hard exponential” limit of around CAS(18, 18) illustrates the utility of the ASCI-SCF method for extending the scale of feasible chemical applications. ASCI-SCF nuclear gradients for geometry optimizations are also available in Q-Chem 5.

### B. Incremental full CI

The method of increments^308–310^ provides an alternative means to approach the FCI solution without the associated exponential scaling via an incremental expansion of correlation energy,^311^

$$E_{\text{c}}=\underset{p}{\sum }\varepsilon_{p}+\underset{p<q}{\sum }\Delta \varepsilon_{pq}+\underset{p<q<r}{\sum }\Delta \varepsilon_{pqr}+\cdots \;.$$ (14)

Q-Chem 5 contains an *incremental FCI* (iFCI) method based on this idea,^312–317^ using occupied MOs for the indices *p*, *q*, *r*, …. Successive *n*-body contributions to Eq. (14) can be computed in a manner that is highly parallelizable, and iFCI recovers both static and dynamic correlation with polynomial scaling. Both the cost and the fraction of *E*_c_ that is recovered depend upon the level of truncation in Eq. (14); tests have shown that a three-body expansion (through *ɛ*_*ijk*_) recovers most of the correlation energy, but a four-body expansion is needed to reproduce full CI to within ∼10^−3^ *E*_*h*_. Equally important to systematic convergence is the use of a localized orbital basis, which greatly speeds up the recovery of dynamic correlation. The generalized valence bond perfect-pairing (GVB-PP) method in Q-Chem^318^ suits this purpose well, providing localized bonding/antibonding pairs of orbitals for iFCI.^314^ When applied to butadiene and benzene, which are two standard test cases for FCI-level approaches,^319^ the four-body iFCI method provides total energies that are within 10^−3^ *E*_*h*_ of other benchmarks.^314,317^

**FIG. 12.**
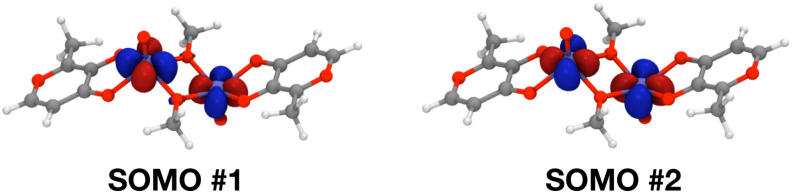
A challenging case of strong and weak correlation: the [(*μ*OCH_3_)VO(ma)]_2_ dimer complex and its two singly occupied MOs. The three-body iFCI yields a singlet–triplet gap within 30 cm^−1^ of experiment.^317^

The iFCI method has also provided solutions equivalent to the largest CI problems to date, including a recent study of transition metal complexes.^317^ For example, the vanadium maltolato dimer, [(*μ*OCH_3_)VO(ma)]_2_, was examined to quantify its singlet–triplet gap (Fig. 12). The unpaired electrons of the vanadium atoms are coupled through a *μ*-oxo bridge, making for a complicated correlation problem involving both static and dynamic correlation. A three-body iFCI approach, correlating all 142 electrons in the 444 orbital space, affords a singlet–triplet gap within a few tens of cm^−1^ of experiment. To achieve this result, a systematic truncation scheme was used to eliminate over 90% of the three-body contributions, based on selecting incremental terms that do not significantly affect the gap.^317^

### C. Other methods

Q-Chem contains several novel active-space methods that blend together aspects of CC and valence bond (VB) theories.^320–325^ These CCVB methods separate *n* electron pairs into arbitrary radical fragments such that the dissociation energy matches CASSCF but the computational cost is only polynomial. However, these methods are difficult to use in practice due to a nonlinear wave function *ansatz* and a lack of orbital invariance, which leads to a challenging multiple-minimum problem in the orbital optimization. The CCVB-SD method^326^ restores invariance with respect to orbital mixing within the core, active-occupied, active-virtual, and inactive-virtual subspaces while retaining the desirable formal features of the CCVB expansion. Q-Chem 5 contains a production-level implementation of the CCVB-SD energy and gradient^327^ using the same tensor tools used in Q-Chem’s efficient implementation of other CC methods.^12^ As such, the cost of CCVB-SD is nearly identical to CCSD, but the former can tackle strongly correlated systems. It is natural to use CCVB-SD with an active space because it can describe both strong and weak correlations but not simultaneously. See Ref. 327 for recent applications of CCVB-SD.

Direct variational determination of the two-electron reduced density matrix (2RDM) provides an efficient description of many-electron systems that naturally captures strong correlation effects. The variational 2RDM (v2RDM) approach can be used as a driver for approximate CASSCF calculations with polynomial scaling.^328,329^ Q-Chem 5 supports v2RDM-driven CASSCF calculations in which the active-space 2RDM is constrained to satisfy two-particle (“PQG”) positivity conditions,^330^ partial three-particle conditions,^331^ or else full three-particle *N*-representability conditions.^332^ Using PQG conditions only, v2RDM-driven CASSCF can be applied to systems with active spaces as large as (64, 64).^333^ Analytic energy gradients are available for v2RDM-CASSCF calculations with all three choices of *N*-representability conditions.^334^

## V. SPECIALIZED METHODS

This section highlights some specialized features of contemporary interest. Quantum chemistry is witnessing a surge of interest in x-ray spectroscopy,^192,335–339^ fueled by advanced light sources and free-electron lasers, and by the recent availability of tabletop laser sources with femtosecond time resolution.^340–344^ For that reason, we highlight Q-Chem’s capabilities for core-level spectroscopy in Sec. V A. Q-Chem also features a suite of methods for describing metastable resonances, which are more often handled with specialized scattering codes, and Q-Chem’s functionality here is unique among widely used electronic structure packages. Unlike bound states, resonance wave functions are not square-integrable, and their description requires specialized methods based on non-Hermitian quantum mechanics,^345^ which are summarized in Sec. V B. Methods for vibronic lineshapes are described in Sec. V C, and Sec. V D describes the nuclear–electronic orbital method for the description of proton quantum effects.

### A. Modeling core-level spectroscopy

**FIG. 13.**
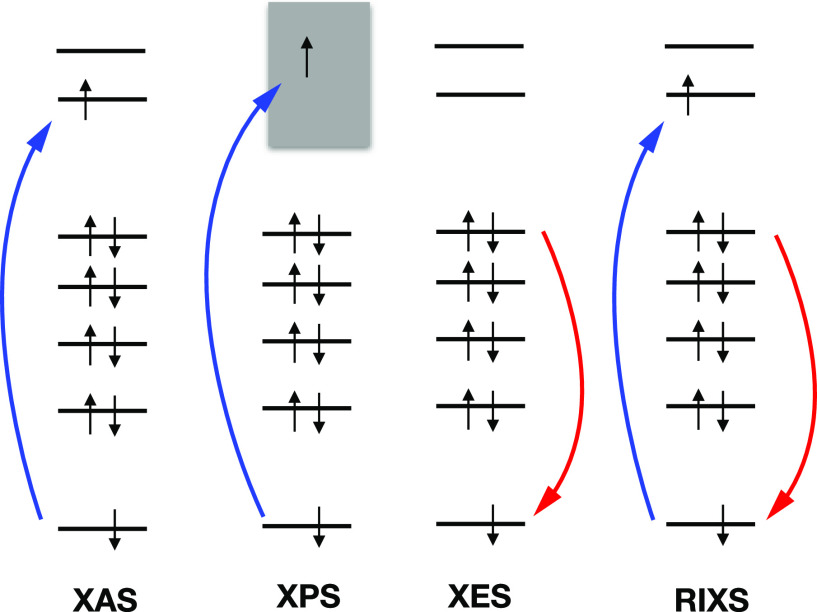
Schematic illustrations of core-level phenomena. The XAS and XPS processes involve excitation into a virtual bound molecular orbital or into the continuum, respectively, whereas the XES signal is produced by radiative relaxation of a valence electron into a core hole. The nonlinear RIXS phenomenon can be described as a coherent combination of XAS and XES transitions.

Various core-level (x-ray) processes are illustrated schematically in Fig. 13. These include x-ray absorption (XAS), x-ray emission (XES), resonant inelastic x-ray scattering (RIXS), and x-ray photoelectron spectroscopy (XPS). The relaxation of the core-level states can also result in secondary ionization, giving rise to Auger spectroscopy. These techniques correspond to photon energies above 200 eV such that core-to-valence excitations are embedded in an ionization continuum. Standard quantum chemistry approaches require modification in order to deal with these highly energetic excitations,^192,335^ especially in models with double (and higher) excitations that allow core-level states to decay. Because core-level states are Feshbach resonances that decay via two-electron processes, attempts to solve unmodified EOM-CCSD or ADC equations for core-level states lead to the same physically correct but practically disastrous behavior as attempts to describe transient anions (e.g., $N_{2}^{-}$, ${CO}_{2}^{-}$) by standard bound-state methods.^257,346^ In both cases, the solutions depend strongly on basis set (which affects how the continuum is discretized),^346^ and in the limit of a complete basis set, these states dissolve into the continuum.^257,346,347^

The ionization continuum can be projected out using the core/valence separation (CVS) scheme,^348^ which entails pruning the target Fock space by removing the configurations that do not engage the core electrons. By doing so, CVS effectively blocks the ionization channels, artificially making core-excited states bound with respect to electron loss. In addition, CVS removes the large manifold of valence excited states so that core-level excitations appear at the bottom of the excited-state manifold, within easy reach of standard iterative eigensolvers. Uncontracted or otherwise specialized basis sets are sometimes required,^192,197,349–354^ because standard Gaussian basis sets are designed for valence chemistry and may not describe the strong orbital relaxation induced by the creation of the core holes. (TDDFT is considerably less sensitive in this regard, however.^121,351^) In addition, relativistic effects and spin–orbit coupling become important for L- and M-edge excitations.^338^

Q-Chem offers a variety of methods for computing transitions involving core orbitals and the corresponding spectroscopic properties. These can be classified as follows:

• Calculations based on orbital eigenvalue differences, often using fractional orbital occupations.^355–359^
• State-specific ΔSCF methods^197,200,337^ (or ΔMP2, etc.) and spin-recoupled ROKS methods^209,211^ based on a non-*aufbau* determinant containing an orbital-relaxed core hole.
• Non-orthogonal CIS (NOCIS), which employs relaxed core holes and returns a spectrum of core excitation energies.^360–362^
• LR-TDDFT calculations using a restricted excitation window.^189,191,337,363^ In conjunction with a non-*aufbau* reference determinant, this approach can also be used to simulate XES.^364^
• Real-time TDDFT calculations of an entire broadband excitation spectrum (Sec. II C 3).
• Correlated methods within the CVS scheme, such as CVS-ADC^258,259^ and CVS-EOM-CC,^260,365–367^ for XAS, XPS, XES, x-ray electronic circular dichroism (or simply XCD), RIXS, and Auger spectroscopy. These may also be used with a non-*aufbau* reference determinant to simulate excited-state XAS and XPS, as needed in the context of time-resolved experiments.^364,368–370^

With the exception of real-time TDDFT, each of these methods invokes some sort of decoupling from the valence continuum. Neglecting the valence continuum is an approximation, which can affect the position of the core-level resonances. Apart from fully time-dependent treatment, the effect of the continuum can also be incorporated via the Feshbach–Fano formalism by combining the CVS treatment with the continuum orbitals^371^ or with other non-Hermitian methods described in Sec. V B.

Methods based on SCF eigenvalue differences *ϵ*_*a*_ − *ϵ*_*i*_ have their origins in Slater’s transition method,^372,373^ which is based on a proof that *ϵ*_*a*_ − *ϵ*_*i*_ is the leading-order approximation to a true excitation energy if the SCF calculation is performed with fractional occupation numbers *n*_*i*_ = 1/2 = *n*_*a*_. Due to the impracticality of computing an entire spectrum state-by-state, it is often assumed that the potential generated by placing 1/2 electron in the LUMO will approximately mimic that obtained by placing 1/2 electron into a higher-lying virtual orbital so that only a single fractional-electron SCF calculation is required. This approach is usually known as the *transition potential method*.^355–357^ Other occupancy schemes have sometimes been considered,^359,374,375^ with names such as “half core-hole,” “full core-hole,” and “excited core-hole.”^375^

The state-specific ΔSCF approach was described in Sec. II C 4. Here, the requisite non-*aufbau* determinant (containing a core hole) can be optimized using one of several algorithms that are available in Q-Chem, including MOM,^197^ IMOM,^198^ SGM,^199^ or STEP.^200^ This approach accounts for orbital relaxation and works very well for core-level ionization (XPS), but in the context of XAS it suffers from the same impracticality that limits Slater’s transition method. State-specific calculations are most commonly performed at DFT levels of theory (hence ΔSCF), but in principle a non-*aufbau* Hartree–Fock determinant could be used as a reference state for a subsequent wave function treatment of correlation, e.g., ΔMP2 or ΔCCSD.^197,200^ It should be kept in mind that non-*aufbau* determinants do suffer from spin-contamination (see Sec. II C 4) and sometimes from artificial symmetry breaking. The convergence of CC methods can sometimes be problematic when using a highly excited reference state.^376^

Regarding LR-TDDFT, it is worth noting that workhorse functionals for the ground-state SCF problem, which might be accurate to 0.2–0.3 eV for valence excitation energies,^128^ afford much larger errors where core-level excitation energies are concerned, e.g., shifts >10 eV are typically required using B3LYP.^377^ (That said, a recent benchmark study suggests that these large shifts do not dramatically affect the *precision* of LR-TDDFT excitation energies,^378^ such that the features of a shifted spectrum might be acceptable.) To improve the absolute accuracy, early studies suggested increasing the fraction of Hartree–Fock exchange in B3LYP to 50%–70%^189,364,379–381^ in order to balance core and valence self-interaction, but such severe modification makes these functionals inappropriate for application to valence chemistry.

An alternative is to use range separation to dial in a large fraction of exact exchange on very short length scale (<1 Å), preserving the balance of semilocal vs Hartree–Fock exchange at larger distances. This is the basis of *short-range corrected* (SRC) functionals developed specifically for x-ray spectroscopy,^189,388^ which afford an absolute accuracy of ∼0.3 eV for core-level excitations of second-row elements when used with LR-TDDFT.

**FIG. 14.**
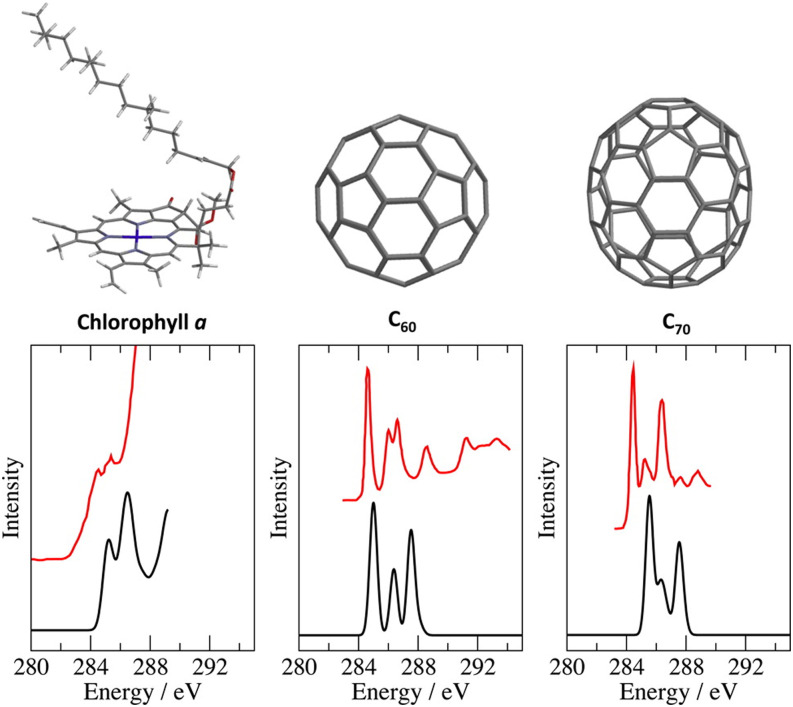
Carbon K-edge spectra for several large molecules computed with LR-TDDFT (SRC2 functional^189^ and 6-31G* basis set,^382,383^ in black) in comparison to experimental near-edge x-ray absorption fine structure (NEXAFS, in red). The experimental data are from Refs. 384–387. Reprinted with permission from N. A. Besley, J. Chem. Theory Comput. **12**, 5018 (2016). Copyright 2016 American Chemical Society.

Q-Chem has the capability to perform LR-TDDFT calculations that are optimized for XAS, reducing both the computational time and memory requirements.^191,192^ Examples of what is feasible with this approach, using a restricted excitation window approximation (analogous to the CVS approximation) at the carbon K-edge, are shown in Fig. 14. These spectra were computed at the TD-SRC2^189^/6-31G*^382,383^ level of theory and are compared directly to experiment,^384–387^ without empirical shifts.

**FIG. 15.**
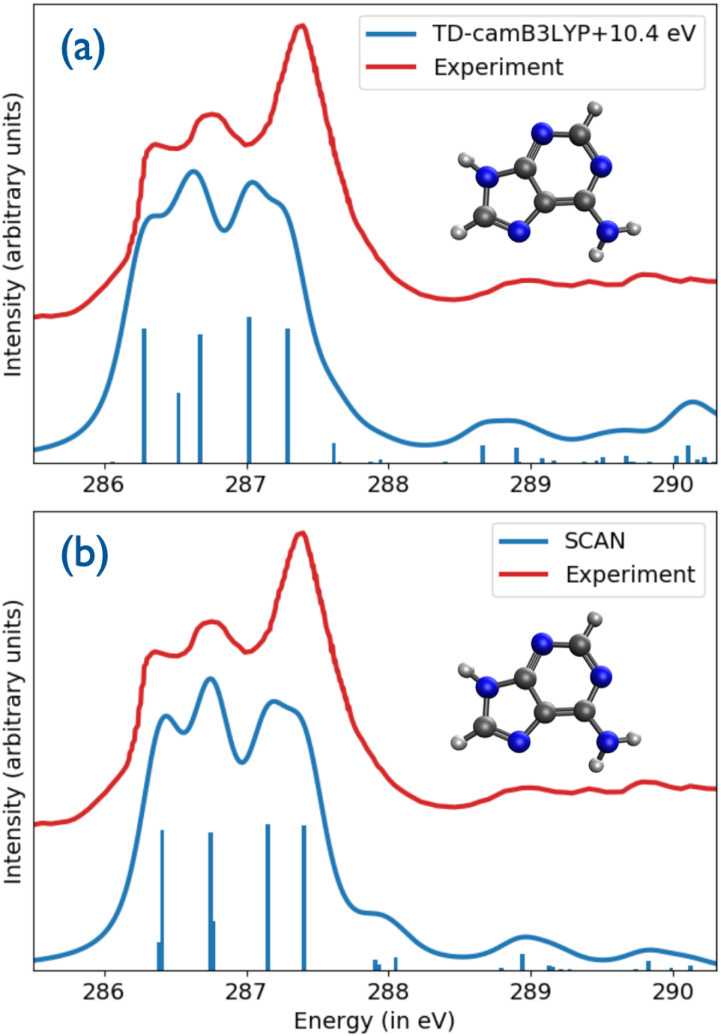
Carbon K-edge spectra of adenine obtained using (a) LR-TDDFT with the CAM-B3LYP functional^389^ vs (b) state-specific ROKS calculations using the SCAN functional. All calculations used a mixed basis set consisting of aug-cc-pCVTZ^390^ on the core-excited atom and aug-cc-pVDZ^391,392^ on all other atoms. The LR-TDDFT calculations require a 10.4 eV shift to align the low-energy edge of the calculated spectrum with experiment,^393^ whereas the ROKS spectrum is unshifted.

Whereas ΔSCF calculations are a single-determinant approximation for the excited state, ROKS calculations provide a spin-pure treatment of open-shell singlet excited states, as discussed in Sec. II C 4, while also providing full core-hole relaxation. ROKS with Hartree–Fock orbitals attains a root-mean-square error (RMSE) of 0.6 eV for K-edge excitations of second-row elements,^212^ *without any correlation*, highlighting the importance of orbital relaxation in describing core-level states. Inclusion of dynamic correlation via DFT can lead to better results, with the modern SCAN meta-GGA^40^ affording a RMSE of ∼0.2 eV for K-edge excitations of C, N, O, and F.^209^ Similarly, small errors are obtained at the L-edges of third-row elements.^209^ The relatively low computational scaling of the semilocal SCAN functional (as compared to hybrid DFAs) makes this approach particularly appealing for larger systems. While it might appear tedious to optimize each possible excitation individually with ROKS, the suite of excited state orbital optimization methods in Q-Chem permits explicit computation of a full spectrum without too much difficulty. This is demonstrated in Fig. 15, which depicts the carbon K-edge spectrum of adenine computed via ROKS using the SCAN functional and the SGM algorithm.

It is also possible to compute multiple excited states simultaneously while accounting for core–hole relaxation. The non-orthogonal CIS (NOCIS) approach achieves this by performing CIS with relaxed orbitals for the core-ionized state.^360,362^ Specifically, NOCIS computes optimal core-ionized orbitals for each possible atomic core-excitation site, builds all singly excited configurations that preserve the desired core hole, and then diagonalizes the Hamiltonian within the subspace spanned by these (non-orthogonal) determinants. Some additional considerations involving ΔSCF states are necessary to extend NOCIS to open-shell systems,^361,362^ and the lack of dynamic correlation leads to small (0.5–1.0 eV) overestimation of excitation energies. However, these drawbacks should be balanced against the ability to compute multiple excited states simultaneously, which is not possible with the more accurate ROKS approach. Much efficiency is gained and almost no accuracy is lost by restricting the CI space to individual atoms.^362^

Finally, many-body methods, such as ADC^227^ and EOM-CC,^224^ provide the means to compute core-excited transitions with systematically improvable accuracy. These methods include both orbital relaxation and electron correlation in a single computational step, within a multi-state formalism that naturally affords transition properties. These methods are naturally spin-adapted when used with a closed-shell reference determinant. Q-Chem 5 facilitates calculation of XPS, XAS, and XES using the CVS-EOM-IP-CCSD approach^260,366^ and XAS using either CVS-EOM-EE-CCSD^260,366^ or CVS-EE-ADC.^259,394,395^

**FIG. 16.**
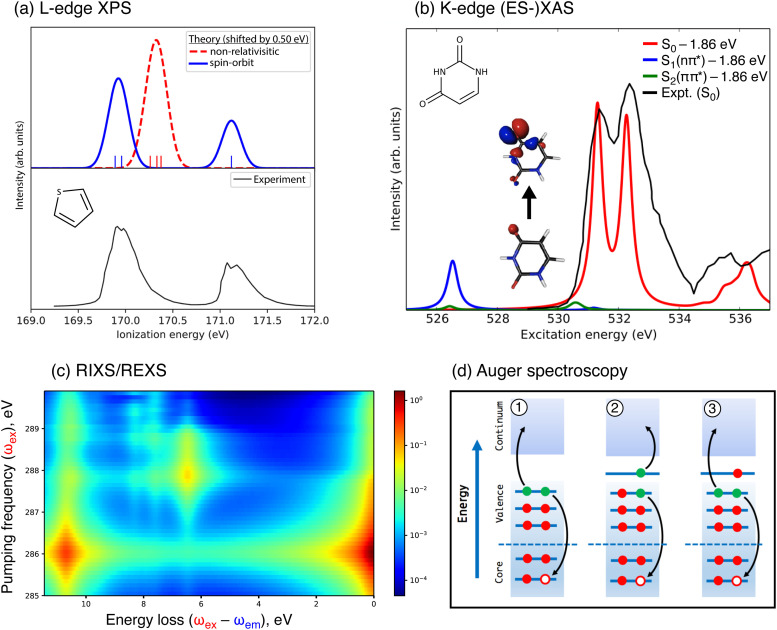
Exemplary applications of CVS-EOM-CCSD methods to x-ray spectroscopy. (a) Sulfur L-edge XPS spectra of thiophene with and without spin–orbit coupling computed at the fc-CVS-EOM-CCSD/u6-311+G(3df) level. The notation u6-311+G(3df) indicates an uncontracted version^197,353^ of 6-311+G(3df).^396–398^ (b) Oxygen K-edge XAS spectra of uracil in its S_0_, S_1_, and S_2_ states computed at the fc-CVS-EOMEE-CCSD/6-311++G** level. Intensity of the excited state bands has been reduced assuming 15% population. NTOs of the 1s → SOMO transition in S_1_ are also shown. (c) RIXS/REXS two-dimensional energy-loss spectrum of benzene vs pumping frequency *ω*_ex_ computed at the fc-CVS-EOM-CCSD/u6-311(2+,+)G** level. Intensities are on a logarithmic scale. (d) Illustrations of various Auger effects: (1) regular Auger decay, (2) resonant (participator) decay, and (3) resonant (spectator) decay. Regular Auger decay is relevant for XPS, whereas resonant Auger processes occur in XAS. These processes can be modeled within the Feshbach–Fano framework using CVS-EOM-CC to describe the initial core-excited or core-ionized state and EOM-IP-CC or DIP-CC to describe the final state. Panel (a) is adapted with permission from Vidal *et al.*, J. Phys. Chem. Lett. **11**, 8314 (2020). Copyright 2020 American Chemical Society. Panel (b) is adapted from Vidal *et al.*, J. Chem. Theory Comput. **15**, 3117 (2019). Copyright 2019 American Chemical Society. Panel (c) is reproduced with permission from Nanda *et al.* Phys. Chem. Chem. Phys. **22**, 2629 (2020). Published by the PCCP Owner Societies. Panel (d) is reproduced from W. Skomorowski and A. I. Krylov, J. Chem. Phys. **154**, 084124 (2021) with the permission of AIP Publishing.

CVS-EOM methods combined with spin–orbit coupling have been used to compute L-edge XPS,^369^ as in Fig. 16(a). Time-resolved variants of XPS or XAS can be modeled by using a non-*aufbau* reference determinant^366,368,370^ or directly as transitions between target ADC/EOM states,^260,368^ as illustrated in Fig. 16(b). Nonlinear spectra, including RIXS, can also be computed with correlated methods,^367,399^ as in Fig. 16(c). Features such as Dyson orbitals,^264,366^ attachment/detachment densities,^400^ and NTOs^15,88,266,267,401^ facilitate analysis and interpretation of the computed spectra. A unique feature of Q-Chem is the ability to compute Auger decay rates and Auger spectra using the Feshbach–Fano formalism combined with CVS-EOM-CC and an explicit description of the free electron,^371^ as illustrated in Fig. 16(d).

### B. Methods for metastable resonances

Electronic resonances, meaning states that are unstable with respect to electron loss, are ubiquitous in energetic environments such as plasmas, in combustion, and in the presence of ionizing radiation.^257,345^ Resonances are also relevant to condensed-phase processes under milder conditions, e.g., plasmonic catalysis,^402^ and may play a role in radiation-induced damage to living tissue.^403^ Because resonances lie in the continuum, their wave functions are not square-integrable and cannot be described using standard quantum-chemical methods designed for isolated bound states. Naïve application of bound-state quantum chemistry to metastable states does not capture genuine resonances but rather “orthogonalized discretized continuum states,”^346^ where the metastable state behaves like a poor approximation to a plane wave, trapped by a finite Gaussian basis set, with properties that are artificial and prone to change erratically as the basis set is changed, especially if additional diffuse functions are introduced.

This computational predicament is elegantly circumvented within non-Hermitian quantum mechanics based on complex-variable techniques,^345^ which generalizes and extends concepts from bound-state quantum chemistry to the case of electronic resonances.^257,345,346^ Within this modified formulation, electronic resonances *can* be described as square-integrable quasi-stationary states albeit with complex-valued energies, *E* = *E*_*R*_ − iΓ/2, where *E*_*R*_ is the resonance position and Γ is the resonance width, the latter of which arises from lifetime broadening.

Q-Chem offers three different complex variable techniques: complex coordinate scaling (CS),^346,404–409^ complex basis functions (CBFs),^410–413^ and complex absorbing potentials (CAPs).^414–417^ The CS approach regularizes the resonance wave function by rotating all coordinates in the Hamiltonian into the complex plane, *x* → *xe*^i*θ*^. This approach has a rigorous mathematical foundation but is not compatible with the Born–Oppenheimer approximation, limiting its applicability to atoms, whereas CBFs and CAPs are applicable to molecules. (The latter approaches can be considered as approximations to “exterior” CS.^418,419^) CBF methods utilize mixed basis sets in which the exponents of the most diffuse functions are complex-scaled, whereas the CAPs simply add an imaginary potential to the molecular Hamiltonian ${\hat{H}}_{0}$,

$$\hat{H}={\hat{H}}_{0}+\text{i}\hat{W}\left(x\right).$$ (15)

The CAP serves to absorb the non-normalizable tail of the resonance wave function, and several functional forms for $\hat{W}$(**x**) are available in Q-Chem. Although there is some arbitrariness associated with the details of the CAP, these methods are generally easier to use as compared to alternative “stabilization” methods,^346,420,421^ in which Gaussian exponents or atomic numbers are modified in order to stabilize the resonance (making it amenable to standard bound-state methods), with the results then extrapolated back to the physical system of interest. If applied carefully, both the stabilization and CAP methods afford useful results;^422^ however, the CAP approach is more rigorous and more straightforward to extend to other molecular properties.

The CS, CBF, and CAP techniques can each be combined with the full EOM-CCSD suite of methods implemented in Q-Chem. The CAP technique is also available for all ADC methods,^248^ implemented via a subspace projection approach.^423^ The EOM-EA or EA-ADC variants are appropriate for treating metastable radical anions of closed-shell molecules, whereas super-excited states of neutral molecules and metastable excited states of closed-shell anions are best described using EOM-EE or EE-ADC.

Q-Chem offers several functionalities for the characterization of electronic resonances beyond their positions and widths, including

• first-order one-electron state properties and transition moments for all complex-variable EOM-CC methods,^415,424^
• Dyson orbitals for all complex-variable EOM-CC methods,^424–426^
• NTOs for CAP-EOM-CC methods,^427^ and
• analytic gradients for CAP-EOM-CC methods.^428^

These tools are useful for investigating the spectroscopy and chemical reactivity of electronic resonances. Dyson orbitals and NTOs, for example, provide compact representations of changes in the wave function upon electron attachment or electronic excitation. Since complex-valued Hamiltonians are not Hermitian but rather complex-symmetric, these quantities conform to a modified metric in which the real part of the complex electron density integrates to the number of electrons, while its imaginary part integrates to zero.^406^ Related results hold for density matrices, transition density matrices, orbitals, and wave functions, all of which also feature a real and an imaginary part. Analogous to the case of bound states, a singular value decomposition of the one-electron transition density matrix affords pairs of NTOs, which facilitate the interpretation of an electronic excitation in terms of MO theory.^269^

**FIG. 17.**
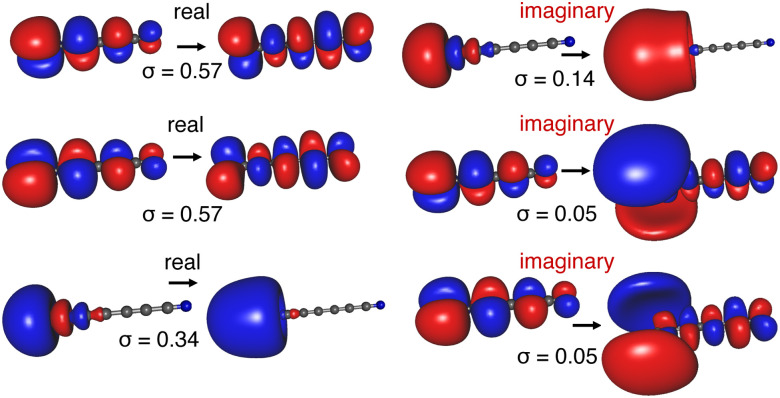
Real and imaginary NTOs for the ^1^Σ^+^ resonance in C_7_N^−^. This state has mixed *π* → π* and *σ* → σ* character, as apparent from the participation ratio PR_NTO_(*γ*^Re^) ≈ 3. Based on the singular values $\sigma_{K}^{\text{Im}}$, the total width of 0.13 eV can be separated into two contributions, Γ_Σ_ = 0.10 eV and Γ_Π_ = 0.03 eV, corresponding to the two decay channels in which the C_7_N radical is either formed in the ^2^Σ^+^ or the ^2^Π state. Reprinted with permission from W. Skomorowski and A. I. Krylov, J. Phys. Chem. Lett. **9**, 4101 (2018). Copyright 2018 American Chemical Society.

Further analysis of NTOs and exciton wave functions can be accomplished based on the Feshbach formalism,^429^ wherein a resonance is described as a bound state coupled to a continuum of scattering states. This analysis demonstrates that the real part of the excitonic wave function describes changes in the electron density corresponding to the bound part of the resonance, while the imaginary component of the wave function can be interpreted as virtual states that facilitate one-electron decay into the continuum.^427^ Singular values associated with particular NTOs can be related to the partial widths of the respective decay channels. As an example, Fig. 17 illustrates NTOs for the ^1^Σ^+^ resonance in C_7_N^−^, a chain-like cyanopolyyne anion relevant to astrochemistry.^430^

**FIG. 18.**
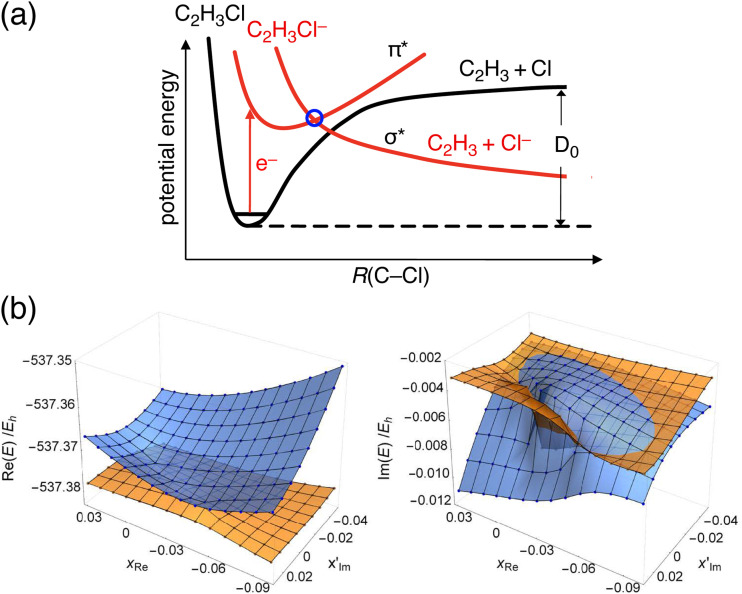
(a) Schematic representation of dissociative electron attachment to chloroethylene. The exceptional point is marked by a blue circle. (b) Real and imaginary part of the potential surfaces in the vicinity of the minimum-energy exceptional point between the π* and the σ* states of the chloroethylene anion, plotted above the plane spanned by the real gradient difference vector (**x**_Re_) and the imaginary gradient difference vector orthogonalized to **x**_Re_ ($x_{\text{Im}}^{\prime }$). Reprinted with permission from Z. Benda and T.-C. Jagau, J. Phys. Chem. Lett. **9**, 6978 (2018). Copyright 2018 American Chemical Society.

Analytic gradients enable the search for special points on the complex-valued potential surfaces of polyatomic resonances. Algorithms are available for equilibrium structures,^428,431^ for crossings between resonances and their parent states,^432^ and for crossings between two resonances,^433^ the latter of which are known as *exceptional points*. These critical points govern the nuclear dynamics following the formation of a resonance state and, if that resonance is long-lived enough, can be connected to features in electron transmission and energy-loss spectra. In particular, exceptional points may be considered the non-Hermitian analogs of conical intersections and play a similar role for electron-induced chemistry as conical intersections do for photochemistry.^433^ An example involving a dissociative electron attachment process^434–436^ is considered in Fig. 18, in which a ${\left(\pi^{*}\right)}^{-}$ resonance anion state is accessible at the equilibrium structure of the neutral parent molecule, chloroethylene.^433^ The dissociative state has ${\left(\sigma^{*}\right)}^{-}$ character but is too high in energy to be accessed directly, and the reaction proceeds via nonadiabatic transition between the two resonances, along a seam of exceptional points. The complex-valued potential surfaces for the ${\left(\sigma^{*}\right)}^{-}$ and ${\left(\pi^{*}\right)}^{-}$ resonances around the minimum-energy exceptional point are shown in Fig. 18, computed using CAP-EOM-EA-CCSD.

### C. Calculation of vibronic lineshapes

The vibrational structure of electronic transitions encodes rich information about molecular structure, in both linear spectroscopies (UV–Vis, XAS, XPS, etc.) and nonlinear ones (2PA, RIXS, resonance Raman, etc.). Quantitative modeling of these spectra combines calculations of electronic structure and nuclear wave functions via either a static (time-independent) or a dynamic (time-dependent) formalism.^437–443^ Q-Chem 5 provides several capabilities to calculate the vibrationally resolved spectra and certain types of electronic cross sections.

Within the dipole approximation, the probability of transition between an initial state (*i*) and a final state (*f*) is proportional to the square of the transition dipole matrix element,

$$P_{if}\propto {\left(\int \Psi_{i}\left(r,R\right)\;\hat{\mu }\;\Psi_{f}\left(r,R\right)\;\text{d}r\;dR\right)}^{2},$$ (16)

when the photon is resonant with the energy gap. Here, $\hat{\mu }$ is the electronic dipole moment operator, and coordinates **R** and **r** represent nuclei and electrons, respectively. Within the Born–Oppenheimer approximation,^444,445^ the wave functions Ψ(**r**, **R**) can be factored into a nuclear wave function *χ*(**R**) and an electronic wave function *ψ*(**r**; **R**) so that

$$P_{i^{\prime }f^{\prime \prime }}\propto {\left(\int \psi_{i}\left(r;R\right)\chi_{i^{\prime }}\left(R\right)\;\hat{\mu }\;\psi_{f}\left(r;R\right)\chi_{f^{\prime \prime }}\left(R\right)\;\text{d}r\;dR\right)}^{2}.$$ (17)

Indices *i*′ and *f*″ denote the vibrational states of the two electronic states. Within the Born–Oppenheimer approximation, the vibrational wave functions are determined solely from the nuclear Schrödinger equation with a potential defined by the electronic Schrödinger equation. Integration over the electronic coordinates in Eq. (17) affords the electronic transition dipole moment for the *i* → *f* transition,

$$\mu_{if}\left(R\right)=\int \psi_{i}\left(r;R\right)\;\hat{\mu }\;\psi_{f}\left(r;R\right)\;\text{d}r.$$ (18)

The transition probability can therefore be written as

$$P_{i^{\prime }f^{\prime \prime }}\propto {\left(\int \chi_{i^{\prime }}\left(R\right)\;\mu_{if}\left(R\right)\;\chi_{f^{\prime \prime }}\left(R\right)\;\text{d}R\right)}^{2}.$$ (19)

Equation (19) is the basis for modeling the spectrum. It contains an electronic transition moment ***μ***_*if*_(**R**) in addition to vibrational wave functions for the initial and final states.

Within the Condon approximation,^446^ it is assumed that ***μ***_*if*_(**R**) depends weakly on the nuclear coordinates so can be evaluated at a fixed nuclear geometry, e.g., at the equilibrium geometry **R**_*e*_ of the initial state. Then,

$$P_{i^{\prime }f^{\prime \prime }}\propto {\left\|\mu_{if}\left(R_{e}\right)\right\|}^{2}{\left(\int \chi_{i^{\prime }}\left(R\right)\;\chi_{f^{\prime \prime }}\left(R\right)\;\text{d}R\right)}^{2}.$$ (20)

The overlap integral between the two nuclear wave functions is called a *Franck–Condon factor* (FCF),^441,446–448^ which is directly related to the intensities of vibrational progressions via Eq. (20).

FCFs for various spectroscopic transitions (photoelectron, UV–Vis, etc.) can be computed in a post-processing step using the ezFCF module of the stand-alone software ezSpectra,^449^ which implements FCFs within the double-harmonic approximation, either with or without consideration of Duschinsky rotation,^441,450^ i.e., changes in the normal modes between the ground and excited electronic states. These calculations require optimized structures and normal mode analysis for both electronic states but are completely agnostic regarding the level of electronic structure theory at which these calculations are performed. ezSpectra also contains a module ezDyson, which can be used to compute total and angular-resolved photoelectron spectra. This requires Dyson orbitals that can be computed using Q-Chem.

To go beyond the Condon approximation, one can invoke the Herzberg–Teller (HT) normal mode expansion of ***μ***_*if*_(**R**) around the equilibrium nuclear geometry,^440,441,451^ in order to account for geometry-dependent changes in the transition dipole moment. Although the Condon approximation is generally accurate for strongly allowed transitions for weak or forbidden transitions, the Franck–Condon term [Eq. (20)] is nearly or exactly zero, and therefore higher-order terms may become important. These give rise to the HT effect.^440,441^

**FIG. 19.**
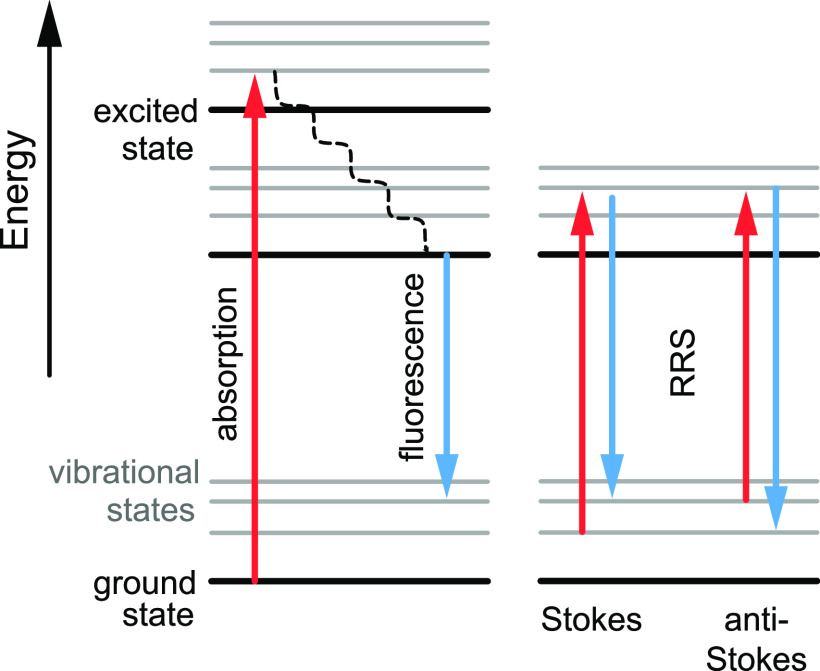
Schematic diagram for one-photon absorption and one-photon emission (left) and for resonance Raman scattering (RRS, at right).

Raman scattering is a two-photon process (see Fig. 19), and resonance Raman scattering (RRS) is a particular type of vibrational Raman spectroscopy in which the incident laser frequency lies close to an electronic transition.^452,453^ In RRS, an incident photon with frequency *ω*_L_ (the laser frequency) is absorbed and another with frequency *ω*_S_ is emitted, with the difference corresponding to a vibrational level spacing. The differential photon scattering cross section is given by^442,454–456^

$$\sigma \left(\omega_{\text{L}},\omega_{\text{S}}\right)\propto \omega_{\text{L}}\omega_{\text{S}}^{3}S\left(\omega_{\text{L}},\omega_{\text{S}}\right),$$ (21)

where

$$S\left(\omega_{\text{L}},\omega_{\text{S}}\right)={\left\|\left\langle \psi_{f}|\hat{M}|\psi_{i}\right\rangle \right\|}^{2}\delta \left(\omega_{\text{S}}-\omega_{\text{L}}+\omega_{fi}\right)$$ (22)

and the transition operator

$$\hat{M}=\underset{k}{\sum }\left[\frac{\hat{\mu }\cdot e_{2}|\psi_{k}〉〈\psi_{k}|\hat{\mu }\cdot e_{1}}{\omega_{\text{L}}-\omega_{ki}}-\frac{\hat{\mu }\cdot e_{1}|\psi_{k}〉〈\psi_{k}|\hat{\mu }\cdot e_{2}}{\omega_{\text{S}}+\omega_{ki}}\right]$$ (23)

involves a sum over virtual vibronic states *k*. In the RRS process, the initial (*i*) and final (*f*) electronic states both correspond to the ground state, so *ℏω*_*fi*_ represents a difference between ground-state vibrational energy levels, as depicted in Fig. 19. When the energy gap *ω*_*k*_ − *ω*_*i*_ between the *k* state and the *i* state is close to the laser frequency *ω*_L_, the intermediate state *k* (a vibrational level of an excited electronic state) dominates the scattering cross section and non-resonant contributions can be neglected.

The formalism described above is inconvenient because even in the resonant case where only a single excited electronic state is important, Eq. (23) still requires a sum over vibrational levels on that state. An alternative strategy is based on a time-dependent formalism,^457,458^ which circumvents the evaluation of the multidimensional integrals that appear when FCFs are computed beyond the parallel-mode approximation, i.e., when Duschinsky rotation is included. In this approach, matrix elements of $\hat{M}$ (which generates the polarizability tensor) are avoided and the scattering cross section is expressed in terms of the Fourier transform of a time correlation function representing the overlap between the final state |*ψ*_*f*_⟩ and the time-evolving wave function |Ψ(*t*)⟩ following excitation to the upper electronic state,

$$\sigma \left(\omega_{\text{L}}\right)\propto \int_{0}^{\infty }e^{\text{i}\omega_{\text{L}}-\Gamma t}\left\langle \psi_{f}|\Psi \left(t\right)\right\rangle \;dt+\text{NRT}.$$ (24)

(Here, “NRT” denotes the non-resonant terms that can be neglected in RRS, and Γ is a damping factor.) A detailed theoretical background is given in Ref. 442.

**FIG. 20.**
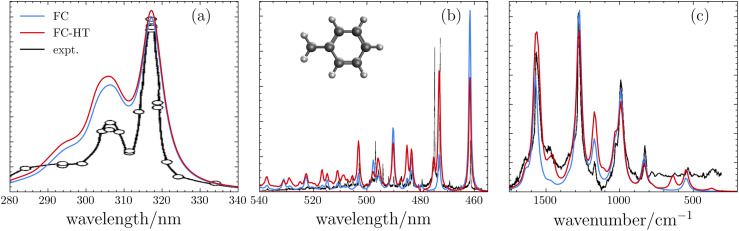
(a) Absorption spectra, (b) emission spectra, and (c) RRS spectra of benzyl radical, comparing experimental results to calculations within the FC approximation (in blue) vs the FC-HT approximation (in red). A damping factor of Γ = 300 cm^−1^ and temperature *T* = 298 K were used for the absorption spectrum vs Γ = 20 cm^−1^ and *T* = 0 K for the emission spectrum. For the RRS spectrum, a damping factor of 100 cm^−1^, Lorentzian broadening of Γ = 20 cm^−1^, and *T* = 298 K are used. All electronic structure calculations are performed at the (TD-)B3LYP/6-311G** level. To make the simulated spectra consistent with experiment,^459–461^ the adiabatic energy gap is shifted by 0.04 eV for absorption, −0.34 eV for emission, and −0.11 eV for RRS. The wavelength of incident light for the RRS simulation is 315 nm, as in the experiment.^460^

Q-Chem 5 includes a built-in implementation of the time-dependent correlation function approach at the LR-TDDFT level, which enables calculation of vibrationally resolved one-photon and two-photon absorption and emission spectra^462,463^ and RRS spectra^440^ within the double-harmonic approximation, including both Duschinsky rotation and HT effects in the time domain. To illustrate the capabilities of the theory, Fig. 20 compares calculated FC and FC-HT spectra for the benzyl radical to experiment. The absorption and fluorescence spectra arise from the *D*_0_ → *D*_3_ and *D*_1_ → *D*_0_ transitions, respectively. In particular, for the stimulated emission and the RRS spectra, agreement with experiment improves upon inclusion of the HT terms.

For semiquantitative calculations, a short-time approximation to Eq. (24) can be used, which turns out to be equivalent to the “independent mode, displaced harmonic oscillator” model,^438,456,464^ in which it is assumed that equilibrium displacements of the vibrational normal modes change upon electronic excitation but not the modes themselves or their frequencies. Under those assumptions, the dimensionless displacement $\Delta_{k}={\left(\omega_{k}/\hbar \right)}^{1/2}\Delta Q_{k}$ for normal mode *Q*_*k*_ can be related to the excited-state gradient, i.e., the derivative *∂*Ω/*∂Q*_*k*_ of the electronic excitation energy, Ω:^465,466^

$$\Delta_{k}=\frac{1}{\sqrt{\hbar \omega_{k}^{3}}}\left(\frac{\partial \Omega }{\partial Q_{k}}\right).$$ (25)

The relative resonant enhancement in the intensity of mode *Q*_*j*_ vs mode *Q*_*k*_ is^466^

$$\frac{I_{j}}{I_{k}}={\left(\frac{\omega_{j}\Delta_{j}}{\omega_{k}\Delta_{k}}\right)}^{2}.$$ (26)

Within this approximation, the resonant enhancement in RRS (as compared to normal Raman scattering) consists of the excited-state gradient projected onto ground-state normal modes {*Q*_*k*_}, so this approach has also been called the excited-state gradient approximation.^465,467^ It has been implemented in Q-Chem 5 for CIS and LR-TDDFT excitation energies and used to compute the resonance Raman spectra of complex systems, such as *e*^−^(aq).^466^ This approach has also been combined with *ab initio* molecular dynamics to simulate transient (excited-state) RRS,^468^ which is measurable via the emerging technique of femtosecond stimulated Raman spectroscopy.^469,470^

### D. Nuclear–electronic orbital methods

Nuclear quantum effects are essential in many chemical and biological processes, such as proton transfer and proton-coupled electron transfer reactions. The nuclear–electronic orbital (NEO) method provides a framework for the accurate and computationally efficient incorporation of the significant nuclear quantum effects within an electronic structure calculation.^471,472^ In this approach, specified nuclei are treated quantum mechanically alongside the MO description of the electrons, thereby avoiding the Born–Oppenheimer separation between the electrons and the quantum nuclei. Treating at least two nuclei classically prevents complications with translations and rotations. Typically, the quantum nuclei are chosen to be protons or deuterons, although the NEO method has also been applied to positrons.^473,474^ For simplicity, the formalism presented below assumes quantum protons. A significant advantage of the NEO method is that anharmonicity, proton delocalization, and zero-point energy are included directly in energies, geometry optimizations, reaction paths, and molecular dynamics. Both wave function and DFT methods have been developed within the NEO framework for the accurate description of nuclear quantum effects in the ground and excited states of molecular systems.^474–490^

The NEO Hamiltonian operator is^471^

$${\hat{H}}_{\text{NEO}}={\hat{T}}^{\text{e}}+{\hat{V}}^{\text{e}}+{\hat{V}}^{\text{ee}}+{\hat{T}}^{\text{p}}+{\hat{V}}^{\text{p}}+{\hat{V}}^{\text{pp}}+{\hat{V}}^{\text{ep}},$$ (27)

where ${\hat{T}}^{\text{e}}$, ${\hat{V}}^{\text{e}}$, and ${\hat{V}}^{\text{ee}}$ are the conventional electronic operators corresponding to kinetic energy, electron–nuclear attraction (for the classical nuclei only), and electron–electron repulsion, respectively. Operators ${\hat{T}}^{\text{p}}$, ${\hat{V}}^{\text{p}}$, and ${\hat{V}}^{\text{pp}}$ represent the analogous quantities for the quantum protons. Finally, ${\hat{V}}^{\text{ep}}$ is the operator corresponding to the electron–proton Coulomb interaction. Simultaneous mean-field descriptions of both the electrons and the quantum protons results in the NEO-Hartree–Fock *ansatz*,^471^ but unfortunately the omission of electron–proton correlation effects makes this model inadequate for predictions of reliable energies or geometries.^472^ The rest of this section describes DFT-based alternatives.

#### 1. NEO-DFT

The NEO-DFT method is a multicomponent extension of the conventional electronic DFT formalism, in which different types of particles (e.g., electrons and protons) are treated quantum mechanically.^491–493^ Similar to NEO-HF, the NEO-DFT Kohn–Sham wave function is the product of electronic and protonic Slater determinants composed of the Kohn–Sham spin orbitals. The NEO-DFT energy is

$$\begin{array}{ll} E\left[\rho^{\text{e}},\rho^{\text{p}}\right] & =E_{\text{ext}}\left[\rho^{\text{e}},\rho^{\text{p}}\right]+E_{\text{ref}}\left[\rho^{\text{e}},\rho^{\text{p}}\right]+E_{\text{exc}}\left[\rho^{\text{e}}\right]\\ & \;+\;E_{\text{pxc}}\left[\rho^{\text{p}}\right]+E_{\text{epc}}\left[\rho^{\text{e}},\rho^{\text{p}}\right]. \end{array}$$ (28)

Here, *E*_ext_[*ρ*^e^, *ρ*^p^] is the interaction of the electronic and protonic densities, *ρ*^e^ and *ρ*^p^, with the external potential created by the classical nuclei. The term *E*_ref_[*ρ*^e^, *ρ*^p^] contains the electron–electron, proton–proton, and electron–proton classical Coulomb energies, as well as the noninteracting kinetic energies of both electrons and quantum protons. The final three terms are electron–electron XC, proton–proton XC, and electron–proton correlation functionals. Variational minimization of the NEO-DFT energy with respect to the densities leads to two sets of coupled Kohn–Sham equations for electrons and protons, which are strongly coupled and must be solved together self-consistently.

Implementation of the NEO-DFT method requires the functionals in Eq. (28). Within this framework, any conventional electron–electron XC functional can be employed.^477^ Due to the local nature of the quantum protons in molecular systems, the proton–proton XC energies are negligible,^472^ but the Hartree–Fock proton–proton exchange is included. The electron–proton correlation (epc) functional is essential for accurate calculations of proton densities and energies. The epc17 (LDA form)^475,476^ and epc19 (GGA form)^478^ functionals were formulated as extensions of the Colle–Salvetti formalism for electron–electron correlation^494,495^ to the case of electron–proton correlation. These functionals are designed to accurately describe proton densities and energies of molecular systems.

**FIG. 21.**
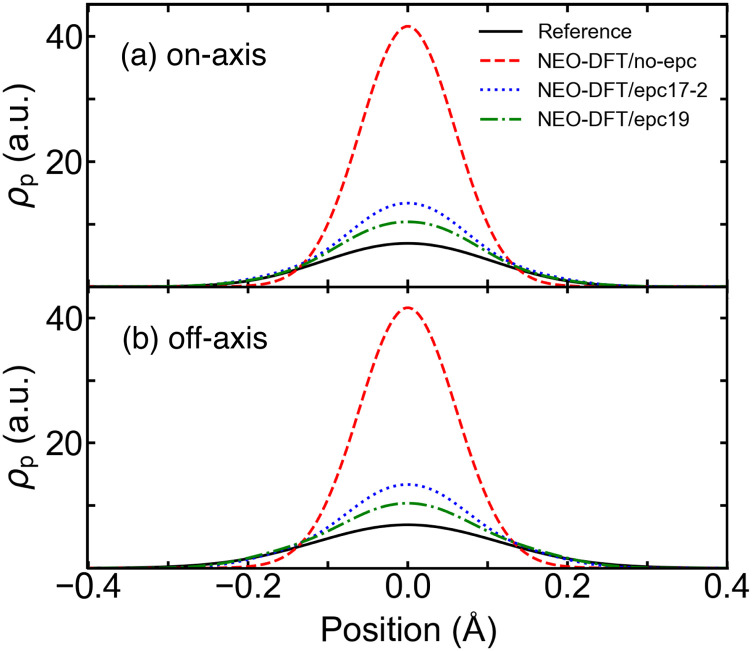
(a) On-axis and (b) off-axis proton density for FHF^−^ computing using NEO-DFT with no electron–proton correlation and two different electron–proton correlation functionals, in comparison to a grid-based reference calculation. All calculations use the B3LYP electronic functional, def2-QZVP electronic basis set,^75^ and an even-tempered 8s8p8d protonic basis set. Adapted with permission from Pavošević *et al.*, Chem. Rev. **120**, 4222 (2020). Copyright 2020 American Chemical Society.

The importance of electron–proton correlation for the prediction of accurate proton densities is shown in Fig. 21 for the FHF^−^ molecular ion, where results from NEO-DFT with several different electron–proton correlation treatments are compared to a near-exact result computed using the Fourier grid method.^496–498^ In the absence of electron–proton correlation (NEO-DFT/no-epc in Fig. 21), the proton density is much too localized, similar to NEO-HF results. Inclusion of electron–proton correlation using either the epc17-2 functional^475,476^ or the epc19 functional^478^ significantly improves the proton densities.

In addition to accurate proton densities, these two epc functionals were shown to predict accurate proton affinities for a diverse set of molecules composed of amines, carboxylates, aromatics, and inorganic species.^476,478^ Because the NEO-DFT method inherently includes the zero-point energy contributions from the quantum protons, the proton affinity of molecule A is simply

$$\text{PA}\left(\text{A}\right)=E_{\text{A}}-E_{{\text{HA}}^{+}}+\frac{5}{2}RT,$$ (29)

where *E*_A_ is the energy of A computed with conventional DFT and $E_{{\text{HA}}^{+}}$ is the energy of the protonated species calculated using NEO-DFT. This procedure does not require the calculation of computationally expensive Hessians because the zero-point energy contributions from the other nuclei have been shown to be negligible due to cancellation.^480^ Moreover, the NEO-DFT method includes the anharmonic effects associated with the quantized proton.

**FIG. 22.**
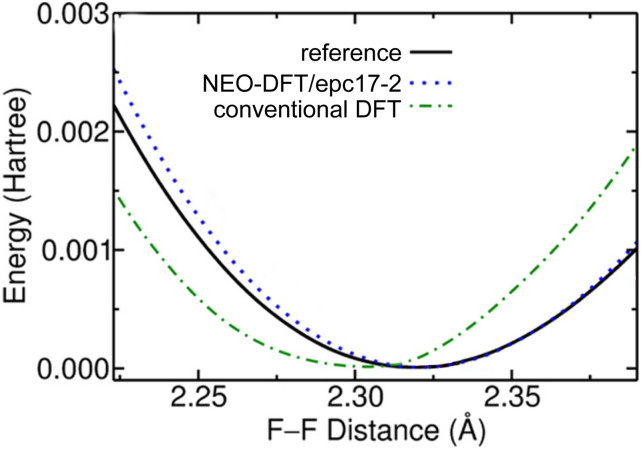
Energy as a function of F–F distance for FHF^−^, comparing conventional DFT and NEO-DFT results to a grid-based reference. Quantization of the proton increases the equilibrium F–F distance. These calculations were performed using the B3LYP electronic functional, the def2-QZVP electronic basis set, and an even tempered 8s8p8d protonic basis set. Data are from Ref. 476.

Analytic geometry gradients for the NEO-DFT method with the epc17-2 and epc19 functionals allow geometry optimizations that include the effects of proton delocalization, anharmonicity, and zero-point energy. Figure 22 shows that the NEO-DFT/epc17-2 method accurately predicts the increased F–F bond length in the FHF^−^ ion, which is shifted by ≈0.02 Å due to proton quantization.^476^ The NEO-DFT/epc17-2 method has been used to optimize the geometries of protonated water tetramers with all nine protons treated quantum-mechanically and correctly predicts the energetic ordering of the four isomers.^490^

The NEO-HF, NEO-DFT/no-epc, NEO-DFT/epc17-2, and NEO-DFT/epc19 methods are available in Q-Chem 5 in both restricted and unrestricted formalisms. The quantum protons are always assumed to be high-spin. Analytic gradients are available for each of these methods, enabling geometry optimizations. The user must specify the quantum protons, the electronic and protonic basis sets,^475,483,499^ and the electron and electron–proton correlation functionals.

#### 2. NEO-TDDFT

**FIG. 23.**
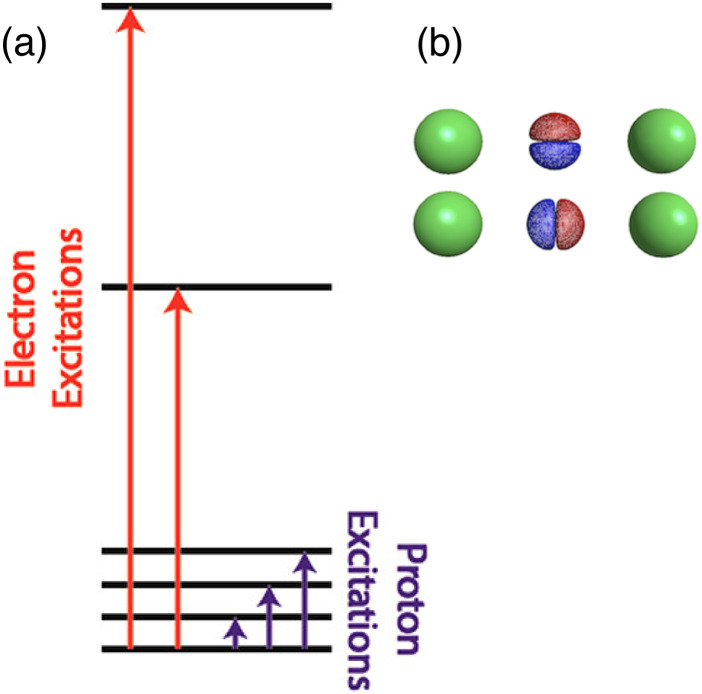
(a) Schematic depiction of the electronic and proton vibrational excitations obtained from a single NEO-TDDFT calculation. (b) Transition densities for the bend and stretch modes of FHF^−^. Panel (a) is reproduced with permission from Yang *et al.*, J. Phys. Chem. Lett. **9**, 1765 (2018). Copyright 2018 American Chemical Society. Panel (b) is reproduced from Culpitt *et al.*, J. Chem. Phys. **150**, 201101 (2019) with the permission of AIP Publishing.

NEO-TDDFT is a multicomponent extension of conventional electronic LR-TDDFT that allows for the simultaneous calculation of electronic and protonic (vibrational) excitation energies,^479^ as depicted in Fig. 23. The formalism follows from the linear response of the NEO Kohn–Sham equations to an external perturbation, and NEO-TDDFT excitation energies Ω are obtained by solving the following multicomponent equation:^479^

$$\left[\begin{array}{cccc} A^{\text{e}} & B^{\text{e}} & C & C\\ B^{\text{e}} & A^{\text{e}} & C & C\\ C^{†} & C^{†} & A^{\text{p}} & B^{\text{p}}\\ C^{†} & C^{†} & B^{\text{p}} & A^{\text{p}} \end{array}\right]\left[\begin{array}{c} X^{\text{e}}\\ Y^{\text{e}}\\ X^{\text{p}}\\ Y^{\text{p}} \end{array}\right]=\;\Omega \left[\begin{array}{cccc} 1 & 0 & 0 & 0\\ 0 & -1 & 0 & 0\\ 0 & 0 & 1 & 0\\ 0 & 0 & 0 & -1 \end{array}\right]\left[\begin{array}{c} X^{\text{e}}\\ Y^{\text{e}}\\ X^{\text{p}}\\ Y^{\text{p}} \end{array}\right].$$ (30)

The matrices **A**^e^, **B**^e^, **X**^e^, and **Y**^e^ are analogous to the orbital Hessians (**A** and **B**) and response amplitudes (**X** and **Y**) that appear in conventional LR-TDDFT,^114,115^ albeit with an additional term associated with electron–proton correlation in **A**^e^ and **B**^e^. The quantities **A**^p^, **B**^p^, **X**^p^, and **Y**^p^ are their protonic counterparts. The quantity **C** is a coupling matrix that includes terms associated with electron–proton Coulomb interactions and electron–proton correlation.

NEO-TDDFT predicts proton vibrational excitation energies that are in a good agreement with grid-based reference values for the fundamental vibrational modes.^483,484^ The electronic excitation energies for the lower electronic states are similar to those obtained with conventional electronic LR-TDDFT,^479^ but vibronic mixing is found to impact the electronic excitation energies for some of the higher electronic states.^486^ The Tamm–Dancoff approximation^114^ can be applied to Eq. (30), eliminating the **Y**^e^ and **Y**^p^ amplitudes, though the resulting NEO-TDA method tends to significantly overestimate proton vibrational frequencies.^479^

The NEO-TDDFT, NEO-TDHF, NEO-TDA, and NEO-CIS methods are available in Q-Chem 5 in both restricted and unrestricted versions. The quantum protons are always assumed to be high-spin. These methods provide electronic, proton vibrational, and electron–proton (vibronic) excitation energies.

## VI. MODELING THE ENVIRONMENT

Most chemistry occurs in the condensed phase, and 21st-century quantum chemistry is characterized by a variety of increasingly sophisticated theoretical models to describe the extended environment around a smaller part of the system that is modeled in detail using electronic structure theory. The simplest approach to modeling a solution-phase molecule is to replace vacuum boundary conditions with dielectric continuum boundary conditions.^500,501^ Section VI A highlights some continuum methods that are new in Q-Chem 5, including capabilities for describing solvent effects on spectroscopy (vertical excitation and ionization energies) and for using a continuum model to describe an anisotropic solvation environment, such as an air/water or aqueous/organic interface.

Hybrid quantum mechanics/molecular mechanics (QM/MM) methods represent a higher degree of sophistication that allows the environment to have atomistic structure, although this necessitates sampling over those atomistic degrees of freedom, at increased cost. Available QM/MM functionality, including interfaces with various MM software packages, is described in Sec. VI B. Taking this one step further, one can imagine “QM/QM” methods that describe the environment at a lower but still quantum level of theory. Historically, this was often accomplished via “subtractive” approaches,^502,503^ as pioneered by Morokuma and co-workers in the “ONIOM” scheme,^504^ but more recently there is growing interest in QM/QM embedding schemes that stitch together two levels of theory in a potentially more natural way. For this purpose, Q-Chem contains a version of projection-based embedding^505,506^ that is described in Sec. VI C. Finally, for a homogeneous QM description of a system that is too large to be tackled in a straightforward way, one can turn to fragmentation methods,^507^ a few of which are described in Sec. VI E.

### A. Continuum solvation models

Dielectric continuum models represent a form of implicit solvation that sidesteps configurational averaging over solvent degrees of freedom, as that averaging is contained (implicitly) within the value of the solvent’s static or zero-frequency dielectric constant, *ɛ*_0_. Within quantum chemistry, the oldest of these models are the *polarizable continuum models* (PCMs),^508^ but historically the best black-box solvation models are the “SM*x*” models developed by Cramer and Truhlar.^509^ See Refs. 501 and 510 for a discussion of the similarities, differences, and nuances of these various models. Q-Chem 5 contains a range of these models,^511^ built upon a smooth discretization procedure for the cavity that defines the interface between the atomistic solute and the structureless continuum.^511–515^ This procedure eliminates numerical artifacts such as discontinuities in the potential energy surface, which can appear in some implementations.^511–513^

#### 1. Models for solvation energies

Q-Chem includes the SM8,^516^ SM12,^517^ and SMD^518^ variants of SM*x*, where the “D” in SMD stands for “density”. Of these, SMD is perhaps the most interesting because it uses density-based electrostatic interactions based on a PCM, and is available (with analytic gradient) in arbitrary basis sets. In addition to these models, Q-Chem 5 also includes the “composite model for implicit representation of solvent” (CMIRS) approach, originally developed by Pomogaeva and Chipman,^519–522^ and later modified by You and Herbert.^523^ CMIRS is designed as a less-empirical continuum solvation model and uses dramatically fewer parameters as compared to the SM*x* models, although the trade-off is that it is presently parameterized for only a few solvents. For the important case of aqueous solvation, error statistics (versus experiment) for small-molecule hydration energies Δ_hyd_*G*° are provided in Table I, and these statistics demonstrate that CMIRS outperforms the SM*x* models for ions in aqueous solution. The dataset is the Minnesota solvation database,^518,524,525^ for which the error bars on the single-ion hydration energies are estimated to be ±3 kcal/mol.^525^ This means that the CMIRS model has reached the limit of the accuracy of the experimental data against which all of the models in Table I were parameterized.

**TABLE I.** Mean unsigned errors (MUEs) for hydration energies Δ_hyd_*G*° using continuum solvation models.^a^

		MUE (kcal/mol)		
Dataset^b^	*N*_data_	SM12	SMD	CMIRS
Neutrals	274	1.3	0.8	0.8
Cations	52	3.5	3.4	1.8
Anions	60	3.8	6.3	2.8
All ions	112	3.7	4.7	2.4

^a^ Computed at the B3LYP/6-31G* level, from Ref. 501.

^b^ Minnesota solvation database.^518,524,525^

CMIRS uses an isocontour of the solute’s electron density *ρ*(**r**) to define the cavity surface,^526^ which is therefore defined in terms of a single empirical parameter and is pleasantly free of other parameters such as atomic van der Waals radii. The disadvantage is that the isodensity construction lacks analytic energy gradients, which are available in Q-Chem 5 for SMD. In Q-Chem, the *self-consistent reaction field* problem defined by the continuum model can be iterated to self-consistency with any SCF level of theory. For post-Hartree–Fock methods, the use of solvent-polarized MOs in the subsequent electron correlation calculation affords a “zeroth-order” correction for solvation effects that is probably accurate to within the limitations of the continuum approach itself.^501^

There is significant confusion in the literature regarding terminology for continuum solvation models.^501,510^ PCMs themselves are electrostatics-only models,^501^ which must be augmented with nonelectrostatic contributions (Pauli repulsion, dispersion, cavitation, etc.) in order to model solvation energies. Models for these nonelectrostatic contributions to Δ_solv_*G*° are included as part of the SM*x* and CMIRS solvation models but are *not* included in PCMs. Even relatively sophisticated electrostatics treatments, such as the “integral equation formulation” (IEF-PCM)^508^ and the closely related “surface and simulation of volume polarization for electrostatics” [SS(V)PE] model,^527,528^ are electrostatics-only descriptions of solvation, as is the much simpler “conductor-like screening model” (COSMO),^529,530^ which often affords results quite similar to IEF-PCM and SS(V)PE.^531^ All of these models are available in Q-Chem; see Ref. 501 for a detailed comparison of them. While not appropriate for computing Δ_solv_*G*°, a PCM alone can still be useful for spectroscopic applications, where the frontier orbital energy levels are modified by the dielectric continuum and this is reflected in the computed excitation energies. Application of PCMs to solvatochromic shifts is discussed next.

#### 2. Nonequilibrium models for vertical excitation and ionization

What is the appropriate manner to describe a sudden change in the solute’s electron density, which occurs upon electronic excitation or ionization, within a continuum representation of the solvent? A simple approach is to partition the solvent polarization into “fast” (electronic) and “slow” (nuclear) components and assume that the former responds instantaneously but that the latter is frozen and remains polarized with respect to the initial state.^532–535^ The slow polarization is therefore out of equilibrium with the solute’s electrons, and such approaches are known as *nonequilibrium* solvation models.^501^ Within this approach, the solvent’s frequency-dependent permittivity *ɛ*(*ω*) is modeled using only its *ω* = 0 limit (the static dielectric constant, *ɛ*_0_) and its *ω* → *∞* limit (the “optical” dielectric constant, *ɛ*_*∞*_). The latter is equal to the square of the solvent’s index of refraction ($\varepsilon_{\infty }=n_{\text{ref}}^{2}$), with values in the range *ɛ*_*∞*_ = 1.8–2.5 for common solvents.^501^

For an electronic transition from initial state |Ψ_0_⟩ to final state |Ψ_*k*_⟩, the Schrödinger equation that one would like to solve is

$$\left({\hat{H}}_{\text{vac}}+{\hat{R}}_{0}^{\text{s}}+{\hat{R}}_{k}^{\text{f}}\right)|\Psi_{k}〉=E_{k}|\Psi_{k}〉,$$ (31)

where ${\hat{H}}_{\text{vac}}$ is the vacuum Hamiltonian and ${\hat{R}}_{k}={\hat{R}}_{0}^{\text{s}}+{\hat{R}}_{k}^{\text{f}}$ is the reaction-field operator, partitioned into a “slow” initial-state component ${\hat{R}}_{0}^{\text{s}}$, representing polarization using wave function |Ψ_0_⟩ and dielectric constant *ɛ*_0_, and a “fast” final-state component ${\hat{R}}_{k}^{\text{f}}$, representing polarization using wave function |Ψ_*k*_⟩ and dielectric constant *ɛ*_*∞*_.^501^ The state-specific nature of the Hamiltonian in Eq. (31) is problematic, however.^536^ A simple solution is to treat ${\hat{R}}_{k}^{\text{f}}$ using first-order perturbation theory in a basis of mutually orthogonal eigenstates of ${\hat{H}}_{0}={\hat{H}}_{\text{vac}}+{\hat{R}}_{0}^{\text{s}+\text{f}}$. This has been called the *perturbation theory state-specific* (ptSS) approach to nonequilibrium solvation.^537–539^ When applied to the CIS-like eigenvalue problem that defines LR-TDDFT, the ptSS approach is closely related to the “corrected LR” approach of Caricato *et al.*;^540^ see Ref. 501 for details.

**FIG. 24.**
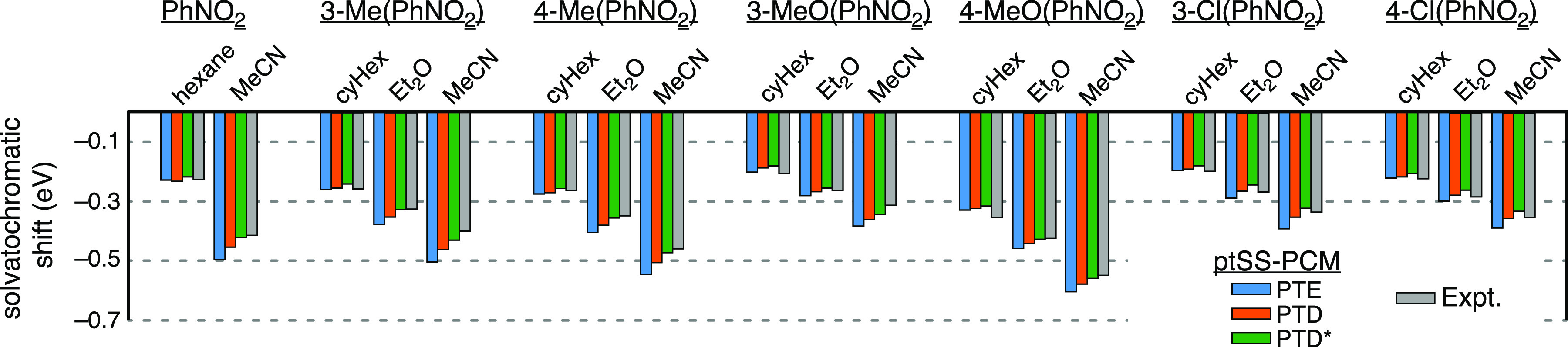
Solvatochromic shifts in the lowest ^1^ππ* state for derivatives of nitrobenzene (PhNO_2_) in different solvents, comparing experimental values to those computed at the ADC(2) level using the ptSS-PCM approach.^538^ The PTE, PTD, and PTD* variants represent slightly different ways of treating the correlated excited-state density.^501,539^ Adapted with permission from Mewes *et al.*, J. Phys. Chem. A **119**, 5446 (2015). Copyright 2015 American Chemical Society.

The ptSS model for solvatochromic shifts is available in Q-Chem 5 for LR-TDDFT^537,538^ and ADC methods.^538,539^ Figure 24 shows some results for a set of nitrobenzene derivatives, with excitation energies computed at the ADC(2) level. The ptSS-PCM solvatochromic shifts compare very well with experiment, and the details of how electron correlation contributions are included in the excited-state density (iteratively alongside the PCM correction or not) matter very little.^539^ In conjunction with LR-TDDFT, the ptSS-PCM approach can also be applied to emission and photoelectron spectroscopies.^537^ In the latter case, nonequilibrium effects of 0.5–1.0 eV on vertical ionization energies (VIEs) have been documented.^541–543^ The nonequilibrium corrections are not yet available for other kinds of excited-state methods (such as EOM-CC), but in those cases, one can still include zeroth-order solvation effects simply by using solvent-polarized Hartree–Fock orbitals in the correlated calculation.

#### 3. Poisson–Boltzmann approach for arbitrary dielectric environments

The solvation models discussed above are designed for the isotropic environment of a bulk solvent, in which case the solvent is characterized by a scalar dielectric *constant* and Poisson’s equation (which defines the continuum electrostatics problem) can be replaced by a more efficient PCM formalism.^501^ However, if the environment is anisotropic (at an interface, for example), then the continuum electrostatics problem is defined instead by the generalized Poisson equation

$$\hat{\nabla }\cdot \left[\varepsilon \left(r\right)\hat{\nabla }\varphi_{\text{tot}}\left(r\right)\right]=-4\pi \rho_{\text{sol}}\left(r\right),$$ (32)

in which *ɛ*(**r**) is an inhomogeneous permittivity *function* and *ρ*_sol_(**r**) is the charge density (nuclei + electrons) of the atomistic solute that is described using quantum chemistry. The solution of Eq. (32) is more expensive than a PCM calculation because it requires discretization of three-dimensional space, but an advantage of the three-dimensional approach is that it provides an exact solution (within the model problem defined by a continuum environment) for the “volume polarization” that arises when the tail of the solute’s charge density penetrates beyond the cavity.^501,544,545^ Equation (32) can also be modified to include the effects of ionic strength (Poisson–Boltzmann equation).^501,546^

**FIG. 25.**
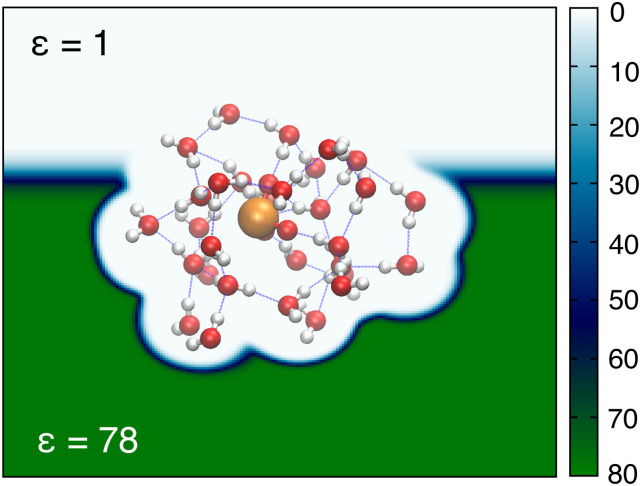
Illustration of an anisotropic permittivity function *ɛ*(**r**) for the air/water interface. The atomistic solute is Cl${\text{O}}_{3}^{-}$(H_2_O)_30_, which amounts to two solvation shells around the ion. Adapted with permission from J. M. Herbert, Wiley Interdiscip. Rev.: Comput. Mol. Sci. **11**, e1519 (2021). Copyright 2021 John Wiley and Sons.

Q-Chem 5 includes a generalized Poisson equation solver (PEqS) for Eq. (32) and the analogous Poisson–Boltzmann equation.^542,546^ For isotropic solvation, *ɛ*(**r**) can be designed to interpolate smoothly across the atomic van der Waals radii, between a “vacuum” value *ɛ* = 1 in the atomistic (quantum chemistry) region and a bulk solvent value outside of that region. A similar construction can be used to obtain a continuum model for the air/water interface,^541–543^ as shown schematically in Fig. 25. Other permittivity models *ɛ*(**r**) have been constructed to describe host/guest systems, where the inside of a molecular capsule screens a guest molecule from the high-dielectric solvent outside, with consequences for the spectroscopy of the guest.^547^

The nonequilibrium ptSS formalism for ionization^537^ (Sec. VI A 2) has also been formulated for use with generalized Poisson boundary conditions,^541,542^ and this ptSS-PEqS approach has been used to compute solution-phase VIEs, including those for ions at the air/water interface.^541–543^ These applications require the use of some explicit water molecules in the atomistic QM region, as shown in Fig. 25. However, whereas aqueous VIEs are notoriously slow to converge, often requiring >500 explicit water molecules,^548–555^ the use of continuum boundary conditions leads to converged results using only about two solvation shells of explicit water.^541,543^ Importantly, only the *nonequilibrium* version of continuum solvation affords VIEs in agreement with experiment.^501,543^ The equilibrium PCM approach may be adequate for *adiabatic* ionization energies but lacks the correct physics to describe vertical excitation or ionization.^501^

### B. QM/MM methods

By itself, Q-Chem contains some limited functionality for QM/MM simulations using standard non-polarizable force fields. This functionality does include periodic boundary conditions for solution-phase QM/MM calculations,^556,557^ and these features have been used to simulate the electronic spectroscopy of aqueous chromophores,^558^ including solvated electrons and other aqueous radicals.^466,557,559–562^ A QM/MM model for physisorption, inspired by dispersion-corrected DFT, is new in Q-Chem 5.^563^

For QM/MM calculations with polarizable force fields, Q-Chem can perform calculations using the *effective fragment potential* (EFP) method,^564^ a QM-derived polarizable force field.^564–566^ QM/EFP calculations can be performed through an interface between Q-Chem and the open-source libefp library.^11,565^ As in previous versions of Q-Chem, QM/EFP calculations are supported at QM levels of theory, including EOM-CC, CIS(D), and LR-TDDFT for excited-state calculations;^567^ in Q-Chem 5, support has been added for ADC/EFP^568^ and for two-photon absorption calculations using EOM-CC/EFP.^569^

Even more flexibility with respect to polarizable force fields is provided by the polarizable embedding (PE) framework,^570^ calculations with which are enabled via an interface between Q-Chem and the open-source cppe library.^14^ PE/SCF calculations are currently enabled for all ground-state SCF methods, and excited-state calculations can be performed at the PE/ADC level.^14^ The latter method has been used to tackle excited states of large biomolecular systems.^571^

For many biomolecular QM/MM applications, it is crucial to have sophisticated tools for visualization and manipulation of coordinates and trajectory data, as well as access to advanced methods for sampling potential energy surfaces. For these purposes, Q-Chem includes interfaces to several popular MM software packages, which serve as front-end drivers to Q-Chem’s computational quantum chemistry engine. An interface to the CHARMM program^572^ has long been a part of Q-Chem,^573^ which can also be accessed via the “CHARMMing” web portal.^574,575^ New in Q-Chem 5 are interfaces to the GROMACS^576^ and NAMD^577^ classical molecular dynamics programs. The GROMACS interface, in particular, supports nonadiabatic trajectory surface-hopping simulations at the CIS and LR-TDDFT levels of theory, including SF-TDDFT (see Sec. II C 2), with GROMACS as the driver for the dynamics. Some tools for “QM-cluster” modeling^578^ of enzyme active sites are also available in Q-Chem itself.^579^

### C. Embedding methods

Taking one step further than QM/MM, one can employ a cost-effective QM theory to describe the environment. The projection-based QM embedding theory^505,506^ provides a robust and formally exact approach to partition a chemical system into two subsystems (*A* and *B*) that are treated at two different levels of QM theory. Typically a small, chemically important part of the system (*A*) is described by a correlated wave function theory (WFT, e.g., MP*n* or CC), while its environment (subsystem *B*) is described using DFT. This scheme goes beyond the electrostatic embedding formalism that is common in ONIOM-style treatments,^504^ as the interaction between the two subsystems is described at the DFT level and is therefore fully quantum-mechanical. Q-Chem 5’s implementation of projection-based embedding supports the use of a myriad of WFT/DFT combinations, thanks to its broad coverage of these two families of electronic structure methods.

A WFT(*A*)-in-DFT(*B*) calculation comprises the following steps:

• Converge the SCF calculation for the full system at the DFT level of theory.
• Partition the occupied orbitals by localizing the canonical MOs and assigning the localized MOs to subsystems *A* and *B*.
• Perform the WFT calculation for the embedded subsystem *A*, which means performing a Hartree–Fock calculation followed by a correlated wave function calculation using the MOs for *A*.

In the final step of this procedure, the MOs assigned to subsystem *B* remain frozen and are employed to construct a projection operator that enforces orthogonality between the MOs of *A* and *B* when the former’s MOs are being re-optimized. Meanwhile, the “environment” subsystem (*B*) affects the QM calculation of *A* by contributing an embedding potential to the one-electron Hamiltonian of *A*, which comprises the Coulomb and XC interactions between two subsystems.

Compared to the original formulation of the projection-based embedding theory,^505^ the implementation in Q-Chem 5 has (i) replaced the use of a somewhat arbitrary level-shift parameter with a strict projection scheme; (ii) implemented the *subsystem-projected atomic orbital decomposition* (SPADE) partition of the occupied space,^580^ which is more robust than the original scheme based on the Pipek-Mezey localization procedure;^581^ and (iii) includes a “concentric localization” scheme to truncate the virtual space with systematically improvable accuracy.^582^ Truncation of the virtual space is essential to reducing the cost of a WFT-in-DFT calculation (relatively to a full WFT treatment), especially for CC methods whose cost increases steeply with the number of virtual orbitals.

Besides the projection-based embedding theory, other notable QM/QM embedding schemes that are available in Q-Chem 5 include frozen-density embedding,^583–587^ embedded mean-field theory,^588^ and the related polarized many-body expansion (MBE) scheme.^589^

### D. Molecules under pressure

Q-Chem includes methods to incorporate the effects of hydrostatic pressure or mechanical forces on molecular structures in geometry optimizations and *ab initio* molecular dynamics simulations. The application of mechanical forces to molecules is modeled by the “external force is explicitly included” approach.^590^ Application of pressure can be modeled either by the hydrostatic compression force field approach,^591^ in which forces point toward the molecular centroid, or via a more refined algorithm, in which mechanical forces are applied perpendicular to the molecular van der Waals surface.^592^ These methods can be deployed in combination with any electronic structure method for which nuclear gradients are available, with no additional computational overhead. Benchmarks show that physically sound geometries are retained even at high pressure.^592^ A more sophisticated approach for applying pressure to chemical systems is the *Gaussians on surface tesserae simulate* *hydrostatic pressure* (GOSTSHYP) algorithm.^593^ This approach uses Gaussian potentials that are distributed evenly on the discretized molecular van der Waals surface to compress the electron density and affords accurate results for energies, structural parameters, dipole moments, and chemical reactions under pressure.^593^ GOSTSHYP energies and gradients are currently implemented only at the SCF level, enabling Hartree–Fock and DFT calculations of compressed atoms and molecules.

### E. Fragment-based methods

Fragmentation methods^507^ seek to sidestep the steep nonlinear scaling of traditional quantum chemistry by sub-dividing a large system into small pieces that can be tackled more tractably by means of distributed computing. Although a plethora of approaches have been discussed in the literature,^507,594^ they are most often implemented at the level of external scripts or driver programs and only a few of them are tightly integrated with Q-Chem itself. A few of these are discussed in the present section, including a general-purpose *n*-body expansion for ground-state energies, an *ab initio* exciton model for representing delocalized excited states in a basis of fragment-localized excitations, and finally a scheme for computing energy-transfer couplings. The energy decomposition methods that are described in Sec. VII can also be considered as examples of fragment-based methods but are discussed separately.

#### 1. Many-body expansion

A simple and straightforward method is the *many-body* *expansion* (MBE),^595–601^

$$E=\underset{I}{\sum }E_{I}+\underset{I<J}{\sum }\Delta E_{IJ}+\underset{I<J<K}{\sum }\Delta E_{IJK}+\cdots \;,$$ (33)

which accounts incrementally for two-body interactions (Δ*E*_*IJ*_ = *E*_*IJ*_ − *E*_*I*_ − *E*_*J*_), three-body interactions (Δ*E*_*IJK*_), etc. Both the MBE and its analytic gradient are available in Q-Chem 5 for ground-state energies of fragments that are not covalently bonded to one another. MBE calculations can be parallelized using either OpenMP (across a node) or MPI, though not both.

Careful analysis of the *n*-body expansion suggests that ostensibly slow convergence is sometimes an artifact of basis-set superposition error (BSSE).^598–600,602–604^ To avoid this, many-body counterpoise corrections are available,^598,599^ which are consistent order-by-order with Eq. (33).

#### 2. *Ab initio* exciton model

The Frenkel exciton model^605^ is an old idea to represent collective, delocalized excitations in multi-chromophore systems using direct-product basis states in which a single monomer is excited,

$$|\Xi_{I}〉=\;\;\overset{\text{monomers}}{\underset{X}{\sum }}\;\;C_{I}^{X}\;|\Psi_{\;A}〉|\Psi_{\;B}〉\cdots |\Psi_{\;X}^{*}〉\cdots |\Psi_{\;N}〉.$$ (34)

The advantage of this “site-basis” is that ground- and excited-state monomer wave functions (|Ψ_*X*_⟩ and $|\Psi_{X}^{*}〉$, respectively) can be computed independently of one another, and applications to very large aggregates are feasible by means of distributed computing.^141^ The model is completed by computing matrix elements between the direct-product basis states, e.g., $\left\langle \Psi_{\;A}^{*}\Psi_{\;B}\Psi_{C}|\hat{H}|\Psi_{\;A}\Psi_{\;B}^{*}\Psi_{C}\right\rangle$, and also the corresponding overlap integrals $\left\langle \Psi_{\;A}^{*}\Psi_{\;B}\Psi_{C}|\Psi_{\;A}\Psi_{\;B}^{*}\Psi_{C}\right\rangle$ because basis functions computed on different monomers are not orthogonal. Addition of higher-lying excited states $|\Psi_{\;X}^{**}〉$ adds variational flexibility to the *ansatz* in Eq. (34), and one solves a generalized eigenvalue problem whose dimension is a few times the number of sites, depending on how many excitations are included per monomer.

Historically, it is common to invoke a dipole-coupling approximation to evaluate matrix elements of $\hat{H}$, and this approximation continues to be made even in modern implementations.^606–608^ The dipole approximation may be satisfactory to describe energy transfer between well-separated chromophores but is questionable under crystal-packing conditions, as in organic photovoltaic materials. The dipole-coupling approximation is not required, and in the *ab initio* Frenkel exciton model developed by Morrison and Herbert,^141,609–611^ these matrix elements are evaluated exactly, within a single-excitation *ansatz* for the monomer excited states,

$$|\Psi_{\;X}^{*}〉=\underset{ia}{\sum }t_{ia}^{X}\;|\Phi_{\;X}^{ia}〉.$$ (35)

Here, $|\Phi_{\;X}^{ia}〉$ represents a singly excited Slater determinant composed of MOs on monomer *X*. This is consistent with either a CIS or a LR-TDDFT calculation for each monomer, incorporating as many individual states $|\Psi_{\;X}^{*}〉$ as desired. In this way, the *ab initio* exciton model can be viewed as a specialized form of nonorthogonal configuration interaction in a customizable diabatic basis.

Using this flexibility, the *ab initio* exciton model has been used to study the singlet fission process in organic photovoltaics,^89,611,612^ meaning the spin-allowed formation of a pair of triplet charge carriers (T + T) via one-photon excitation,

S0→hνS1→fissionsinglet(TT)1→T+T. (36)

The intermediate “multi-exciton” state ^1^(TT), involving triplet states on two different chromophores that are spin-coupled to a singlet, is challenging to describe using standard quantum chemistry because it involves a true double excitation,^613,614^ and such states are absent from conventional LR-TDDFT.^194^ Within the *ansatz* in Eq. (34), however, the ^1^(TT) state simply involves a pair of single excitations with appropriate Clebsch–Gordan coefficients to couple them.^89,612^ The importance of charge-transfer excitons can be interrogated as well, simply by including basis states $|\Psi_{\;A}^{\pm }\Psi_{\;B}^{\mp }\Psi_{C}〉$ involving ionized monomers.^612^ In this way, the *ab initio* exciton model allows one to construct a tailored diabatic basis, letting Schrödinger’s equation decide which basis states are important. Calculations on cluster models of crystalline pentacene have helped to resolve a long-standing debate about the presence of charge-separated states in the low-energy optical spectrum of this material.^89^

Analytic derivative couplings ⟨Ξ_*I*_|(*∂*/*∂x*)|Ξ_*J*_⟩ between excitonic states are also available.^611^ The key ingredient in these couplings are derivatives of the matrix elements of $\hat{H}$ in the exciton site-basis, e.g.,

$$H_{\;AB}^{\left[x\right]}=\frac{\partial }{\partial x}\left\langle \Psi_{\;A}^{*}\Psi_{\;B}\Psi_{C}\left|\hat{H}\right|\Psi_{\;A}\Psi_{\;B}^{*}\Psi_{C}\right\rangle .$$ (37)

**FIG. 26.**
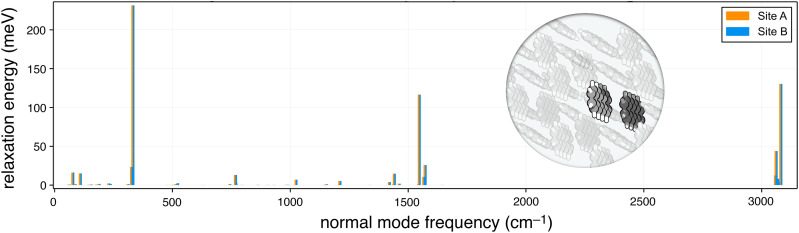
Holstein coupling parameters for crystalline tetracene, obtained from an *ab initio* exciton calculation of $H_{\;AB}^{\left[x\right]}$ for the unit cell projected onto phonon modes from a periodic DFT calculation. The couplings are plotted as relaxation energies $g_{\;AA\theta }^{2}/2\omega_{\theta }$, where *ω*_*θ*_ is the phonon frequency, and indicate several modes that strongly modulate the site energies. Peierls couplings for this system are several orders of magnitude smaller; see Ref. 611. Adapted from A. F. Morrison and J. M. Herbert, J. Chem. Phys. **146**, 224110 (2017) with the permission of AIP Publishing.

Following a transformation from nuclear Cartesian coordinates to normal modes (*x* → *Q*), the quantities $H_{\;AB}^{\left[Q\right]}$ are essentially the linear exciton–phonon coupling parameters *g*_*ABθ*_ that appear in the phenomenological Holstein–Peierls Hamiltonian.^615^ The diagonal coupling parameters *g*_*AAθ*_ are the “Holstein couplings” that describe how the site energies are modulated by phonons *θ*, whereas the off-diagonal couplings *g*_*ABθ*_ are the “Peierls couplings” that quantify how the energy-transfer integrals *H*_*AB*_ are coupled to the phonons.^611^ Often these are treated as phenomenological parameters, but the *ab initio* exciton model affords a means to compute them from first principles. This can be used for *a priori* identification and characterization of the vibrational modes that couple strongly to excitation energy transfer (EET). An example is shown in Fig. 26 for crystalline tetracene, a singlet fission material, where the *ab initio* exciton model identifies several localized vibrational modes on the tetracene monomers that strongly modulate the energy-transfer dynamics.^611,612^

#### 3. Excitation energy transfer couplings

The *ab initio* exciton model described above represents one means to compute EET couplings, but alternative methods exist.^616^ One of these is the fragment excitation difference (FED) scheme, an extension of the fragment charge difference (FCD) method.^617^ In the FED approach, the charge density difference in FCD is replaced by an excitation difference density operator (i.e., the sum of electron and hole densities created upon excitation). Within a single excitation theory such as CIS, one can easily obtain analytic expressions for the matrix elements of the excitation density. However, for multi-excitation wavefunctions, no simple expressions exist for the off-diagonal elements. To circumvent this problem, a new scheme was developed known as *θ*-FED.^618,619^ In this approach, the diabatic states are assumed to be functions of a mixing angle *θ*; thus, the difference density **Δx** depends on *θ* as well. In order to obtain “ideal” diabatic states, the angle *θ* is scanned from −*π*/4 to *π*/4 in order to maximize the difference of the excitation,

$$\theta_{\max}=\underset{-\pi /4<\theta <\pi /4}{argmax}\left\|\Delta x_{i}\left(\theta \right)-\Delta x_{f}\left(\theta \right)\right\|,$$ (38)

with *i* and *f* indicating the initial and final diabatic states. The corresponding *θ*-dependent coupling can then be written as

$$V_{\theta -\text{FED}}=\frac{1}{2}\left(E_{m}-E_{n}\right)\sin\left(2\theta_{\max}\right),$$ (39)

where *E*_*m*_ and *E*_*n*_ are the excitation energies for the two adiabatic states in question.

For wave functions consisting only of single excitations, it has been demonstrated that this generalized *θ*-FED scheme provides results identical to the original FED,^618^ but the former can be extended beyond CIS. In Q-Chem 5, the *θ*-FED scheme is implemented for both CIS and XCIS,^620^ as well as RAS-CI.^285^

## VII. ANALYSIS

Q-Chem offers numerous tools to aid interpretation of *ab initio* calculations and to provide conceptual insights. Some of the more popular ones include natural bond orbital (NBO) analysis,^621^ along with wave function (orbital and density matrix) analysis,^88,269,622^ provided by the libwfa module.^15^ Some recent applications of these tools have been highlighted in Sec. III B, so the present section will focus specifically on a different topic, namely, methods for *energy decomposition analysis* (EDA).

Successful quantum chemistry calculations are akin to numerical experiments, whose physical or chemical interpretation remains a separate problem. To address this problem in the context of intermolecular interactions, EDA methods seek to partition the intermolecular interaction energy between a collection of molecules (or “fragments,” as in Sec. VI E) into physically meaningful components. Two separate approaches for intermolecular EDA are available in Q-Chem 5, one based on variational minimization with constraints, via absolutely localized MOs (the ALMO-EDA scheme,^623^ Sec. VII A), and another based on symmetry-adapted perturbation theory (SAPT),^624^ as described in Sec. VII B.

### A. ALMO-EDA method

The ALMO-EDA scheme identifies contributions to the intermolecular interaction energy by performing variational minimization of the supramolecular DFT energy in the presence of constraints that first prevent polarization and charge transfer (CT), then prevent only CT, and finally with all constraints released. The total DFT interaction energy for a collection of fragments *F*,

$$\Delta E_{\text{INT}}=E_{\text{FULL}}-\underset{F}{\sum }E_{F},$$ (40)

is partitioned according to

$$\Delta E_{\text{INT}}=\Delta E_{\text{GD}}+\Delta E_{\text{FRZ}}+\Delta E_{\text{POL}}+\Delta E_{\text{CT}}.$$ (41)

The geometric distortion energy (Δ*E*_GD_ ≥ 0) is the penalty to distort the fragments from their isolated structures to the geometry of the intermolecular complex. The frozen energy change (Δ*E*_FRZ_) is the net effect of permanent electrostatics, Pauli repulsion, and dispersion. Δ*E*_POL_ is the energy lowering due to electrical polarization (constrained to prevent charge delocalization). Finally, Δ*E*_CT_ is the stabilization due to electron delocalization from one fragment to another,^625^ which is automatically corrected for BSSE in Q-Chem. Key advantages of the variational supramolecular approach include (i) immunity from any convergence questions of perturbation theory and (ii) the ability to select the best density functional for the problem at hand (the theory is applicable, in principle, to the exact density functional, though sadly, it remains unavailable).

Q-Chem 5 contains the latest (second-generation) version of the ALMO-EDA,^626,627^ which includes several significant improvements over the original version.^628,629^ A detailed discussion of the theory can be found elsewhere,^623^ but the following two major improvements warrant specific mention:

1. The polarization energy is defined in a new way that is largely independent of details of the atomic orbital basis set and has a useful complete-basis limit. Intra-fragment relaxation of the frozen orbitals is accomplished by allowing them to mix with fragment-specific electric response functions (FERFs).^630^ These are the virtual orbitals that exactly describe the linear response of the frozen orbitals to uniform electric fields (which requires three dipolar FERFs per occupied orbital) and the spatial gradients of those fields (which requires an additional five quadrupolar FERFs per occupied orbital). The mixing between frozen orbitals and FERFs on each fragment minimize the energy of the complex subject to the constraint of no charge flow between fragments, using the SCF for the molecular interactions (SCF-MI) procedure.^631^
2. The frozen energy change can be decomposed into contributions from its three underlying components: permanent electrostatics, Pauli repulsion, and dispersion.^632^ The dispersion contribution is separated with the aid of a “dispersion-free” density functional, e.g., Hartree–Fock theory in the case that an RSH functional such as *ω*B97X-V or *ω*B97M-V is used to compute *E*_FULL_. Electrostatics can be separated using the traditional quasi-classical definition of the electrostatic interaction between isolated fragments, and what remains is identified as Pauli repulsion.^633^ This traditional approach may be appropriate for force field assessments because fragment densities do not change as the complex is rearranged, but a quantum-mechanically correct alternative definition is also available, wherein the fragment densities deform so as to sum to the total frozen density.^632^

The well-behaved separation of an interaction energy into physically interpretable contributions has permitted use of the ALMO-EDA to assess polarizable force fields^633,634^ and, recently, to develop a highly accurate polarizable force field for water.^635^

**FIG. 27.**
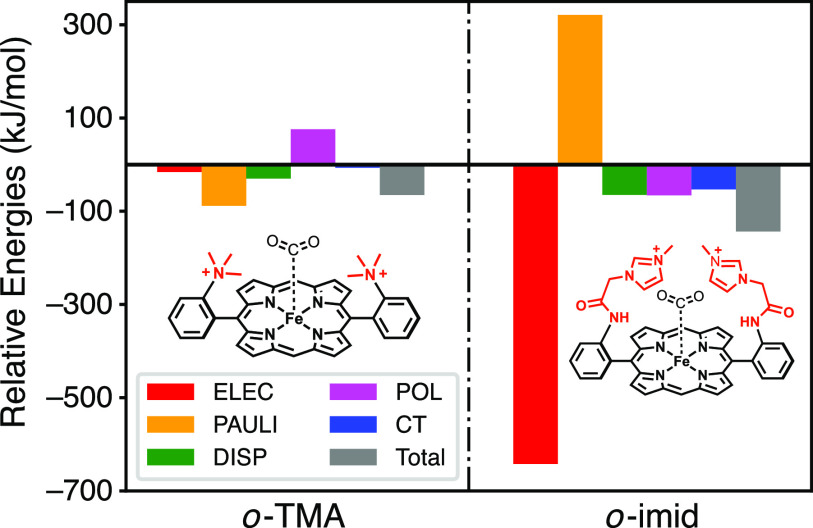
ALMO-EDA(solv) results for the additional binding of CO_2_ when two positively charged substituents (tetramethylammonium, TMA, and an imidazolium-carrying group denoted as “imid”) are introduced at the ortho position of the meso-phenyl group in FeTPP, a promising molecular catalyst for CO_2_ reduction. Compared to unsubstituted FeTPP, the *o*-TMA groups stabilize CO_2_ mainly by alleviating the Pauli repulsion between CO_2_ and the FeTPP core, while the *o*-imid groups stabilize CO_2_ primarily through attractive Coulomb interactions. The solvent is acetonitrile (modeled using C-PCM with *ɛ* = 35.88), and the calculations were performed at the *ω*B97X-V/def2-TZVPP level of theory.^636^

An important new capability is that ALMO-EDA is properly integrated with Q-Chem’s polarizable continuum models (PCMs) of solvent,^511–513^ specifically C-PCM and IEF-PCM, which are electrostatics-only, and also SMD^518^ (see Sec. VI A 1). This ALMO-EDA(solv) model^636^ is a significant new capability because the solvent can exert both qualitative and quantitative effects on the binding of a complex. For example, electrostatic interactions may be screened by high-dielectric solvents such as water, whose polarity may also permit larger polarization and/or CT interactions by stabilizing the resulting deformed densities. An example of the application of ALMO-EDA(solv) to a CO_2_ reduction catalyst (in acetonitrile solution) is presented in Fig. 27, illustrating the effects of different substituent groups toward stabilizing binding of an activated CO_2_ substrate.^636^

In addition, many useful visualization tools are available in conjunction with ALMO-EDA calculations, including the automatic generation of significant complementary occupied-virtual pairs (COVPs)^629,637^ for characterizing charge transfer between fragments, electron density difference (EDD) plots between different intermediate stages of ALMO-EDA, and its further partition into natural orbitals for chemical valence (NOCV) pairs.^638^ Beyond SCF methods, ALMO-EDA is also available at the MP2 level for both closed- and open-shell reference determinants.^639–641^ Beyond ground states, ALMO-EDA can be used to analyze excited states of intermolecular complexes (excimers and exciplexes) at the level of either CIS or LR-TDDFT.^642,643^

One of the traditional criticisms of EDA techniques is that the energy components themselves are not observables,^644,645^ so there is some arbitrariness in their definitions. A substantive step to address this issue has been taken with the introduction of an adiabatic EDA (aEDA),^646^ where observable quantities such as structure and vibrational frequencies are computed on the potential energy surface belonging to each constrained energy. These include the frozen energy (*E*_FRZ_), the polarized energy (*E*_POL_), and the individual fragment energies, {*E*_*F*_}, as well as the final unconstrained supramolecular energy *E*_FULL_. This enables calculation of negative semidefinite aEDA energy components,

$$\Delta E_{\text{INT}}=\Delta E_{\text{FRZ}}^{\text{ad}}+\Delta E_{\text{POL}}^{\text{ad}}+\Delta E_{\text{CT}}^{\text{ad}}.$$ (42)

The components in Eq. (42) are given as the energy difference between the optimal structures in each consecutive pair of states. For example, if the optimized structures on the FRZ and POL surfaces are denoted as **R**_FRZ_ and **R**_POL_, then

$$\Delta E_{\text{POL}}^{\text{ad}}=E_{\text{POL}}\left(R_{\text{POL}}\right)-E_{\text{FRZ}}\left(R_{\text{FRZ}}\right).$$ (43)

**FIG. 28.**
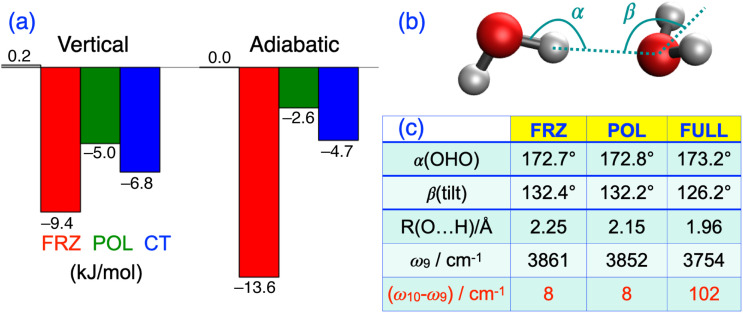
Adiabatic EDA (aEDA) for the water dimer. (a) Comparison of aEDA components vs the conventional (vertical) EDA components. (b) Illustration of the water dimer showing two of the key geometric parameters, whose values at each level of the aEDA are reported in (c). It is striking that linearity of the hydrogen bond is already present at the frozen energy optimization (i.e., it is not critically dependent on polarization or CT) and also striking that the redshift in the proton donor O–H stretch can be directly associated with CT.

Shifts in structures, vibrational frequencies, etc., can be associated with each of the EDA components so that, for example, the difference **R**_POL_ −**R**_FRZ_ demonstrates the effect of polarization on geometry. The example in Fig. 28 illustrates that the redshift of the hydrogen-bonded O–H stretch in the water dimer is primarily associated with CT.

Closely related to the aEDA is the possibility of separately assessing the energetic and observable effects of forward and backward CT, which can be accomplished via a variational forward–backward (VFB) scheme.^641^ The VFB approach uses a generalized SCF-MI method that can disable either forward- or back-donation effects in DFT calculations, thus enabling one to assess the individual role of each, on both the interaction energy but also structure and vibrational frequencies (by performing optimization on the constrained surfaces, as in the aEDA).^646^ This VFB approach is a powerful tool that has been applied to assess the character of a variety of interesting bi-directional metal–ligand interactions, including the novel ligand BF (iso-electronic to CO and N_2_) and also BeO and BeCO_3_ interactions with CO.^641^

**FIG. 29.**
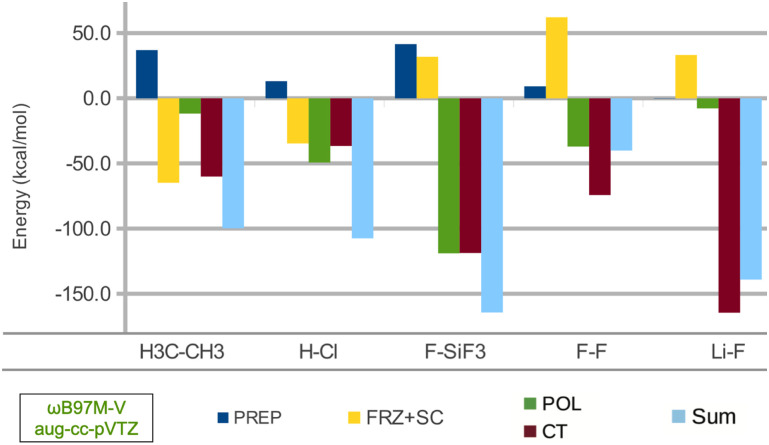
Bond fingerprint in terms of energy components (PREP, FRZ + SC, POL, CT) for several single bonds, showing the contrast between a conventional covalent bond (H_3_C–CH_3_), a polar covalent bond (HCl), the strongest single bond (F_3_Si–F), a charge-shift bond (F_2_), and an ionic bond (LiF). PREP is the generalization of the geometric distortion (GD) energy of Eq. (41) to include electronic hybridization, while the energy lowering due to spin-coupling (SC) between the two radical electrons upon bond-formation is grouped with the frozen (FRZ) energy of Eq. (41).^647^

Finally, the ALMO-EDA can be employed for analysis of single chemical bonds,^647,648^ yielding a fingerprint picture of the chemical bond in terms of energy components. Development of the bonded ALMO-EDA required generalization of the frozen orbital interaction to include the energy lowering associated with spin-coupling of two unpaired electrons, generalization of the geometric distortion term (to become a “preparation energy” that includes the electronic energy cost of hybridizing the orbitals), and finally generalization of the polarization term to include the energy lowering associated with orbital contraction. The latter requires the use of monopolar FERFs.^649^ One interesting use of the bonded ALMO-EDA is to clarify how the fingerprints of exotic chemical bonds compare to those of more familiar bonds, as illustrated in Fig. 29. As one example, the Zn(I)–Zn(I) bond in dizincocene (Cp − Zn − Zn − Cp) emerges as a conventional covalent chemical bond, analogous to H_2_. By contrast, the Mn(0)–Mn(0) bond in (CO)_5_Mn–Mn(CO)_5_ behaves as a charge-shift bond^650^ that is more similar to F_2_ than to H_2_. An interesting recent application of the bonded ALMO-EDA was to investigate the role of kinetic energy lowering in chemical bond formation.^651^ The results are controversial because in contrast to the decrease in kinetic energy upon spin coupling in H_2_ (as a result of greater electron delocalization), the bonded EDA shows that kinetic energy rises upon spin-coupling to make covalent single bonds such as H_3_C–CH_3_ due to Pauli repulsion with core electrons.

### B. Symmetry-adapted perturbation theory

Symmetry-adapted perturbation theory (SAPT) offers an alternative kind of EDA for intermolecular interactions, which is at the same time designed for accurate calculation of interaction energies.^624,652,653^ Unlike supramolecular calculations, the interaction energy *E*_int_ is not computed by the energy difference [as in Eq. (40)] and SAPT is therefore free of BSSE. Instead, *E*_int_ is computed directly from perturbation theory, using isolated-monomer wave functions as an unperturbed basis, in a manner that naturally partitions into physically meaningful components, including electrostatics, Pauli repulsion (“exchange”), induction, and dispersion. Through second order in the perturbation, which includes both intermolecular Coulomb operators and the antisymmetrizer that brings in Pauli repulsion, this affords

$$E_{\text{int}}^{\text{SAPT0}}=E_{\text{elst}}^{\left(1\right)}+E_{\text{exch}}^{\left(1\right)}+E_{\text{ind}}^{\left(2\right)}+E_{\text{exch-ind}}^{\left(2\right)}+\;E_{\text{disp}}^{\left(2\right)}+E_{\text{exch-disp}}^{\left(2\right)}+\delta E_{\text{HF}}.$$ (44)

Here, *δE*_HF_ is an optional correction to account for higher-order induction based on a counterpoise-corrected dimer Hartree–Fock calculation.^652^ If Hartree–Fock wave functions are used to describe the monomers, then this second-order approach is known as “SAPT0”^653^ because it is zeroth-order in the Møller–Plesset fluctuation potentials, i.e., it neglects monomer electron correlation effects. These can be incorporated using perturbation theory, albeit at rather high cost.^652,653^ A low-cost alternative is to use Eq. (44) in conjunction with Kohn–Sham wave functions for the monomers in a method known as SAPT0(KS), although care must be taken to use functionals with correct asymptotic behavior, else the anomalously small Kohn–Sham gaps wreak havoc with second-order dispersion.^654,655^ As such, SAPT0(KS) should *only* be used in conjunction with tuned LRC functionals.^655^ In Q-Chem 5, this tuning can be performed in an automated way during the SCF calculation via a global density-dependent (GDD) tuning procedure.^135–137^

Missing from Eq. (44) is a CT term because CT is contained within the induction energy in the traditional formulation of SAPT.^656,657^ The two can be separated, in a manner that is well-defined and stable, by using constrained DFT (cDFT) to define CT-free reference states for the monomers.^658–661^ The SAPT0 induction energy,

$$E_{\text{ind}}^{\text{SAPT0}}=E_{\text{ind}}^{\left(2\right)}+E_{\text{exch}-\text{ind}}^{\left(2\right)}+\delta E_{\text{HF}},$$ (45)

can thereby be separated into a part that represents “pure” or CT-free polarization, along with a CT energy that is defined as the energy lowering upon lifting the cDFT charge constraint.

**FIG. 30.**
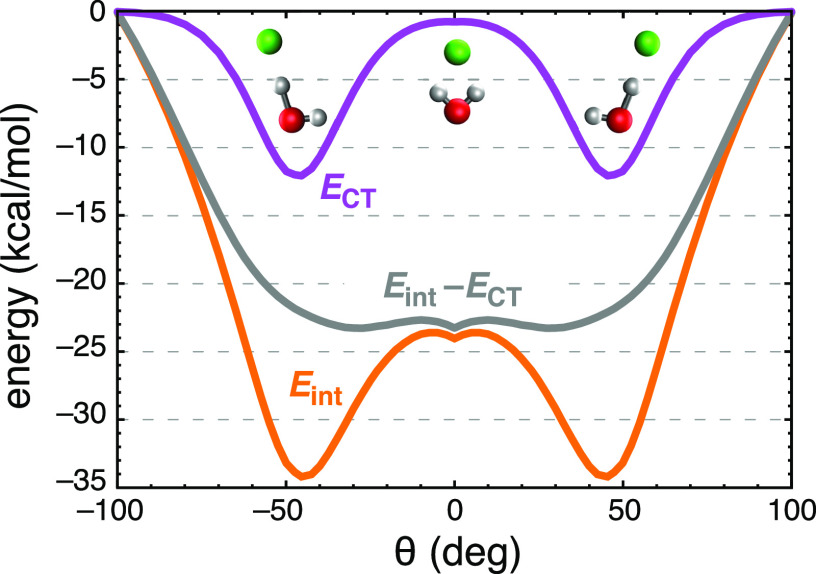
Total interaction potential (*E*_int_) for F^−^(H_2_O) along a relaxed radial scan of the XOH angle, *θ*. Also shown is a SAPT/cDFT-EDA decomposition of *E*_int_ into a CT component (*E*_CT_) and a CT-free interaction energy, *E*_int_ − *E*_CT_. As the ion circumscribes the water molecule, *E*_CT_ turns on sharply in the vicinity of quasi-linear hydrogen bonds. Removal of CT stabilization results in a *C*_2*v*_-symmetric structure, in disagreement with experiment,^662^ although the “dipolar” *C*_2*v*_ structure can still be found in many undergraduate textbooks, e.g., Ref. 663. Adapted with permission from J. M. Herbert and K. Carter-Fenk, J. Phys. Chem. A **125**, 1243 (2021). Copyright 2021 American Chemical Society.

Figure 30 presents an example in which the combined SAPT/cDFT-EDA is used to understand halide–water hydrogen bonding.^661^ Whereas the textbook picture of anion–water interactions imagines a *C*_2*v*_-symmetric structure for X^−^(H_2_O),^663^ with X^−^ at the positive end of the H_2_O dipole moment, gas-phase vibrational spectroscopy convincingly demonstrates the incorrectness of this picture.^662^ According to SAPT/cDFT-EDA analysis,^661^ the existence of quasi-linear hydrogen bonds is driven primarily by CT, which turns on sharply in the vicinity of linear X^−^⋯H–O angles but is negligible at the *C*_2*v*_ “dipolar” geometry.

The SAPT interaction formula in Eq. (44) is traditionally understood to apply to dimers but has been extended to clusters of molecules through a combination with the “XPol” self-consistent charge embedding scheme,^654,664–667^ which is used to capture many-body polarization effects. The combined method, “XSAPT,”^136,624,654,666–672^ is a many-body extension of SAPT that is currently available exclusively in Q-Chem for both closed- and open-shell systems.

Although useful for qualitative and perhaps semiquantitative purposes, second-order SAPT0 is not a benchmark-quality method,^653,669^ primarily due to the limitations of second-order dispersion,

$$E_{\text{disp}}^{\text{SAPT0}}=E_{\text{disp}}^{\left(2\right)}+E_{\text{exch}-\text{disp}}^{\left(2\right)}.$$ (46)

SAPT0 calculations are often performed using a limited basis set such as jun-cc-pVDZ^673^ in order to affect some error cancellation.^653^ An alternative is to seek replacements for $E_{\text{disp}}^{\text{SAPT0}}$, and two such methods are available in Q-Chem:

• XSAPT + *ai*D,^136,624,668,669^ which adds an *ab initio* dispersion potential in place of $E_{\text{disp}}^{\text{SAPT0}}$. Although similar in form to “+D” corrections in DFT + D,^674^ the +*ai*D correction is fitted to pure dispersion data from DFT-SAPT, SAPT2+(3), and SAPT2+3(CCD) calculations, each of which provides CCSD(T)-quality interaction energies but remains separable into components.^652,653^ Taking advantage of the separability of the SAPT interaction energy, XSAPT + *ai*D avoids the double-counting that is inherent in DFT + D.^674^ (As a result, the +D corrections in DFT + D should never be interpreted as genuine dispersion.^37,659^) The third-generation + *ai*D3 correction is the latest and most accurate.^624^
• XSAPT + MBD,^667,672^ which incorporates a modified form^672^ of the many-body dispersion (MBD) model.^72–74^ As compared to XSAPT + *ai*D, this is much closer to a first-principles model and also more accurate.

**FIG. 31.**
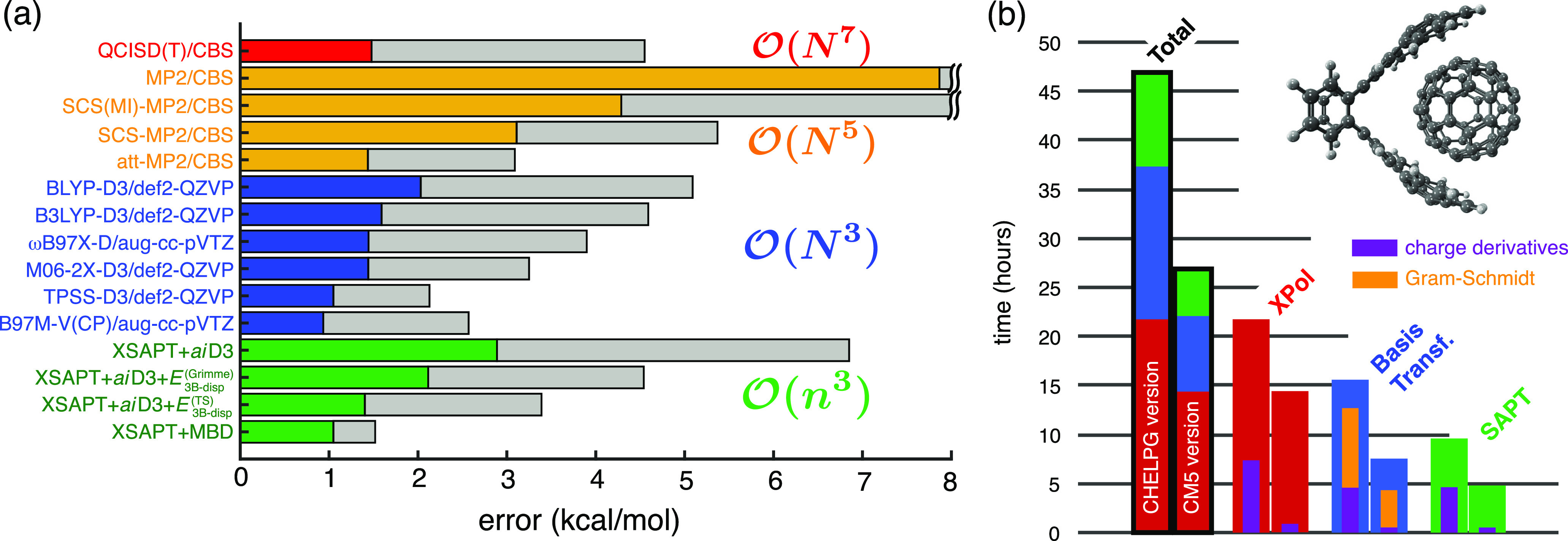
(a) Errors in interaction energies for the L7 set of large dispersion-bound dimers,^675^ as predicted by a variety of quantum-chemical methods in comparison to complete-basis set (CBS) CCSD(T) benchmarks. Gray bars indicate maximum errors whereas colored bars indicate mean absolute errors. The latter are color-coded according to computational cost, with $O\left(N^{p}\right)$ indicating *p*th-order scaling with respect to the size *N* of the supramolecular complex, whereas $O\left(n^{3}\right)$ means cubic scaling with respect to the size *n* of the largest monomer. These comparisons were originally reported in Ref. 672, but the XSAPT + MBD statistics have been updated to reflect modifications reported in Ref. 667. (b) Timing breakdown for an XSAPT + *ai*D calculation of the C_60_ @ C_60_H_28_ “buckycatcher” complex (4592 basis functions) on a single 28-core node. The left bar in each pair uses the original XPol embedding based on ChElPG charges,^654^ and the right bar is a new implementation based on CM5 charges. 667. Panel (a) is adapted with permission from K. Carter-Fenk, K. U. Lao, K.-Y. Liu, and J. M. Herbert, J. Phys. Chem. Lett. **10**, 2706 (2019). Copyright 2019 American Chemical Society. Panel (b) is reproduced from Liu *et al.*, J. Chem. Phys. **151**, 031102 (2019) with the permission of AIP Publishing.

Although designed as intermolecular EDAs, XSAPT methods are also among the most accurate quantum chemistry methods for predicting intermolecular interaction energies, as demonstrated by error statistics for the L7 dataset^675^ [Fig. 31(a)]. MP2-based methods dramatically overestimate these dispersion-dominated interaction energies, with the exception of the “attenuated” att-MP2 method,^676^ which is also available in Q-Chem. The selection of DFT methods in Fig. 31(a) is chosen carefully to focus on those that do well for non-covalent interactions. Hence, it is impressive that XSAPT + MBD approaches the MAE of the best density functional tested, B97M-V, and has lower maximum error. The combination of benchmark-quality energies with a physically meaningful decomposition is one reason that SAPT-based methods are used to parameterize physically motivated force fields.^677^ These desirable properties have also been used to make fundamental inquiries regarding the nature of *π*–*π* interactions.^678,679^ The latter studies demonstrate, for example, that the textbook^680^ Hunter–Sanders (quadrupolar electrostatic) model of *π*-stacking is simply wrong.^678^ The frequently asked question,^681^ “is *π*-stacking a unique form of dispersion?”, can be answered in the affirmative using XSAPT + MBD calculations, although a detailed analysis suggests that stacking is driven by molecular shape rather than by aromaticity *per se*, in what has been called the “pizza-*π*” model of stacking interactions.^679^

**FIG. 32.**
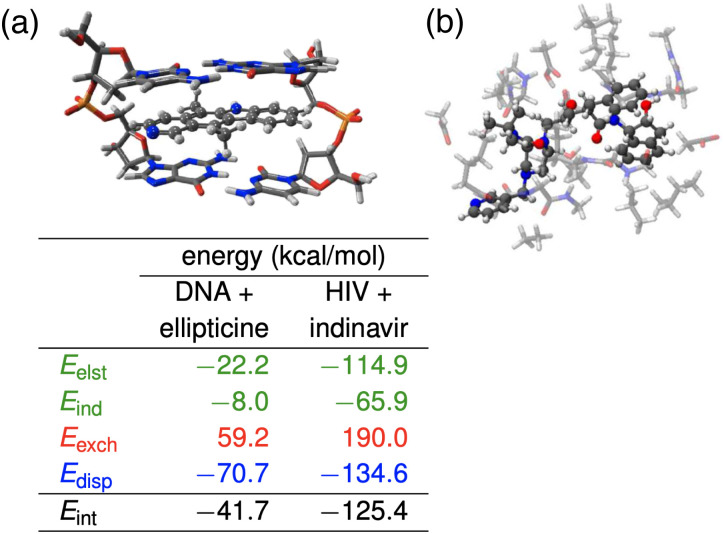
Model systems for drug binding: (a) DNA/ellipticine intercalation complex (157 atoms) and (b) the protease inhibitor molecule indinavir, situated in a model of HIV-2 protease (323 atoms). The table shows XSAPT + MBD energy components from Ref. 667.

Notably, XSAPT calculations are considerably *less* expensive than supramolecular DFT due to the monomer-based nature of XSAPT. For XSAPT + *ai*D and XSAPT + MBD, the rate-limiting step is $O\left(n^{3}\right)$ with respect to the *monomer* size (*n*), rather than the supersystem size. The method can be implemented efficiently in the atomic orbital basis,^136^ and a new XPol embedding scheme based on CM5 charges,^682^ available in Q-Chem 5, offers almost 2× speedup over earlier versions;^667^ see Fig. 31(b). Cost savings relative to supramolecular DFT are most pronounced in systems that can be divided into more than two fragments, such as the DNA intercalation complex that is shown in Fig. 32(a). For this system, a counterpoise-corrected interaction energy calculation at the level of *ω*B97M-V/def2-TZVPPD (4561 basis functions) requires 3 × 13 h on a 40-core compute node, i.e., 13 h for each of the three supramolecular calculations that are needed to compute *E*_int_ = *E*_AB_ − *E*_A_ − *E*_B_. In contrast, an XSAPT + MBD calculation using the same basis set requires 7 × 6 h running on the same hardware.^672^ Like the fragment methods discussed in Sec. VI E (of which XSAPT can be considered an example), these seven constituent calculations can be run independently on different compute nodes.

Figure 32 shows two pharmacologically relevant examples of ligand–macromolecule binding, along with the XSAPT + MBD energy decomposition for each.^667^ One of these is a DNA intercalation complex [Fig. 32(a)], emblematic of *π*-stacking interactions, but the other does not exhibit any obvious dominant binding motif yet has a dispersion energy that is almost twice as large as that of the DNA intercalation complex. In the HIV + indinavir system, which is considerably larger, dispersion arises from a large number of small contributions that must be treated carefully. Notably, for the DNA/ellipticine complex, the XSAPT + MBD interaction energy (reported as −40.7 or −41.7 kcal/mol, depending on the details of the charge embedding^667^) is in better agreement with the complete-basis CCSD(T) benchmark (−38.6 ± 2.2 kcal/mol^683^) than an earlier quantum Monte Carlo estimate (−33.6 ± 0.9 kcal/mol^684^).

## VIII. SOFTWARE ENGINEERING

This article focuses primarily on the diverse scientific advances made by the research groups that comprise the Q-Chem developer community. Figure 2 is a convincing demonstration of sustained energetic growth of the software and the developer community over the past 10+ years. Despite its age, the Q-Chem software shows no signs of aging.

As a software development platform, Q-Chem comes with many challenges for developers and maintainers. Many features are contributed by novice coders without much prior training for whom Q-Chem is their first software development project. This is coupled with an enormous body of computer code that no single person can fully grasp. Software developed by scientists is often notorious for its poor quality assurance and software engineering practices,^685–688^ but Q-Chem developers benefit from the network effect and the stability that the Q-Chem platform provides. The Q-Chem core team and experienced developers provide training and assistance to new community members. Events such as regular developer workshops and webinars, visits to the Q-Chem office in California, and a “Summer at Q-Chem” program facilitate networking, encourage cross-pollination, and help to integrate new developers.

Below, we describe some of the software engineering practices that help to maintain productivity with such a large group of developers.

### A. Software development environment

The Q-Chem code began in the early 1990s as a set of individual components that communicated through temporary files. These components were soon linked together for better performance (by avoiding file-based communication involving large amounts of data), becoming a monolithic code, but while this new structure delivered performance gains, it became difficult to read and maintain over time. The problem is easy to recognize, but the optimal solution is far from obvious. Should we give up, abandon the legacy code, and rewrite the software from scratch? Or should we continue to develop around the old infrastructure and simply adjust to its idiosyncrasies? Following a discussion among the developers, around 2003, a decision was made to pursue slow modernization: continuous code refactoring, gradual rewriting, and quick adoption of newly created component replacements. This strategy has proven to be effective, and Q-Chem’s code has undergone significant improvement while continuing to serve the computational chemistry community. One by one, legacy modules are rewritten and replaced by modern versions with improved performance and enhanced capabilities. Importantly, this process simultaneously preserves the rich functionality of the software, which is essential for applications, while providing a platform for developing new features.

Many Q-Chem developers now choose to begin working on new capabilities within development packages, i.e., small code-development environments with a minimal set of components required to enable a new feature. (The concept is very similar to package management in the context of software development in other languages.) Development packages are very quick to compile and link, which cannot be said of Q-Chem as a whole with its > 10^6^ lines of compilable code. New features are first verified via unit testing and then, following their integration into the Q-Chem package, as end-to-end Q-Chem jobs.

### B. Infrastructure

A small team of software maintainers at Q-Chem provides a number of systems for code and documentation version control, issue tracking, merge requests, continuous integration, and quality control. Q-Chem contributors follow the standard workflow of developing and testing new features, enhancements, and bugfixes on a branch, followed by submission of a merge request. The automated code merge procedure incorporates the changes into the main line of development and executes a suite of pre-commit tests. If any of the tests fails, the merge is rejected and the developer is requested to resolve any issues with assistance from the core Q-Chem team when necessary.

This automated approach provides Q-Chem’s large developer community with assurance that their features will be rolled into release versions in a predictable way. Indeed, Q-Chem software is released on a time-based schedule, with one major release and two minor releases per year. Beyond automation, the Q-Chem developer community is encouraged to interact via an online forum, and typically there is an in-person developer meeting once a year. These mechanisms help to minimize issues that can arise in a sizable developer community over overlapping or even duplicative contributions.

The back-end infrastructure is a complex system that is largely hidden from the developers. It utilizes a combination of open source, proprietary, and in-house software running on premises as well as in the cloud. Continuous integration and deployment is powered by Jenkins equipped with automated pipelines for software builds, testing, benchmarking, and other routine tasks. Version control is provided by Subversion. Software testing and performance benchmarking is automated using CTest, and the results can be visualized with specialized tools. Trac is used as a wiki-based programmer’s reference, issue tracker, and release planning tool.

### C. Third-party components

Q-Chem makes use of several software libraries developed outside of our own developer community. For example, the Armadillo C++ library^689^ provides convenient template-based C++ application programming interfaces for linear algebra. If requested by the user, libecpint (a C++ library for the evaluation of effective core potentials,^690^ based on the Gauss–Chebyshev quadrature) can be used instead of Q-Chem’s internal algorithms.^691^

## IX. HIGH PERFORMANCE COMPUTING

### A. Platforms

Computational quantum chemistry spans a diverse range of myriad calculation types, ranging from exploratory qualitative analysis to high-accuracy calculations based on many-body theory, and furthermore spans a range from large-scale calculations on hundreds of atoms to high-throughput calculations on thousands of small molecules. Different researchers may therefore use Q-Chem in very different modes of operation, and our vision is to provide all of them with a versatile and flexible software engine that can meet these needs. Q-Chem runs effectively on a variety of architectures, from laptops and desktops to leadership-class supercomputers, and is also now available for cloud computing, for which we provide a ready-to-deploy machine image for use on Amazon Web Services. Users can interact with the Cloud via a Linux shell or by using either IQmol or WebMO.

To enable this versatility, we rely on a variety of techniques for reducing the memory footprint of the software using flexible rebalancing tools for disk vs in-core storage and effective shared-memory (OpenMP) parallelization of key software elements, such as integrals and tensors. That said, Q-Chem to date has focused most performance optimization effort on enabling efficient use of mid-scale computing resources for a single job. Leadership computing or supercomputing resources can then be effectively leveraged via workflows (i.e., job-level parallelism). With this in mind, Q-Chem has placed emphasis on OpenMP (shared memory parallel) capabilities and the use of GPU resources associated with a single node. Below, we discuss some recent advances in these capabilities and present example timings.

### B. Improved OpenMP parallel capabilities

OpenMP is a standard paradigm for shared memory parallel computing. Efficient OpenMP parallelism is thus the key to enabling significantly reduced time-to-solution for single jobs using mid-range computing, where the single job can take as much as an entire single node of a computer cluster or the entire resources of a workstation. Typical modern compute nodes consist of 16–64 cores, but nodes with as many as 128 cores are already available. OpenMP parallel capabilities for DFT calculations were already quite good at the time of the review article describing Q-Chem 4,^20^ but progress since that time has been continuous and significant. Below, some representative snapshots of current OpenMP parallel capabilities for DFT and MP2 are reported. Q-Chem also has excellent OpenMP parallel computing capabilities at the CC/EOM-CC and ADC levels, which have been documented elsewhere.^12,13,215,219^

**FIG. 33.**
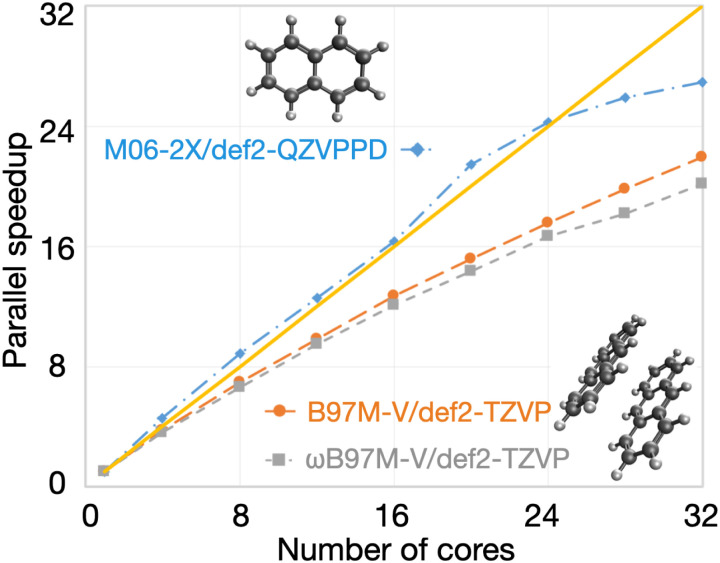
Illustration of OpenMP parallel scaling for DFT calculations. The first example is a single-point energy evaluation in a large basis set (M06-2X/def2-QZVPPD, blue diamonds), as might be performed after structure optimization in a smaller basis set. The other two examples are for the evaluation of the DFT energy and gradient in a triple-*ζ* basis, as often used for geometry optimization. One case is with a semilocal functional (B97M-V/def2-TZVP, orange circles), and the other uses a hybrid functional (*ω*B97M-V/def2-TZVP, gray squares). All calculations were performed on a 32 core dual-socket Intel Xeon CPU E5-2697A server.

OpenMP parallel speedups for DFT calculations are summarized in Fig. 33. For single-point energy evaluation on naphthalene in a large basis (M06-2X/def2-QZVPPD level of theory), it is evident that Q-Chem’s parallel efficiency is very high indeed, with speedups of 16× on 16 cores and 27× on 32 cores. The parallel efficiency is also very good, although noticeably lower, for the two energy + gradient examples in the medium-sized def2-TZVP basis set, performed on the anthracene dimer (C_28_H_20_, 988 basis functions). Using the B97M-V functional, a parallel speedup of 22× is obtained on 32 cores vs 12.7× using 16 cores; the 32-core calculation requires only 516 s of wall time. Energy and gradient evaluation at the *ω*B97M-V/def2-TZVP level of theory exhibits similar scaling. The overhead associated with RSH functionals is not excessive for this calculation: the 32-core job requires 787 s, which is only 50% more than the corresponding pure (semilocal) functional.

**FIG. 34.**
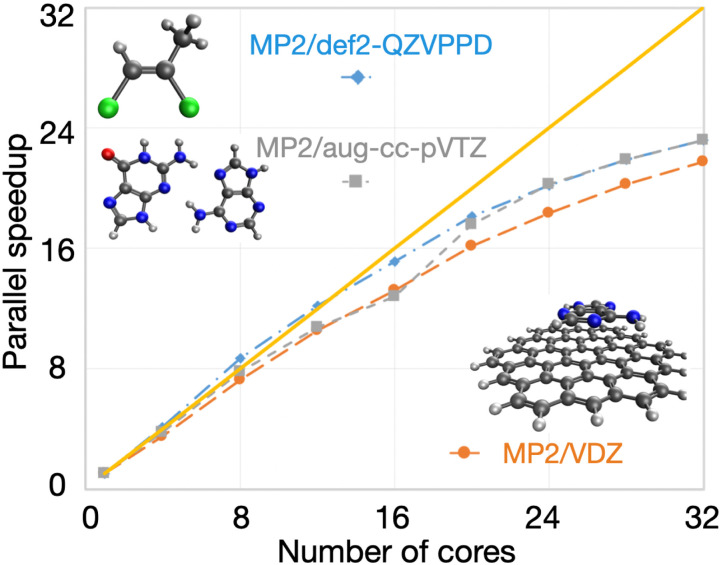
Illustration of OpenMP parallel scaling for evaluation of the MP2 energy and gradient for three molecules: dichloromethyl ethene (C_3_H_4_Cl_2_) with aug-cc-pVQZ,^391,392^ a hydrogen-bonded complex between adenine and guanine (C_10_N_10_H_10_O) in the aug-cc-pVTZ basis,^391,392^ and a circumcoronene complex with adenine (C_59_N_5_H_23_) in the VDZ basis.^692^ The calculations were performed using SCF with exact integrals and MP2 with standard auxiliary (resolution-of-identity or density fitting) basis sets,^693^ with a frozen core approximation. All timings were obtained on a 32 core dual socket Intel Xeon CPU E5-2697A server.

Q-Chem’s new fully object-oriented code for MP2 energies and gradients (as well as the other advanced methods discussed in Sec. III A) requires no storage of amplitudes or four-center electron repulsion integrals and is optimized for OpenMP parallelism. To illustrate the performance of the code, Fig. 34 shows the parallel scaling of the MP2 gradient for three different molecules ranging from 5 to 64 heavy atoms. For all three cases, the results indicate good OpenMP performance all the way out to 32 cores, with speedups of ≈22× (69% parallel efficiency) on 32 cores and somewhat higher efficiency (79%) on 24 cores.

### C. GPU capabilities

A new capability in Q-Chem 5 is the ability to build and diagonalize the Fock matrix using graphics processing units (GPUs). This is achieved through a partnership with StreamNovation Ltd., producers of the BrianQC module,^694^ which functions as an add-on to Q-Chem for the calculation of electron repulsion integrals (ERIs).

ERI computation in Q-Chem exploits a variety of algorithms depending on the properties of the Gaussian basis set, such as the angular momentum classes and the degree of contraction, with an optimal strategy selected based upon the “PRISM” meta-algorithm.^695^ The BrianQC module implements several standard ERI algorithms as well, including McMurchie–Davidson,^696^ Head-Gordon–Pople,^697^ Obara–Saika,^698,699^ and Rys quadrature,^700,701^ and these are controlled by a “BRUSH” meta-algorithm that is optimized for use with GPUs.^702^

In contrast to PRISM and other approaches that were optimized for central processing units (CPUs), the computational power of GPUs is often quite different for single-precision vs double-precision operations, and quantum chemistry integral calculations often require the latter. For that reason, precision and speed requirements are balanced carefully in BrianQC and integrals are evaluated in single or double precision based on a pre-computed strict Cauchy upper bound on their magnitude.^703^ The BRUSH algorithm automatically determines the best possible approach to compute each type of ERI, selecting from among various algorithms and (in the GPU case) between mixed-precision implementations.^702,703^ Each route to ERIs has been implemented and optimized for each supported type of GPU using computer algebra to automatically generate the GPU kernels. (Automatic code generation of this kind is increasingly popular in GPU-based quantum-chemistry code development.^704^) The BrianQC system has its own internal representation for the scalar and tensor expressions that naturally arise in quantum chemistry calculations.

The BrianQC GPU-based ERI engine includes the following features:

• optimization for large molecules;
• support for s, *p*, *d*, *f*, and *g* basis functions;
• support for all NVIDIA GPU architectures (Kepler, Maxwell, Pascal, Volta, and Turing);
• support for 64-bit Linux and Windows operating systems;
• mixed-precision implementation with double-precision accuracy; and
• multi-GPU and supercomputer support.

**FIG. 35.**
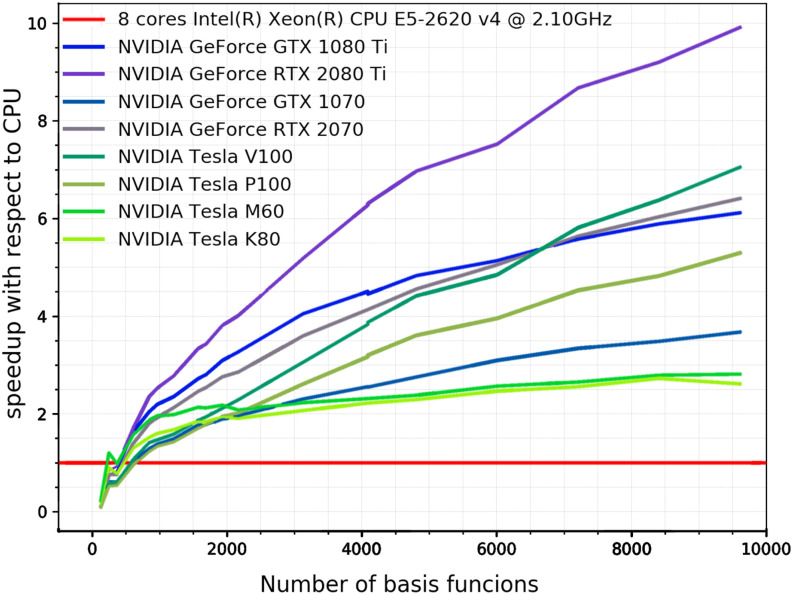
Speedup obtained for single-point B3LYP/cc-pVDZ calculations with BrianQC for randomly generated branched alkanes. Hardware: Intel(R) Xeon(R) CPU E5-2620 v4 2.10 GHz (2 × 8 core); NVIDIA GeForce GTX 1080 Ti, 1070, 980 Ti, RTX 2080 Ti, 2070; Micron 9ASF1G72PZ-2G3B1 DDR4 2400 MHz 8 × 8 GB; ASUS Z10PG-D16 Series Motherboard. For the K80 and M60 GPUs, Amazon Web Service p2.xlarge and g3.4×large instances were used; in the case of P100 and V100 GPUs, Google Cloud instances were used with similar parameters. All CPU timings were obtained with Q-Chem 5.2.2. All GPU timings were obtained using BrianQC 1.0 + Q-Chem 5.2.2.

**FIG. 36.**
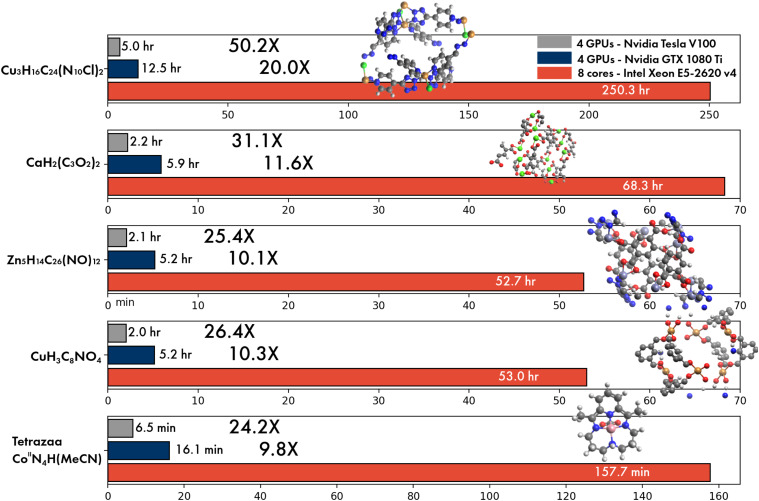
Wall times for DFT (M06-2X/def2-QZVP) energy calculations using Q-Chem with BrianQC. Hardware details can be found in the caption of Fig. 35.

The BrianQC module speeds up every Q-Chem calculation that uses Coulomb and/or exchange integrals and their first derivatives, including Hartree–Fock and DFT energies and geometry optimizations for most functionals. Figure 35 shows speedups vs a CPU-only implementation for B3LYP/cc-pVDZ calculations on a test set of alkanes, and Fig. 36 presents speedups for M06-2X/def2-QZVP calculations on a set of organometallic complexes.

## X. GRAPHICAL USER INTERFACES

Q-Chem jobs can be set up and deployed by WebMO,^705^ a popular web-based interface to quantum chemistry programs, and Q-Chem results can also be visualized using a variety of third-party software, including MolDen, Jmol, and Gabedit. In this section, we focus on two especially fully featured graphical front ends, IQmol^17^ and Spartan.

### A. IQmol visualizer

IQmol is an open-source molecular visualization package^17^ that has been developed within the Q-Chem community and is designed to facilitate the Q-Chem workflow: building molecular structures, generating Q-Chem input files, submitting calculations, and visualizing the results.

Molecular structures can be built from the included molecular library by entering the SMILES ID for simple molecules or by using the free-form builder. Tools are included that enable structures to be quickly optimized using molecular mechanics and to symmetrize geometries to ensure they have the desired point-group symmetry.

Setting up Q-Chem jobs is made easier by an input generator that is aware of the many Q-Chem options and settings and presents these in a hierarchical fashion to avoid overwhelming the new user. Once generated, these inputs can be submitted to either the local machine, a compute server running scheduling software such as PBS or SLURM, or to a freely accessible demonstration server. The latter is a service provided by Q-Chem, Inc. and allows access to Q-Chem’s full functionality, with only a time restriction. This service has been used to great effect in undergraduate and graduate teaching programs in universities around the world.

Results from the Q-Chem output file and associated formatted checkpoint file can be analyzed and visualized in a range of ways depending on the type of calculation. IQmol recognizes and can plot a range of molecular surfaces such as densities and orbitals, including localized orbitals, NTOs, NBOs, and Dyson orbitals. Animations can be generated for vibrational frequencies and pathways, including optimization, intrinsic reaction coordinates, and *ab initio* molecular dynamics trajectories. Visual representations of spectroscopic data are also available, including model spectra for IR, UV, and NMR.

**FIG. 37.**
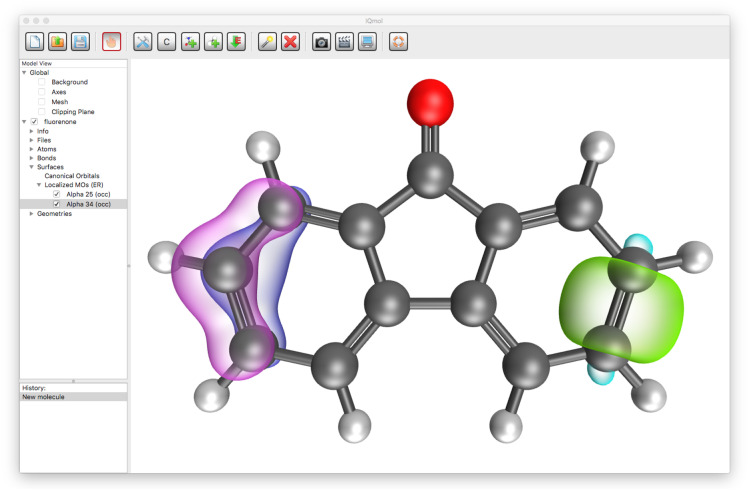
IQmol a provides a convenient front-end and visualization tool for Q-Chem users.

IQmol uses OpenGL shaders to provide a range of appealing and configurable visual effects out of the box, as shown in Fig. 37. In addition, IQmol supports the export of cube file data^346^ and POV-Ray formatted files for import into third-party software for complete control over the appearance of molecular structures and surfaces.

### B. Integration into Spartan

The Spartan program was first introduced in 1991 and since 2000 has provided easy-to-use access to the majority of functionality available in Q-Chem. This includes Hartree–Fock as well as a full range of DFT and wave function-based correlated models, coupled with a wide selection of basis sets. Molecular mechanics models (MMFF and Sybyl) and a selection of semi-empirical models are implemented in Spartan as well.

Multiple molecules (or sets of molecules) may be open in Spartan, and multiple molecules may be submitted to Q-Chem from Spartan. Interface operations and compute tasks are independent. Once a job is “submitted,” either locally or to a remote server, it is marked as “read only,” and the interface is free to deal with other molecules. Upon completion, the job is “unlocked.” Queuing logic allows full control of local and remote resources.

Spartan provides 2D sketching and 3D building tools for organic, organometallic, and inorganic molecules as well as specialized 3D builders for polypeptides and polynucleotides. It also accesses a wide selection of 2D and 3D molecular formats. Guesses for transition states may be obtained with the aid of an internal database by adding “curly arrows” to reactant or product structures. Tools are available for generating regio- and stereoisomers, tautomers, and conformers of flexible cyclic and non-cyclic molecules and for aligning molecules. Job selection (task, method, basis set, and requests for spectra or other properties) is accomplished via simple but open-ended dialogs. Composite tasks (for example), required for the G3 and G4 thermochemical recipes^706,707^ or for the calculation of a Boltzmann-averaged NMR spectrum, are available.

Output for Spartan includes not only text from the Q-Chem output file but also an easy-to-read summary of “important” calculated quantities, e.g., atomic charges and NMR chemical shifts and *J*-couplings. IR, Raman, UV/visible, and NMR spectra (both 1- and 2D) may be plotted and visually compared to experimental spectra. NMR chemical shifts from selected density-functional models may be empirically corrected.

Spartan seamlessly accesses a variety of experimental databases, including the Cambridge Structural Database (CSD) of over a million x-ray crystal structures, the NIST thermochemical database, and the NMR shift database. CSD is under license, while the latter two are freely available. In addition, Spartan accesses the Spartan Structure and Properties database (SSPD), a collection of 300 000 organic and organometallic molecules with *ω*B97X-V/6-311+G(2df,2p) energies obtained at *ω*B97X-D/6-31G* equilibrium geometries and EDF2^708^/6-31G* vibrational frequencies that facilitate calculation of thermochemical quantities (Δ*H*, Δ*S* and Δ*G*). Proton and ^13^C NMR spectra computed at the *ω*B97X-D/6-31G* level are included in SSPD as well. A databases of calculated natural product structures that includes experimental chemical shifts is also provided.

Spartan is released on a two-year schedule with a version number corresponding to the calendar year. The latest version is Spartan’20. Further details about Spartan are available from Wavefunction, Inc.^709^

## XI. CONCLUSIONS AND OUTLOOK

This article has surveyed the broad range of new capabilities developed in Q-Chem over the past six years. Both the author list and the length of this paper itself attest to the strength of the community that has coalesced around contributions to the code. It is this community of developers that has enabled the large majority of the new features and most of the new innovations in methodology reported here. At the same time, support for this community is delivered by a small core group of Q-Chem scientists who have themselves created and tuned critical features, including the substantial modernization of the software development infrastructure to adopt modern best practices of object-oriented programming. This synergy has been critical to the ongoing development of the code: academic developers of Q-Chem have the advantage of using a well-supported infrastructure upon which to build new features, while Q-Chem scientists can focus on commercially critical developments and optimizations. While open source is a powerful movement whose value is unquestioned, the idea that the large community of end users should contribute to the sustainability of the code through a modest purchase price is central to Q-Chem’s approach. However, there is no boundary between the two classes of Q-Chem customers—developers and end-users. It is worth reiterating that anyone or any group that purchases Q-Chem is eligible to join the developer community and help contribute to future advances. We hope that the recent accomplishments reviewed here will inspire future contributions to the code, as well as inspiring myriad chemical applications of this full-featured electronic structure program package.

## DEDICATION

We dedicate this paper to Dr. Michael Wormit and Prof. Nick Besley, whose lives were cut short by tragic accidents in March 2015 and June 2021, respectively. Michael and Nick were dedicated and inspiring members of the Q-Chem family and we remember them as enthusiastic researchers, inspiring teachers, and good friends. We celebrate their important contributions to our community with annual awards: the existing Michael Wormit Award for an outstanding young Q-Chem developer and the newly established Nick Besley Award for contributions to computational spectroscopy in the Q-Chem community.

## Data Availability

The data that support the findings of this study are available within the article.
